# Methanesulfonate in Phosphate Electrolyte Superstructure Enables Ampere-Hour Practical Aqueous Batteries

**DOI:** 10.34133/research.1170

**Published:** 2026-02-25

**Authors:** Jian Zhi, Yunfeng Luo, Chenyi Liao, Zhongyi Liu, Mei Han, Kaihang Yue, Lei Zhang, Guohui Li, P. Chen

**Affiliations:** ^1^ State Key Laboratory of High Performance Ceramics, Shanghai Institute of Ceramics, Chinese Academy of Sciences, Shanghai 200050, China.; ^2^ Department of Chemical Engineering and Waterloo Institute of Nanotechnology, University of Waterloo, Waterloo, Ontario N2L 3G1, Canada.; ^3^ Laboratory of Molecular Modeling and Design, State Key Laboratory of Molecular Reaction Dynamics, Dalian Institute of Chemical Physics, Chinese Academy of Sciences, Dalian, Liaoning 116023, China.; ^4^ Interdisciplinary Research Center for Biology and Chemistry, Liaoning Normal University, Dalian, Liaoning 116029, China.; ^5^ School of Chemical and Biomolecular Engineering, College of Engineering, Eastern Institute of Technology, Ningbo, Zhejiang 315200, China.

## Abstract

Present high-voltage Li-ion aqueous batteries largely rely on highly concentrated (up to 63 *m*) “water-in-salt” electrolyte with limited cycle life (<1,000 cycles) and little hope in practical application. To date, no dilute aqueous electrolyte (salt molality < 2 *m*) can reliably operate beyond 2 V with cycling life over 1,000 cycles. Here, we introduce a “methanesulfonate in phosphate” superstructure that simultaneously tailors Li^+^ migration in molecular crowding solvent and electrode surfaces, which enables an unprecedented electrochemical stability window (up to 4.5 V) and cyclability (up to 10,000 cycles) in a dilute 1.1-*m* aqueous electrolyte. In addition, Such methanesulfonate in phosphate electrolyte can be also applied in various high-voltage electrode couples, and the assembled 60-V, 15-A·h LiMn_2_O_4_/LiTi_2_(PO_4_)_3_ aqueous battery can drive an electric bike for 70 km, highlighting the power of such electrolyte design in the commercialization of high-voltage aqueous batteries.

## Introduction

As lithium-ion (Li-ion) batteries with organic electrolytes are extensively utilized in electronic devices and electric vehicles, there are increasing safety concerns due to their flammability and toxicity, rendering them unsuitable for large-scale energy storage systems [1,2]. Furthermore, the stringent moisture-free processes required for the assembly of these flammable organic electrolytes also incurred substantial manufacturing costs, thereby limiting the global production capacity of Li-ion batteries [3,4]. Replacing flammable organic electrolytes with aqueous electrolytes presents a viable strategy to address the safety issues associated with conventional Li-ion batteries and to reduce manufacturing expenses. Nevertheless, the electrochemical stability window (ESW) of aqueous electrolyte is narrow (1.23 V), which severely constrains the energy density of aqueous Li-ion batteries by limiting the range of electrode couples that can be used [5,6].

Recently, the “water-in-salt” (WIS) concept has introduced a concentrated electrolyte design that expands the ESW of aqueous electrolytes [7,8]. This approach uses a high concentration of 21-*m* lithium bis(trifluoromethanesulfonyl)imide (LiTFSI) [9], extensively utilized in aqueous batteries. The elevated salt concentration fosters the formation of a LiF-based solid electrolyte interface (SEI) through the electrochemical decomposition of the TFSI^−^ [10], thereby extending the ESW of WIS electrolytes to approximately 3 V. Efforts to further widen the ESW include using organic imide hydrate melts of LiTFSI [11], using “water-in-bisalt” (WIBS) electrolytes with additives [12], and increasing the LiTFSI concentration to 63 *m* (mol·kg_solvent_^−1^) [13]. Despite these advancements, economic and environmental concerns continue to restrict the real applications of WIS electrolytes [14]. For instance, LiTFSI salt has limited large-scale capability that is only available in small quantities at a high price of $10 g^−1^ (Sigma-Aldrich, 2022). Moreover, the use of such highly concentrated fluorinated salts normally results in extremely low ion transfer kinetics in LiF-rich interphase [15], leading to limited cycling life (<1,000 cycles ) and poor tolerance under extreme and abusive conditions [16]. To overcome these challenges, alternative strategies to develop a dilute electrolyte with enhanced ion conductions are urgently required to make aqueous batteries feasible in large-scale energy storage systems.

It is well known that dilute electrolytes normally show a low ionic conduction because the scarcity of ions limits total charge transport, which has been often quoted to be detrimental in battery performance [17]. However, we argue the opposite and believe through precise solvent shell reconfiguration; the ion transfer kinetics can still be enhanced in dilute aqueous electrolyte. Inspired by molecular crowding electrolyte design proposed by Xie et al. [18], here, we reported “methanesulfonate in phosphate” (MIP) liquid superstructure that enables increased Li^+^ transference number (*t*_Li+_) and diffusion coefficient (*D*_Li+_) in a dilute (1.1 *m*) lithium methanesulfonate (LiMS) aqueous electrolyte compared to their concentrated counterparts (18 *m*). Such peculiar trend is realized by the synergic superionic pairing between trimethyl phosphate (TMP) and methanesulfonate (MS^−^) anions. In one hand, bulky methyl phosphate groups from TMP spatially shield Li^+^–H_2_O interactions and cut off the widely extended Li–MS interaction network, which leads to an increased degree of uncorrelation between Li^+^ and MS^−^ motions and a higher calculated *t*_Li+_ and *D*_Li+_. In another hand, the oxygen atoms from strongly polarized –SO_3_^−^ of MS^−^ carry a high negative charge and form strong ion–dipole interactions with the H^+^ atoms in H_2_O molecules. Such H_2_O–H_2_O hydrogen bond (HB) disruption results in a smaller and more dynamic solvation shell, faster solvation exchange, and reduced competition from proton conduction—together leading to enhanced Li^+^ transport kinetics.

Meanwhile, we also identified a selective diffusion of Li^+^ over H^+^ at the γ-Li_3_PO_4_ and amorphous Li_3_PO_4_ electrode–electrolyte interfaces derived from such MIP electrolyte superstructure. This unique feature markedly suppresses oxygen and hydrogen evolution in dilute aqueous electrolyte while maintaining excellent Li^+^ transfer kinetics, which enables an exceptional lifespan (up to 10,000 cycles) in full batteries and widened ESW (up to 4.5 V) that even surpasses the ESW (3 V) of concentrated WIS electrolytes. This work is a notable departure from the previously reported LiF-rich electrode–electrolyte interphase based on organic additives and TFSI^−^ anions, which only physically blocks H_2_O to improve interfacial stability in aqueous electrolytes [18–20]. Our MIP electrolyte design also demonstrates a high output voltage (up to 2.5 V) in a wide range of battery systems, including LiMn_2_O_4_ (LMO)/LiTi_2_(PO_4_)_3_ (LTP), LMO/TiO_2_, LMO/Li_4_Ti_5_O_12_ (LTO), LMO/Zn_2_Nb_34_O_87_ (ZnNbO), LiCoO_2_ (LCO)/LTO, LiNi_0.33_Co_0.33_Mn_0.33_O_2_ (NCM_111_)/LTO, and LiNi_0.5_Co_0.2_Mn_0.3_O_2_ (NCM_523_)/LTO. Moreover, MIP aqueous electrolytes also provide wide operating temperature range (−40 to 25 °C) and superior cycling performance (>88% capacity retention after 1,000 cycles) in 2-A·h class pouch battery, demonstrating excellent practical viability. On the basis of such ampere-hour level battery pack, we also assembled 60-V, 15-A·h aqueous battery and drive an electric bike for 70 km, highlighting the potential of MIP electrolyte in replacing lead–acid batteries in low-speed vehicles.

## Results and Discussion

**Fig. 1.**
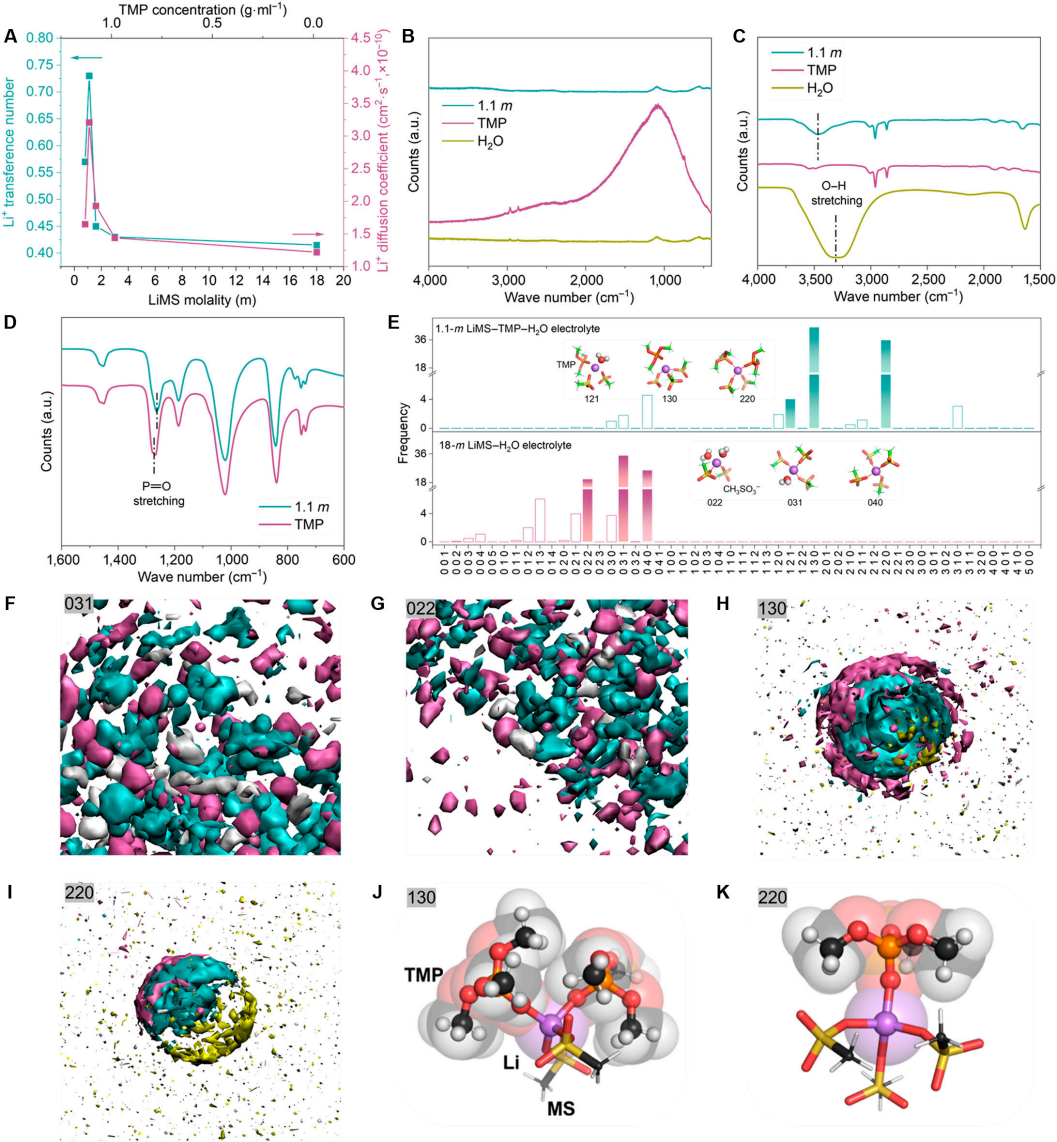
(A) Li^+^ transference number (*t*_Li+_) and diffusion coefficient (*D*_Li+_) of various LiMS–H_2_O electrolytes under different LiMS modalities and TMP concentrations. (B) Raman and (C and D) FT-IR spectra of 1.1-*m* LiMS–TMP–H_2_O electrolyte in comparison with pristine TMP and H_2_O solvent. a.u., arbitrary units. (E) Distribution of coordination structures between Li^+^ and TMP, MS, and H_2_O. (F to I) SDFs of MS (cyan), TMP (yellow), and H_2_O (gray) around a Li^+^ (purple) in (F) 031, (G) 022, (H) 130, and (I) 220 coordination structures. The surfaces represent isosurfaces of normalized concentration for mass center of different components. (J and K) Molecular shielding of umbrella-shaped TMP with LiMS in (J) 130 and (K) 220 coordination structures.

Attributing to the unique nontoxic and water-miscible features of TMP [21,22], in this study, we successfully prepared a series of aqueous MIP electrolytes with TMP concentration of up to 1.13 g·ml^−1^ (Table S2). The ionic conductivity of the electrolyte decreases from 20.3 to 0.9 mS·cm^−1^, as the TMP concentration increases from 0 g·ml^−1^ in 18-*m* LiMS–H_2_O to 1.13 g·ml^−1^ in 0.8-*m* LiMS–TMP–H_2_O (Fig. S1), which is consistent with the general expectations for dilute electrolytes [23]. However, we noted an increase in both transference number (*t*_Li+_) and diffusion coefficient (*D*_Li+_; see Supplementary Discussion and Figs. S2 and S3) in various MIP electrolytes with increasing TMP content (from 18-*m* LiMS–H_2_O to 1.1-*m* LiMS–TMP–H_2_O), peaking at 1.1 *m* and slightly declining upon further TMP increase in the 0.8-*m* electrolyte (Fig. 1A). We also calculate *D*_Li+_ of various MIP electrolytes via pulsed field gradient nuclear magnetic resonance according to the Stejskal–Tanner equation [24]. As shown in Fig. S4, the 1.1-*m* LiMS–TMP–H_2_O electrolyte shows the highest *D*_Li+_ among all MIP electrolytes, which is in good correlation with the value of *D*_Li+_ based on galvanostatic intermittent titration technique (Fig. 1A). The sharp spectrum for 1.1-*m* LiMS–TMP–H_2_O (Fig. S5) indicates the averaging of the dipole and quadrupole couplings due to the isotropic fast Li^+^ transfer kinetics that is similar to the Li^+^ motion behavior in single crystals [25].

Such a peculiar trend in Li^+^ diffusion kinetics implies a boosting effect in MIP electrolyte, which prompts us to investigate the intermolecular solvation structure. The Raman spectra of the 1.1-*m* LiMS–TMP–H_2_O electrolyte, shown alongside those of pure TMP and H_2_O in Fig. 1B, indicate the quenching of fluorescence, facilitated by the excited-state strengthened intermolecular HB between the phosphate groups of TMP and H_2_O molecules [26]. Compared to pure H_2_O, there is a notable blue shift of the O–H stretching vibration bands in Fourier transform infrared (FT-IR) spectra of the 1.1-*m* LiMS–TMP–H_2_O aqueous electrolyte (Fig. 1C). It is due to the higher Gutmann donor number of TMP (23) compared to H_2_O (18) [27], which leads to a deshielding in the solvation sheath of Li^+^. In addition, the red shift of P═O stretch band pairs (1,277 and 1,268 cm^−1^) observed in Fig. 1D suggests the formation of an ionic-liquid-type equilibrium state with reduced activity [28,29], where H_2_O molecules are bonded to the phosphate groups of TMP. It is well known that FT-IR spectra is primarily based on the vibrational modes of covalent bonds such as stretching and bending vibrations, etc. [30]. These vibrational modes absorb infrared radiation and induce transitions between vibrational energy levels, forming characteristic absorption peaks. However, since Li^+^ ions form ionic bonds rather than covalent bonds, they do not contribute to vibrational energy transitions and therefore do not produce absorption peaks in the FT-IR spectra. As a result, the presence of Li^+^ ions does not influence the observed spectra, and there are no obvious differences between the 1.1-*m* LiMS–TMP–H_2_O electrolyte and the TMP–H_2_O solution under the same TMP:H_2_O molar ratio (Fig. S6).

Density functional theory (DFT) characterization was also performed to elaborately scrutinize the interplay among MS^−^ anions and water in MIP electrolyte. As shown in Fig. S7, MS^−^–H_2_O shows a binding energy of 98.26 kJ·mol^−1^, which is more than 4-fold higher than that of H_2_O–H_2_O (23.67 kJ·mol^−1^) and much higher than those of other chaotropic anions (TFSI^−^, BF_4_^−^, and ClO_4_^−^) with water. The HBs of water can be broken by anions through charge-driven formation of anion–H bonding; typically, a higher binding energy indicates stronger bond formation [31,32]. This effect was further examined via ^1^H nuclear magnetic resonance spectroscopy of pure water, 1.1-*m* LiMS–H_2_O and 1.1-*m* LiTFSI–H_2_O (Fig. S8). Notably, the ^1^H peak shifts upfield in the presence of MS^−^ anions, confirming a weakening of the HB in water molecules, which aligns with the DFT calculations. In contrast, the degree of peak shift with TFSI^−^ anion is relatively minor, demonstrating a weak breaking effect of HB in water.

Molecular dynamics (MD) simulations were carried out to further elucidate the solvation structures of 18-*m* LiMS–H_2_O and various MIP electrolytes (3.0-, 1.6-, 1.1-, and 0.8-*m* LiMS–TMP–H_2_O). A snapshot of lithium aggregates from 18-*m* LiMS–H_2_O (Fig. S9) illustrates that one MS coordinates with 3 Li^+^, which are coordinated by surrounding MS and H_2_O to form a wide spreading –Li–MS– matrix with dominant 040, 031, and 022 coordination structures (Fig. 1E and Fig. S10; where “*xyz*” represents the numbers of TMP [*x*], MS^−^ [*y*], and H_2_O [*z*] around one Li^+^ atom). Spatial distribution functions (SDFs) of MS (cyan) and H_2_O (gray) around a Li^+^ in a 040 configuration display a densely packed –Li–MS–H_2_O interaction network (Fig. S11), which becomes slightly scattered in 031 and 022 configurations (Fig. 1F and G). This result is consistent with our radial distribution function (RDF) analysis (Fig. S12), which shows that the RDF intensity for MS^−^ is approximately 3 times higher than that of H_2_O due to the stronger interactions between the negatively charged oxygen atoms in MS^−^ and Li^+^. A similar trend is also observed in MIP electrolytes. As the TMP concentration increases, the RDF intensity of MS^−^ gradually decreases but remains approximately twice that of TMP in the 1.1- and 0.8-*m* MIP systems. Electrolyte (1.1 *m*) predominantly features 130 and 220 ion clusters (Fig. 1E and Fig. S10), and their corresponding SDFs reveal discrete features as a colloidal solution (Fig. 1H and I).

This distinct behavior is attributed to the solvent shell reconfiguration of umbrella-shaped TMP molecules, which spatially shields Li^+^–H_2_O interactions by bulky methyl phosphate groups (Fig. 1J and K) and cuts off the widely extended Li–MS interaction network (Fig. S13). Thus, compared with the extended Li–MS interaction network in the 18-*m* LiMS–H_2_O electrolyte, the 1.1-*m* LiMS–TMP–H_2_O electrolyte exhibits TMP-wrapped ion clusters, leading to an increased degree of uncorrelation between Li^+^ and MS motions (Fig. S14 and Table S3) and a higher calculated *t*_Li+_ (Table S2). Meanwhile, The –PO–CH_3_ structure in TMP also provides a proper nonpolar and polar amphiphilic structure to balance TMP’s solubility in MIP electrolyte by comprehensive interactions with Li^+^ and H_2_O molecules. It should be noted that the *t*_Li+_ in Table S3 is calculated from molecular simulations trajectories based on a concentrated solution theory [33], and the uncertainty for *t*^+^ can be affected by the studied systems, theory approximation, the reference frame (such as center-of-mass choice for the whole box or the solvent), and the thermostat method that was applied during the MD simulations. Despite these variations, the computational data remain consistent with the experimental findings in most respects (Fig. 1A), particularly in demonstrating the higher Li^+^ transference number of the 1.1-*m* LiMS–TMP–H_2_O system compared to the 18-*m* system. In addition, the oxygen atom from O═P of TMP molecules also forms abundant HB with neighboring H_2_O molecules (Fig. S15), substantially constraining the dynamics of H atoms and reducing water activity (Fig. S16). The more positive interaction energies of 130 and 220 structures than those of 040, 031, and 022 (Fig. S17) demonstrate the efficient mitigation of Li–MS interactions by TMP molecules and energetically improve the kinetics of uncorrelated Li^+^ motion. Figure S18A shows the ionic conductivity of 1.1-*m* LiMS–TMP–H_2_O electrolytes from 25 to 70 °C. Notably low activation energy of 0.28 eV was calculated on the basis of Arrhenius equation (Fig. S18B), which is in line with the value of other highly conductive aqueous electrolyte and suggests an efficient Li^+^ transport [34].

The enhanced Li^+^ transfer kinetics in MIP electrolyte increase the probability of Li^+^ in the vicinity of electrode surface, which leads to an interfacial chemistry dominated by the intimate reaction between Li^+^ and TMP. Quantum mechanics (QM) calculations (Fig. S19) reveal that the reduction potential of TMP with Li (3.62 to 3.96 V versus Li/Li^+^) is substantially higher than that of TMP without Li (0.89 to 1.6 V versus Li/Li^+^). Although the standard reduction potential seems much higher than other studied material values [9], it is noted that it highly depends on the reduced products and represents the tendency under the standard condition. A high reduction potential for an initial TMP-derived product promotes rapid formation of a phosphate interface on the anode. Further analysis indicates that TMP–Li exhibits a stronger electron-accepting tendency compared to H_2_O, thereby suppressing H_2_ evolution (2.63 V). This preferential reduction of TMP over H_2_O was also validated by projected density of states (PDOS) analysis for both 18-*m* LiMS–H_2_O and 1.1-*m* LiMS–TMP–H_2_O systems (Fig. S20). In the 18-*m* LiMS–H_2_O system, H_2_O and MS contribute to the lowest unoccupied molecular orbital (Fig. S20A and B), facilitating rapid H_2_O reduction at the anode. In contrast, TMP from the 1.1-*m* LiMS–TMP–H_2_O system causes a downshift of the orbital levels and accounts for the vast majority of the lowest unoccupied molecular orbital; thus, it is predominantly reduced at the anode to form a solvent-derived interphase. In addition, TMP alters the electron density states near the highest occupied molecular orbital levels (Figs. S20C and D and S21), which may induce the formation of a solvent derived interphase on the cathode.

**Fig. 2.**
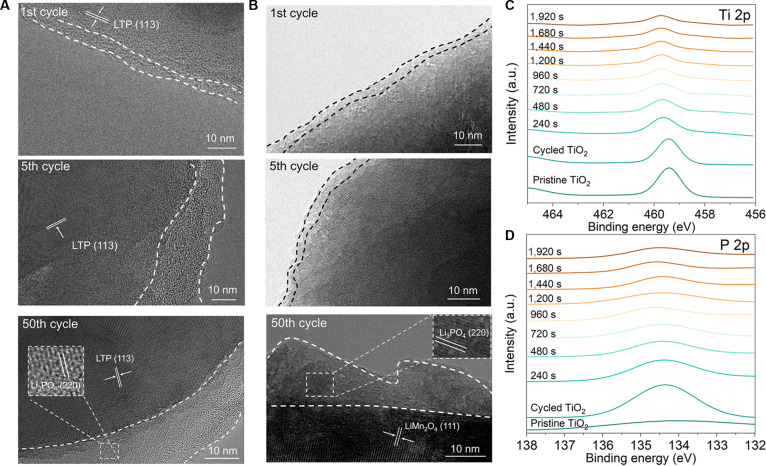
(A) High-resolution cryo-TEM of LTP anode from LMO/LTP battery with 1.1-*m* LiMS–TMP–H_2_O electrolyte after 1st, 5th, and 50th cycles. (B) High-resolution cryo-TEM of LMO cathode from LMO/LTP battery with 1.1-*m* LiMS–TMP–H_2_O electrolyte after 1st, 5th, and 50th cycle. (C and D) XPS spectrum of Ti 2p (C) and P 2p (D) from pristine (bottom) and cycled TiO_2_ anode after various Ar^+^ sputtering durations.

The growth of such interphase derived from MIP electrolyte was further characterized by cryo-transmission electron microscopy (cryo-TEM; Fig. 2A), which clearly illustrates the morphological evolution of the SEI during cycling on an LTP anode from an LMO/LTP battery with the 1.1-*m* LiMS–TMP–H_2_O electrolyte. After 50 cycles, the LTP anode is fully covered with a 20-nm-thick monolithic SEI, whose 2-dimensional lattice fringe matches well with the (220) interplanar spacing of γ-Li_3_PO_4_ (orthorhombic, Joint Committee on Powder Diffraction Standards #15-0760) [35,36]. High-resolution TEM further shows the growth of crystalline/amorphous Li_3_PO_4_ layer during cycling on the LMO cathode (Fig. 2B), indicating the formation of monolithic cathode–electrolyte interphase (CEI). To verify the phosphatic characteristics of this interfacial layer, we performed the x-ray photoelectron spectroscopy (XPS) depth analysis on the SEI layer of LMO/TiO_2_ battery under the 1.1-*m* LiMS–TMP–H_2_O electrolyte (Fig. 2C and D). The most obvious distinction of TiO_2_ anode cycled in this electrolyte is the existence of P arising from TMP. Etching this surface by Ar^+^ shows that the concentration of P diminishes from the surface toward the bulk, implying that phosphorus species are mainly at the top surface of the TiO_2_ anode (Fig. 2D). Closer examination shows a sudden decrease in TiO_2_-related species (Ti 2p) after Ar^+^ etching for 240 s, which remains constant even after prolonged (1,920 s) sputtering (Fig. 2C). Notably, no S 2p signal is detectable in the cycled TiO_2_ (Fig. S22), underscoring a different trend in the changes of P 2p and Ti 2p, which undoubtedly confirm the existence of a monolithic crystalline/amorphous Li_3_PO_4_ interphase on the TiO_2_ anode. Furthermore, energy-dispersive x-ray spectroscopy line scans (Fig. S23) and mappings (Fig. S24) clearly suggest a uniform spatial distribution of P element on the surface of TiO_2_ anode. We wish to emphasize that the identification of Li_3_PO_4_ does not imply it is the sole phosphorus-containing component. In fact, the broad P 2p peak observed in the XPS depth profile, with its peak broadening, suggests the possible coexistence of multiple phosphorus species in diverse chemical environments, which likely includes the organic phosphate decomposition products. TEM results provide further support that while Li_3_PO_4_ was identified in the crystalline regions, amorphous areas could well contain additional components with organic phosphate groups (Fig. 2A and B). These findings collectively reinforce the view that the CEI/SEI constitutes a complex mixture.

**Fig. 3.**
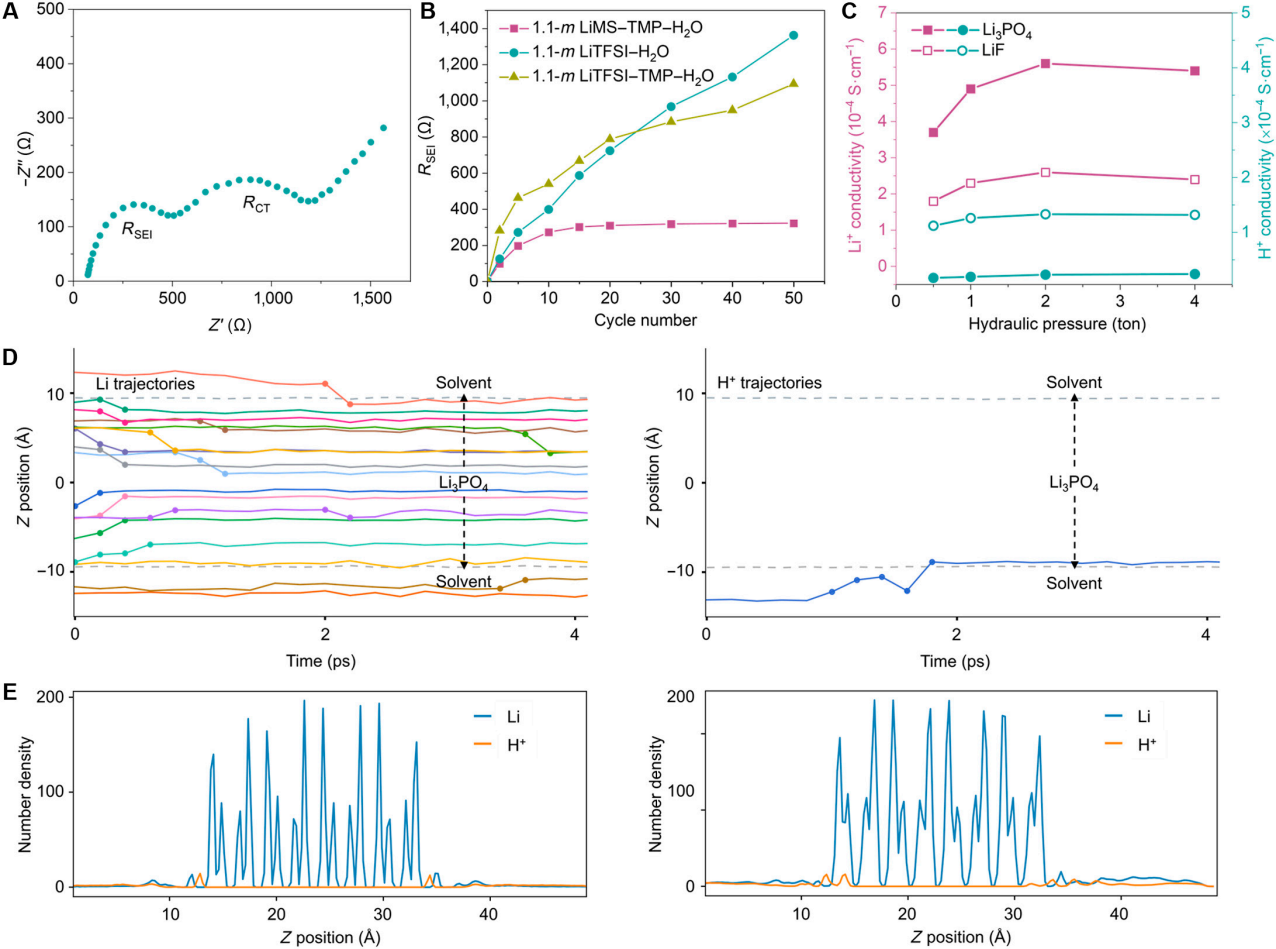
(A) Nyquist plot of TiO_2_ anode from the cycled LMO/TiO_2_ battery (after 50 cycles) with 1.1-*m* LiMS–TMP–H_2_O electrolyte. (B) The change of *R*_sei_ as a function of cycling number of TiO_2_ anode from LMO/TiO_2_ battery with 1.1-*m* LiMS–TMP–H_2_O electrolyte. The change of *R*_sei_ from TiO_2_ anode operated under 1.1-*m* LiTFSI–H_2_O and 1.1-*m* LiTFSI–TMP–H_2_O electrolyte is also shown for comparison. (C) Li^+^ and H^+^ conductivities of γ-Li_3_PO_4_ and LiF pellets under various hydraulic pressures. (D) Illustrations of Li^+^ (left) and H^+^ (right) transport trajectories in γ-Li_3_PO_4_ slabs with vacant Li sites during MD simulations. Each diffusion that is greater than 0.9 Å in 0.2 ps is marked by dots. The positions of the upper and lower interfaces of Li_3_PO_4_ slabs are marked by 2 gray dashed lines. (E) Number density of Li^+^ and H^+^ from 0.25-Å slices on *z* axis in γ-Li_3_PO_4_ slabs without (left) and with (right) vacant Li sites during MD simulations.

To investigate the kinetics of Li^+^ diffusion through such γ- and amorphous Li_3_PO_4_ SEI, we used the electrochemical impedance spectroscopy (EIS) to assess the interfacial resistance of various cycled TiO_2_ anodes in a 3-electrode cell utilizing a 1-*m* Li_2_SO_4_ aqueous electrolyte. The Nyquist plot of the TiO_2_ anode from the LMO/TiO_2_ battery with a 1.1-*m* LiMS–TMP–H_2_O electrolyte after 50 cycles (Fig. 3A) reveals high- and medium-frequency semicircles corresponding to the SEI resistance (*R*_sei_) and charge transfer resistance (*R*_ct_), respectively (Fig. 3A). Utilizing an equivalent circuit model (Fig. S25), the *R*_sei_ of the TiO_2_ anode cycled under the 1.1-*m* LiMS–TMP–H_2_O electrolyte remains stable at approximately 322 Ω after 50 cycles, substantially lower than that observed for TiO_2_ anodes operated under 1.1-*m* LiTFSI–H_2_O and LiTFSI–TMP–H_2_O electrolytes (Fig. 3B). Evidently, the Li_3_PO_4_-based SEI derived from the LiMS–TMP–H_2_O electrolyte exhibits more consistent Li^+^ conduction compared to the fluorinated SEI formed in LiTFSI-based electrolytes. In addition, a higher Li^+^ and lower H^+^ conductivity in γ-Li_3_PO_4_ compared to LiF pellets under various hydraulic pressures (Fig. 3C) suggests faster Li^+^ and slower H^+^ conduction in Li_3_PO_4_ compared to LiF.

To further examine whether the Li^+^/H^+^ conduction selectivity observed in pellet samples can be transferred to thin interphases, we fabricated a series of ultrathin γ-Li_3_PO_4_ and LiF pellets with thicknesses down to 350 μm. As shown in Fig. S26A, all ultrathin γ-Li_3_PO_4_ pellets exhibit higher ionic conduction for Li^+^ than for H^+^. In contrast, all micrometer-scale LiF pellets display comparable Li^+^ and H^+^ ionic conductivities across the entire thickness range (Fig. S26B), which is consistent with the results obtained from millimeter-scale LiF pellets (Fig. 3C). These results indicate that, within the pellet thickness range down to 350 μm, the Li^+^/H^+^ selectivity of γ-Li_3_PO_4_ and LiF is insensitive to thickness under our testing conditions. To better approximate the space charge environment and interfacial conditions encountered during real battery operation, we first attached all γ-Li_3_PO_4_ and LiF pellet samples to glass fiber separators wetted with 1.1-*m* LiMS–TMP–H₂O and LiTFSI–H₂O electrolytes prior to the conductivity measurements. As a result, these measurements demonstrate the intrinsic Li^+^/H^+^ conduction preference of γ-Li_3_PO_4_ and the nonselective ionic transport behavior of LiF under electrolyte-wetted conditions, thereby providing strong experimental support for understanding the ionic selectivity of γ-Li_3_PO_4_- and LiF-based interphases formed from LiMS–TMP–H₂O and LiTFSI–H₂O electrolytes. While nanoscale interphases may additionally be influenced by space charge effects and interfacial microstructural features, the present results establish a reliable bulk reference for interpreting Li^+^/H^+^ transport behavior in the corresponding nanometer-scale interphases.

The selective ionic conduction of Li^+^ over H^+^ facilitated by the Li_3_PO_4_ interphase impedes the migration of H^+^ to the bulk electrode, thereby suppressing H^+^ from accepting electrons and subsequently inhibiting H_2_ evolution. This conclusion is further supported by the analysis of hydrogen evolution reaction (HER) via in situ gas chromatography during the charge–discharge process of LMO/TiO_2_ full batteries. As depicted in Fig. S27, LMO/TiO_2_ batteries with a 1.1-*m* LiMS–TMP–H_2_O electrolyte exhibit the lowest H_2_ evolution rate of 0.27 μmol·h^−1^ during cycling at 5 C, whereas those with 1.1-*m* LiTFSI–H_2_O and LiTFSI–TMP–H_2_O electrolytes display H_2_ evolution rates of 0.98 and 0.87 μmol·h^−1^, respectively.

MD simulations on γ-Li_3_PO_4_ and amorphous Li_3_PO_4_ slabs, with and without vacant Li sites, were conducted to investigate the preference of Li^+^ over H^+^ diffusion tailored by the Li_3_PO_4_ interphase. Rapid Li^+^ transport between vacant sites was observed in both γ-Li_3_PO_4_ (left of Fig. 3D) and amorphous Li_3_PO_4_ (left of Fig. S28) during “vacancy refilling” processes (Movie S1), while only proton trapping by phosphate groups at the interface was observed for H^+^ (right of Fig. 3D and Fig. S28). Rapid Li^+^ transport occurs at the initial stages of a “vacancy refilling” process, slowing down as the potential difference diminishes with vacant sites refilled. Figure S29 illustrates the observed Li^+^ transport pathway between vacant sites in γ-Li_3_PO_4_, wherein Li^+^ was capable of hopping from one vacant site to the next (approximately 3 Å) within 0.2 ps (at a rate of 15 Å·ps^−1^), accompanied by interstitial motions. Figure S30 illustrate MD-equilibrated structures of γ-Li_3_PO_4_ and amorphous Li_3_PO_4_ slabs in an aqueous solution of mixed hydrochloric acid (HCl) and lithium chloride (LiCl). In a “vacancy refilling” process, vacant Li sites were generated by randomly removing lithium ions from the central slab (yellow in Fig. S30), followed by system relaxation over 100 ps repeated over 50 times. The number density of Li^+^ and H^+^ along the normal direction of γ-Li_3_PO_4_ slabs without (left of Fig. 3E) and with vacant Li sites (right of Fig. 3E) clearly indicates H^+^ trapping at the surface layer, which is consistent with amorphous Li_3_PO_4_ slabs (Fig. S31). The preference for Li^+^ transport over H^+^ transport in Li_3_PO_4_ is attributed to the high energy barrier for H^+^ to escape from the strong HBs of H–OPO_3_ with a preferred H–O distance close to 1 Å (Fig. S32). Conversely, Li^+^ can more easily transport among vacant sites (approximately 3 Å) with Li–O distance around 2 Å.

MIP electrolyte with other TMP analogs, including dimethyl methylphosphonate (DMMP) and triethyl phosphate (TEP), also shows a substantial increase in *t*_Li+_ and *D*_Li+_ with the increase in phosphate concentration (Fig. S33A and B), indicating the similar boosting effect of Li^+^ migration kinetics as TMP. Under the same LiMS:phosphate:H_2_O molar ratio as 1.1-*m* LiMS–TMP–H_2_O, the *R*_sei_ of the TiO_2_ anode cycled under 0.9-*m* LiMS–TEP–H_2_O and 1.3-*m* LiMS–DMMP–H_2_O electrolytes remains constant after 50 cycles (Fig. S34A), indicating a stable Li^+^ transfer kinetics on anode surface. In addition, LMO/TiO_2_ battery using 0.9-*m* LiMS–TEP–H_2_O and 1.3-*m* LiMS–DMMP–H_2_O electrolytes exhibits much lower H_2_ evolution rate compared to the 1.1-*m* LiTFSI–H_2_O electrolyte (Fig. S34B), clearly indicating the impeded H^+^ migration on phosphate-based SEI derived from the reduction of TEP and DMMP.

**Fig. 4.**
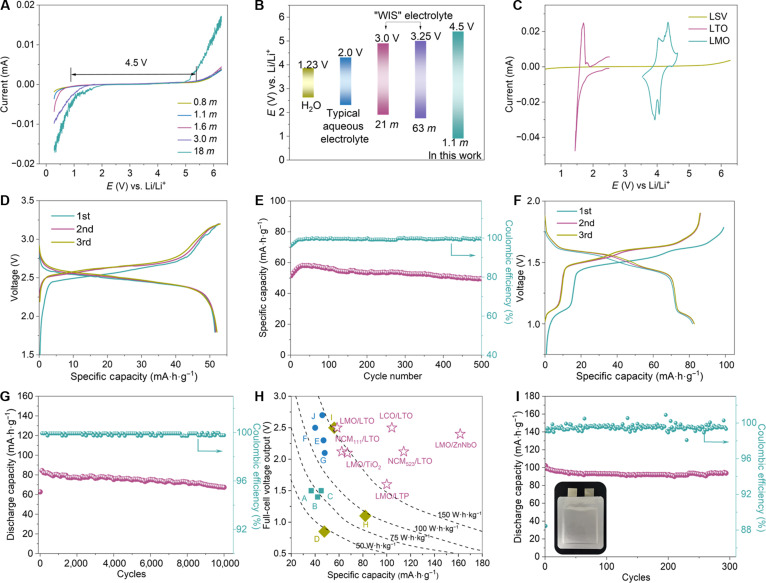
(A) Overall ESW of 0.8-, 1.1-, 1.6-, and 3-*m* LiMS-TMP H_2_O and 18-*m* LiMS–H_2_O electrolyte. (B) Electrolyte stability window (with a scan rate of 5 mV·s^−1^) of 1.1-*m* LiMS–TMP–H_2_O electrolyte in this work, pure H_2_O, and various aqueous electrolytes with different salt concentrations (traditional dilute aqueous electrolyte [106], 21 *m* [9], 28 *m* [52], and 63 *m* [13]). (C) ESW of 1.1-*m* LiMS–TMP–H_2_O electrolyte overlaid with cyclic voltammograms of LMO/LTP couple at 0.2 mV·s^−1^. (D) Voltage profile and (E) cycling performance of LMO/LTO aqueous battery with 1.1-*m* LiMS–TMP–H_2_O electrolyte at 5 C. (F) Voltage profile at 0.5 C and (G) cycling performance at 5 C of LMO/LTP aqueous battery with 1.1-*m* LiMS–TMP–H_2_O electrolyte. (H) Average output voltage of aqueous full cells with specific capacity based on different electrochemical couples under 1 C (A [50], B [51], C [51], D [71], E [9], F [72], G [52], H [73], I [13], and J [23]). Different colors represent different operation times during cycling: orange, below 100 h; blue, 100 to 1,000 h; green, above 1,000 h. (I) Cycling performance of 100-mA·h LMO/LTP pouch battery with 1.1-*m* LiMS–TMP–H_2_O electrolyte under 0.5 C. Inset shows the picture of the pouch cell. Please note that all the capacities in this manuscript are based on the mass of anode materials.

Such Li_3_PO_4_-tailored Li^+^ conduction and hindrance of H^+^ migration at the electrode–electrolyte interface by Li_3_PO_4_ effectively enhance the overpotential of HER and oxygen evolution reactions (OERs) by preventing H_2_O from the bulk electrolyte, thereby extending the cathodic/anodic limits of the electrolyte. As illustrated in Fig. 4A, the overall ESW of various MIP electrolyte expands with increasing TMP concentration, pushing the gas (O_2_ and H_2_) evolution voltages well beyond the electrochemical stability limits of H_2_O. Further examination of Fig. 4A reveals a negative shift in the H_2_ evolution voltage from 1.8 V in an 18-*m* LiMS–H_2_O electrolyte to 0.8 V in a 0.8-*m* electrolyte (Fig. S35A). The cathodic current at 1 V decreases from 5.1 × 10^−3^ mA in the 18-*m* LiMS–H_2_O electrolyte to 7.2 × 10^−5^ mA in the 0.8-*m* electrolyte (Fig. S35B), possibly due to the passivation of the Li_3_PO_4_-based SEI. In addition, the monolithic CEI (Fig. 2F to H) with sluggish H^+^ conductions blocks HO*, O*, and HOO* intermediate on cathode surface from the bulk electrolyte, which contribute to the suppressed OERs as evidenced by the reduced anodic current above 5.0 V. Overall, an ESW of approximately 4.5 V is achieved in 0.8, 1.1, and 1.6-*m* MIP electrolytes (Fig. 4B), with both cathodic (~1 V versus Li/Li^+^) and anodic (~5.2 V versus Li/Li^+^) potentials exceeding those of pure water (cathodic, ~2.63 V versus Li/Li^+^; anodic, ~3.86 V versus Li/Li^+^) and other dilute and concentrated aqueous electrolytes (Fig. 4B). We further investigated the ESW of MIP electrolyte at various scan rates. In line with previous report [37], the scan rate had only a minor effect on the slope of current versus *E* curve and onset of electrolyte decomposition (Fig. S36), highlighting the robustness of the electrolyte’s performance. To fundamentally avoid subjective bias introduced by arbitrarily setting a current density threshold, we innovatively used an objective determination method based on differential calculations in this study. Specifically, instead of relying on a fixed current density threshold value to identify the onset of decomposition, we performed differentiation on the measured linear sweep voltammetry curves and located the zero-crossing points of the differential curves to objectively determine the onset potentials for oxidation and reduction decomposition. The difference between these 2 onset potentials is taken as the measured ESW. As shown in Fig. S37, the ESW measured using this method is approximately 4.5 V, covering a potential range from about 1.0 to 5.5 V (versus Li^+^/Li).

With its low cost and high stability, LTO has been widely utilized as an anode material in Li-ion batteries with organic electrolytes. However, its low Li^+^ intercalation potential greatly limits its application in most aqueous electrolytes, even in high-concentration WIS electrolytes. This challenge can be overcome by MIP electrolyte design. The cyclic voltammogram of LMO/LTO demonstrates that the reversible lithiation/delithiation of LMO and LTO occurs within the cathodic limits of a 1.1-*m* LiMS–TMP–H_2_O electrolyte (Fig. 4C), enabling the full utilization of Li^+^ intercalation/deintercalation sites that seem unattainable in other dilute aqueous solutions. The wide ESW of such an electrolyte can also accommodate the redox potentials of LMO/LTP (Fig. S38) and LMO/TiO_2_ (Fig. S39).

Because of the wide ESW of the 1.1-*m* LiMS–TMP–H_2_O electrolyte, the LMO/LTO aqueous battery can operate with a high average discharge voltage of 2.5 V and a high discharge capacity of 52 mA·h·g^−1^ at 5 C (Fig. 4D). Breaking H_2_O–H_2_O HB networks by the introduction of MS^−^ anion also inhibits H^+^ transport and suppresses the water decomposition process [38], which enable aqueous batteries to deliver an exceptional cycling performance. As shown in Fig. 4E, LMO/LTO aqueous battery demonstrates excellent stability with a capacity retention of over 96% and a high coulombic efficiency of approximately 99.90% after 500 cycles. In contrast, the capacity of the LMO/LTO aqueous battery with a 1.1-*m* LiTFSI–TMP–H_2_O electrolyte rapidly decreases from 85 mA·h·g^−1^ to only 10 mA·h·g^−1^ with a low coulombic efficiency of 47% after 300 cycles (Fig. S40). It is reported that TFSI^−^ anions are unstable toward OH^−^ species generated from H_2_ evolution reaction and a fluorinated SEI is eventually formed during cycling [39], which leads to a notably increasing *R*_sei_ and poor cycling stability due to the impeded Li^+^ migration. We also performed cycling tests of LMO/LTO aqueous batteries using 0.8-, 1.1-, 1.6-, and 3-*m* LiMS–TMP–H_2_O and 18-*m* LiMS–H_2_O electrolytes (Fig. S41). Notably, the 1.1-*m* electrolyte demonstrated superior cyclability under 3 C compared to all other LiMS–TMP–H_2_O electrolytes, experimentally indicating the rationality in the selection of 1.1-*m* LiMS concentration. In sharp contrast, LMO/LTO aqueous batteries using 18-*m* LiMS–H_2_O electrolyte quickly decayed within 180 cycles, further suggesting the necessity of TMP in retaining excellent cycling lifespan. Differential electrochemical mass spectrometry, an in situ analytical method, was used to track gas evolution in cycled cells as a function of TMP concentration. Figure S42 displays the differential electrochemical mass spectrometry collected from LMO/LTO full cells during the 151st charging process after 150 cycles at 5 C. Consistent with expectations, the TMP-free electrolyte (18-*m* LiMS–H_2_O) exhibited predominant water splitting during charging above 3.2 V, yielding high H_2_ and O_2_ evolution rates of 63 and 17 nmol·min^−1^ (Fig. S42A). However, raising the TMP concentration effectively inhibited this process, with decomposition at the cathode surface being more curbed than at the anode. Specifically, O_2_ almost completely disappears at 3.0 *m* (Fig. S42B), while small amount of H_2_ still generates at the 1.6-*m* LiMS–TMP–H_2_O electrolyte (Fig. S42C). The distinction between H_2_ and O_2_ evolution rate aligns with the well-established understanding that the kinetics of H_2_ evolution are more favorable than those of O_2_ evolution [40], and the root cause for the gradual capacity decay in 1.6- and 3-*m* LiMS–TMP–H_2_O electrolytes (Fig. S41) comes from the inevitable parasitic hydrogen evolution flux on anode surface. At 1.1 *m*, there is no detectable H_2_ and O_2_ evolution during the 151st charging process from LMO/LTO cells (Fig. S42D), indicating the fully suppression of H_2_O decomposition during cycling and ensuring a stable capacity retention after 500 cycles (Fig. 4E).

The LMO/LTP and LMO/TiO_2_ aqueous batteries with a 1.1-*m* LiMS–TMP–H_2_O electrolyte also deliver high output voltages of 1.6 V (Fig. 4F) and 2.1 V (Fig. S43), respectively. By adding 20 cycles of low-rate (0.5 C) charge–discharge before the cycling test, the LMO/LTP aqueous battery can be stably operated for up to 10,000 cycles at 5 C with a capacity retention of 77% (Fig. 4G). The cycling life at 0.5 and 1 C of such a battery with a 1.1-*m* LiMS–TMP–H_2_O electrolyte is also verified by third-party testing (Fig. S44). The charge–discharge platforms of the LMO/LTP full cell for the 100th, 200th, and 300th cycles remain consistent, further indicating the excellent cycling stability of the system (Fig. S45). We also utilized the LMO/LTP electrode pair to evaluate the rate performance of the 1.1-*m* LiMS–TMP–H_2_O electrolyte. As illustrated in Fig. S46, the specific capacities of the battery under various current densities were approximately 84 mA·h·g^−1^ at 0.5 C, 82 mA·h·g^−1^ at 1 C, 79 mA·h·g^−1^ at 2 C, 72 mA·h·g^−1^ at 5 C, and 56 mA·h·g^−1^ at 10 C. These data clearly indicate that the battery maintains considerable capacity output across a broad range of current densities from 0.5 to 10 C. Furthermore, after undergoing high-rate testing at 10 C, the capacity retention returned to 100% upon reverting to 0.5 C, fully demonstrating its excellent rate performance. We further compared the performance of the LMO/LTP electrode pair in our work with studies using other aqueous electrolytes, including WIS electrolytes, conventional Li_2_SO_4_ dilute electrolyte, and saturated Li_2_SO_4_ electrolytes (Fig. S47). The comparison revealed that at lower charge–discharge current densities (1 and 2 C), the performance of our battery was comparable to that of these alternative aqueous electrolytes. However, at higher current densities (5 and 10 C), our battery exhibited substantially higher specific capacities [41–45], further evidencing the enhanced Li^+^ transfer kinetics in the 1.1-*m* LiMS–TMP–H_2_O electrolyte. LMO/LTP aqueous battery maintains excellent cycling stability and high coulombic efficiency at 40 and 50 °C (Fig. S48). However, at 60 °C, a sudden decline in cycling stability and coulombic efficiency is observed. This is attributed to the decomposition of TMP molecules at elevated temperatures, which leads to the release of phosphorus-containing gases that absorb energy and contributes to performance loss in cycling stability [46–49].

It is widely recognized that increasing the output voltage from aqueous batteries typically leads to a significant compromise in cycling stability, even when using WIS electrolytes [50,51]. Apparently, MIP electrolyte enables us to overcome such voltage/cycling trade-offs and achieve high values in both aspects in LMO/LTO, LMO/LTP, and LMO/TiO_2_ battery systems (Fig. 4H). Moreover, the electrolyte demonstrates exceptional compatibility with a diverse range of battery systems, extending but not limited to LMO/ZnNbO (Fig. S49), LCO/LTO (Fig. S50), NCM_111_/LTO (Fig. S51), and NCM_523_/LTO configurations (Fig. S52), with the highest output voltage of 2.5 V (LCO/LTO) and a specific capacity of 161 mA·h·g^−1^ (LMO/ZnNbO) at 1 C (Fig. 4H). The corresponding cycling performance of LMO/TiO_2_, LMO/ZnNbO, LCO/LTO, NCM_523_/LTO, and NCM_111_/LTO full cells are presented in Fig. S53. TiO_2_ (Fig. S53A) and ZnNbO (Fig. S53B) anodes exhibit slightly higher specific capacities and superior cycling stability in comparison with previous reports [52,53]. For LCO (Fig. S53C) whose application is traditionally limited by side reactions in aqueous systems, our electrolyte system enables it to achieve a specific capacity similar to prior reports while improving cycling stability [54,55]. However, NCM electrodes (Fig. S53D and E) show reduced cycling retention, likely due to the dissolution of Ni ions in the aqueous electrolyte, a well-known issue for ternary cathodes in aqueous systems [56]. Clearly, LMO/LTO (ceiling discharge voltage of 2.5 V) and LMO/LTP (ceiling discharge voltage of 1.5 V) show much better cycling performance (Fig. 4E and G) than LMO/TiO_2_, LMO/ZnNbO, LCO/LTO, NCM_111_/LTO, and NCM_523_/LTO (Fig. S53) with 1.1-*m* LiMS–TMP–H_2_O electrolyte. As a result, LMO/LTO and LMO/LTP are recommended electrode couples for the practical application of LiMS–TMP–H_2_O electrolyte in grid-scale energy storage systems that are sensitive in lifespan. It should also be noted that the primary contribution of this work lies in proposing an electrolyte design strategy that expands the ESW, thereby offering a potential solution for high-voltage electrode pairs and verifying its basic compatibility across different material systems. Therefore, the results stated here are strictly based on the fundamental electrochemical performance data we presented when applying the electrolyte with various electrode pairs. We do not intend to claim, nor have we claimed, that this electrolyte can universally address all specific degradation challenges faced by high-voltage cathode materials.

We also fabricated a 100-mA·h LMO/LTP pouch battery with a 1.1-*m* LiMS–TMP–H_2_O electrolyte. The full cell (cathode [35 mg·m^−2^] and anode [20 mg·m^−2^] with a positive/negative electrode capacity ratio [P/N] of 1.73) can sustain over 300 cycles at 0.5 C with a capacity retention of 93% and a coulombic efficiency of 99.97% (Fig. 4I). Our pouch cell achieves a high energy density comparable to state-of-the-art systems (Fig. S54), whether evaluated by real gravimetric energy density (40 and 43.4 W·h·kg^−1^ excluding the battery casing) [53,57,58] or electrode material mass (165 W·h·kg^−1^) [59–70]. In addition, our pouch cell demonstrates outstanding cycling stability, maintaining excellent performance over 300 cycles (Table S4). It is important to note that aqueous batteries and organic electrolyte-based Li-ion batteries differ fundamentally in their application focus and technological pathways. Traditional high-energy-density Li-ion batteries (200 to 250 W·h·kg^−1^) are primarily designed for consumer electronics and electric vehicles, where energy-to-weight ratio is extremely sensitive. In contrast, this aqueous battery system is developed with priority on intrinsic safety, environmental friendliness, low cost, and long cycle life. It targets applications such as large-scale energy storage and low-speed electric vehicles where absolute weight and volume constraints are relatively relaxed, but stringent requirements exist for safety, cost, and cycling durability.

**Fig. 5.**
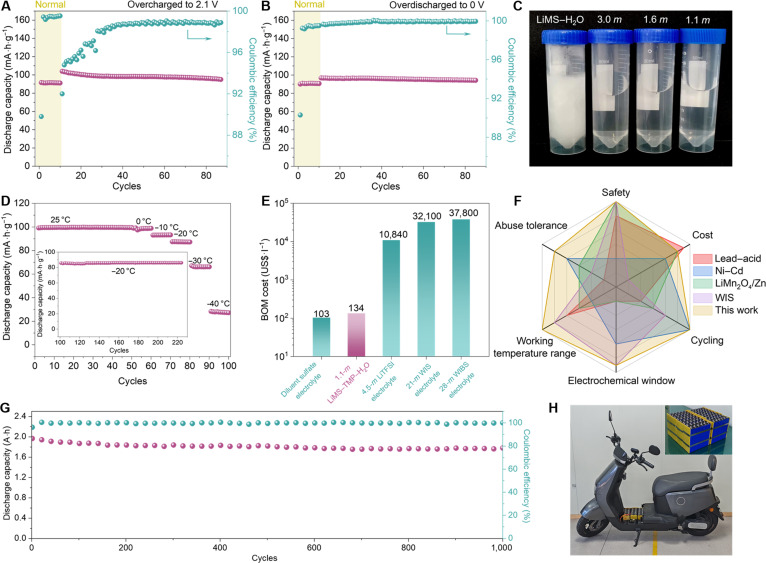
(A) Overcharged and (B) overdischarged performance of LMO/LTP battery with 1.1-*m* LiMS–TMP–H_2_O electrolyte. (C) Optical photographs of LiMS–H_2_O electrolyte and 1.1-, 1.6-, and 3.0-*m* LiMS–TMP–H_2_O electrolytes at −30 °C. (D) Discharge capacity (at 0.2 C) of LMO/LTP battery with 1.1-*m* LiMS–TMP–H_2_O electrolyte under various working temperature (−40 to 25 °C). Inset shows the cycling performance of this battery at 0.2 C under −20 °C. (E) BOM cost comparison of 1.1-*m* LiMS–TMP–H_2_O electrolyte with dilute sulfate electrolyte (2 M ZnSO_4_/1 M Li_2_SO_4_) in aqueous LMO/Zn batteries, 4.5-*m* LiTFSI electrolyte [LiTFSI–KOH–CO(NH_2_)_2_–H_2_O], 21-*m* WIS electrolyte, and 28-*m* WIBS electrolyte. (F) Comparison of 1.1-*m* LiMS–TMP–H_2_O electrolyte-based batteries with other state-of-art commercial or laboratory-scale aqueous battery technologies, such as lead–acid, Ni–Cd, LMO/Zn, and “WIS” batteries. (G) Cycling performance of 2-A·h LMO/LTP pouch battery with 1.1-*m* LiMS–TMP–H_2_O electrolyte under 1 C. (H) Practical demonstration of the assembled commercial LMO/LTP aqueous battery with 1.1-*m* LiMS–TMP–H_2_O electrolyte in electric bike. The energy consumption metric of the battery is 60 V and 15 A·h.

In traditional aqueous batteries, severe water consumption, cell swelling, and significant capacity degradation occur during overcharging or deep discharging due to increased H_2_/O_2_ evolution under these abusive conditions [71,72]. Surprisingly, this issue is mitigated in the aqueous battery with MIP electrolyte. When overcharged to 2.5 V in each cycle at 0.5 C (Fig. S55), the LMO/LTP battery with 1.1-*m* LiMS–TMP–H_2_O electrolyte shows over 95% capacity retention with 98.26% coulombic efficiency after 85 cycles (Fig. 5A). Similar cycling performance is observed in the cell discharged to 0 V at 0.5 C (Fig. 5B and Fig. S56), indicating the safety advantage of such an electrolyte during practical operations. The excellent deep discharge and overcharge performance of such a battery are further confirmed by third-party institute testing (Fig. S57). To more intuitively demonstrate the intrinsic safety of the battery, we also supplemented nail penetration and short-circuit tests of LMO/LTP battery pack with 1.1-*m* LiMS–TMP–H_2_O electrolyte. The video (Movie S2) clearly documents that during these extreme abuse conditions, the battery showed no deformation, smoking, ignition, or explosion, and no thermal runaway occurred. This directly confirms its excellent safety performance.

The effective disruption of H_2_O–H_2_O HB networks by MS^−^ anions greatly increases the “tetrahedral entropy” of H_2_O molecules, which lowers the freezing point of water [38] and enables much higher ionic conductivity over −60 to +20 °C (Fig. S58) than the electrolytes with other lithium salts (LiTFSI, LiClO_4_, and LiBF_4_). Compared to the LiMS–H_2_O electrolyte that solidifies at −40 °C, the 3.0-, 1.6-, and 1.1-*m* LiMS–TMP–H_2_O electrolytes remain liquid (Fig. 5C). Because of the antifreezing property of such electrolyte, the LMO/LTP battery with 1.1-*m* LiMS–TMP–H_2_O electrolyte can operate at 0.2 C over a wide temperature range (−40 to 25 °C; Fig. 5D; with corresponding charge–discharge curves shown in Fig. S59). This battery also demonstrates nearly 100% capacity retention after 100 cycles at −20 °C (inset of Fig. 5D).

LiMS is a low-cost salt derived from the neutralization of LiOH and CH_3_SO_3_H in distilled water [73]. TMP is also an inexpensive compound widely used in electroplating and textile manufacturing as a flame retardant [37,38]. As a result, the bill of materials (BOM) cost of 1.1-*m* LiMS–TMP–H_2_O electrolyte (Fig. 5E) is only 0.41% of a 21-*m* WIS electrolyte [9], 0.35% of a 28-*m* WIBS electrolyte [30], and 1.2% of a recently reported 4.5-*m* LiTFSI–KOH–CO(NH_2_)_2_–H_2_O electrolyte [14] and is close to that of a sulfate-based aqueous electrolyte (2 M ZnSO_4_/1 M Li_2_SO_4_) used in aqueous LMO/Zn batteries [39]. Considering other factors such as safety, cycling performance, ESW, working temperature range, and abuse tolerance, it is anticipated that such an electrolyte design is superior to state-of-the-art commercial or laboratory-scale aqueous technologies, such as lead–acid, Ni–Cd, LMO/Zn, and “WIS” batteries (Fig. 5F). The low BOM cost of such MIP electrolyte also encourages us to fabricate scaled-up ampere-hour class aqueous batteries. As shown in Fig. 5G, 2-A·h LMO/LTP aqueous battery based on MIP electrolyte exhibits >98% capacity retention over 1,000 cycles while maintaining an average coulombic efficiency of ∼99%. For further practical application, we also assembled a 60-V, 15-A·h commercial LMO/LTP aqueous battery using MIP electrolyte (Fig. 5H), which drives an electric bike to run smoothly for a demonstration distance of 70 km (Fig. S60) and underscores its significant potential in replacing environmentally toxic lead–acid batteries in low-speed vehicles.

## Conclusion

**Fig. 6.**
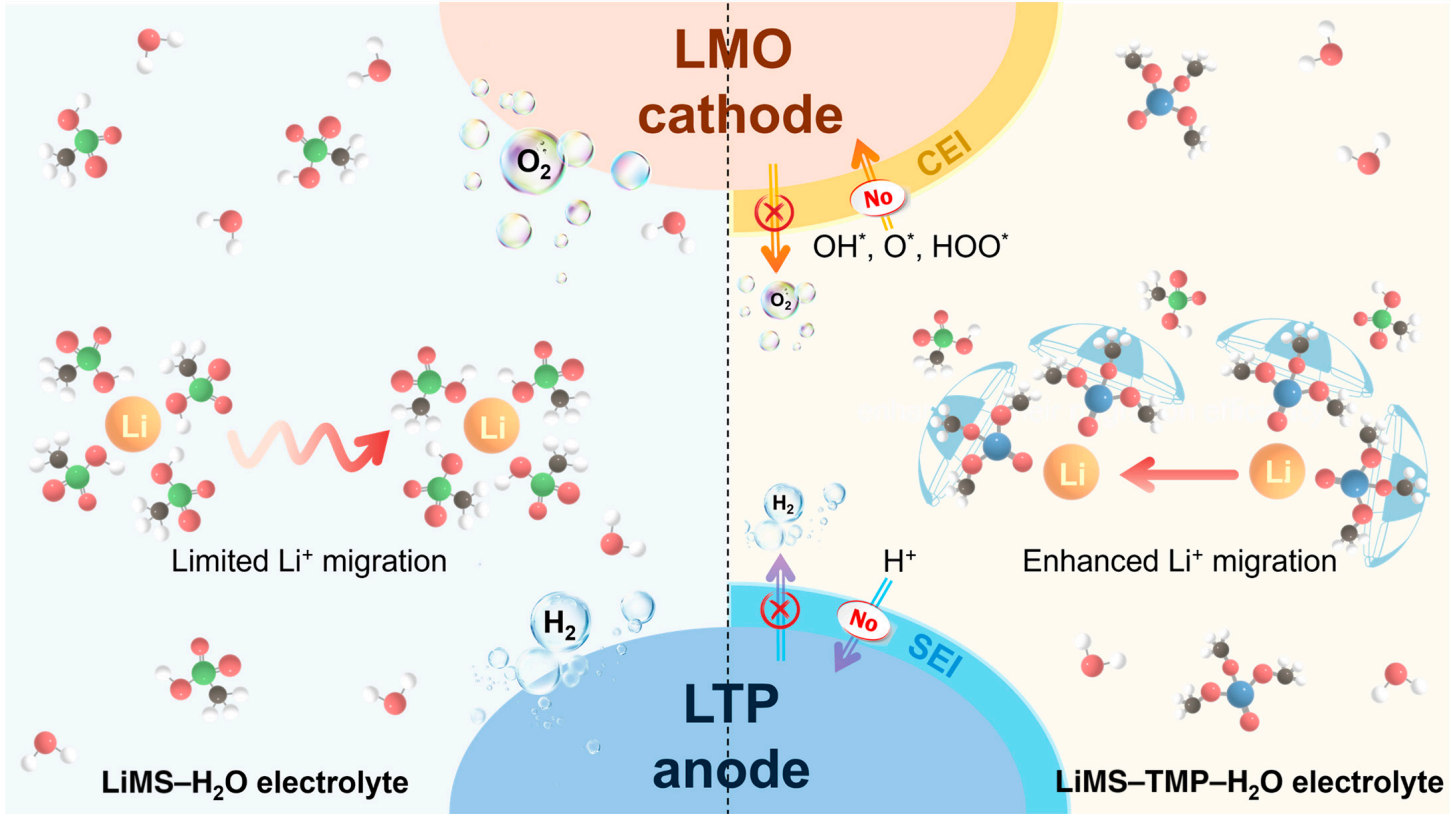
Schematic diagram of simultaneously boosted liquid and interfacial Li^+^ conduction in MIP electrolyte.

In summary, we have identified simultaneously boosted liquid and interfacial Li^+^ conduction in an aqueous electrolyte due to the synergic superionic pairing between TMP and MS^−^ anions in MIP electrolyte (Fig. 6). On one hand, the phosphate group carries 3 nonpolar alkyl groups (–CH_3_) while exposing an oxygen site to coordinate with Li^+^. Such amphipathic structure exhibits a bulky nonpolar region and a single coordination site, functioning like an umbrella that shields Li^+^ from extensive interactions with MS^−^ anions in the electrolyte and thereby enhancing their migration efficiency. In another hand, strongly polarized –SO_3_^−^ from MS^−^ form strong ion–dipole interactions with H^+^ in H_2_O molecules. It leads to a smaller and more dynamic solvation shell, faster solvation exchange, and reduced competition from proton conduction—further promoting Li^+^ transport kinetics. Meanwhile, we also observed a selective diffusion of Li^+^ over H^+^ in electrode–electrolyte interfaces, which is due to the trapping of proton by phosphate groups and facilitated “vacancy refilling” Li^+^ diffusion processes in Li_3_PO_4_-based CEI and SEI. Such Li^+^-conducting and H^+^-impeding interfacial chemistry blocks H^+^ transfer to the anode and oxygen-based intermediates (HO*, O*, and HOO*) to the cathode, simultaneously suppressing interfacial HER and OER (Fig. 6) and enabling a series of aqueous electrolytes with a wide ESW (up to 4.5 V) and an exceptional lifespan (up to 10,000 cycles). More importantly, this MIP electrolyte design is applicable in a wide range of battery systems, including LMO/LTP, LMO/TiO_2_, LMO/LTO, LMO/ZnNbO, LCO/LTO, NCM_111_/LTO, and NCM_523_/LTO configurations, with a high output voltage (up to 2.5 V) and remarkable temperature adaptability. Attributing to its low BOM cost that is only 0.41% of the widely used 21-*m* WIS electrolyte for high-voltage aqueous batteries, such MIP electrolyte also provides wide operating temperature range (−40 to 25 °C) and superior cycling performance (>88% capacity retention after 1,000 cycles) in 2-A·h class pouch battery. The assembled 60-V, 15-A·h commercial LMO/LTP aqueous battery could drive an electric bike smoothly for a maximum distance of 70 km, demonstrating a breakthrough in replacing toxic lead–acid batteries in low-speed vehicles.

## Methods

### Materials and synthesis

LiMS–TMP–H_2_O electrolyte (1.1 *m*) was prepared by a mixture of LiMS (LiCH_3_SO_3_), deionized water, and TMP (>99%; Sigma-Aldrich). To synthesize LiCH_3_SO_3_, 14.45 g of methanesulfonate acid solution (70 wt % in water; Sigma-Aldrich) was dissolved in 50 ml of water and magnetic stirred at 80 °C when 2.4 g of lithium hydroxide (LiOH; reagent grade, 98%; Sigma-Aldrich) was added gradually. The solution was stirred at 80 °C for 2 h until all water evaporated. Finally, the product was collected and ground, followed by drying at 100 °C under vacuum overnight.

TiO_2_ for battery test was synthesized as following method: 4 g of anatase TiO_2_ (>99%; Sigma-Aldrich), 0.2 g of iron(III) oxide (Fe_2_O_3_; powder < 5 μm; Sigma-Aldrich), and 2 g of starch (from potato; Sigma-Aldrich) were mixed and ground for 15 min, followed by heated at 900 °C with a heating rate of 5 °C·min^−1^ in argon for 4 h. The product was then stirred in 4 M HCl solution at 60 °C for 2 h and rinsed 3 times with deionized water to eliminate Fe ions. Finally, the product was dried at 80 °C overnight. LTP was prepared by sol–gel process and carbothermal reaction. Lithium dihydrogen phosphate (LiH_2_PO_4_; 24.6 kg, >99%; Aladdin), ammonium dihydrogen phosphate (NH_4_H_2_PO_4_; 51.5 kg, >99%; Aladdin), TiO_2_ (36.8 kg, >99%; Aladdin), and β-cyclodextrin (7.12 kg, >99%; Aladdin) were placed in a ball mill along with 180 kg of deionized water. The mixture was milled for 4 h to obtain a dispersed mixture with a solid content of 40%. Subsequently, the dispersed mixture was transferred to a sand mill using a diaphragm pump for ultrafine grinding. The high-speed sand milling process lasted for 4 h, resulting in a slurry with particle sizes controlled between 500 and 800 nm. The obtained slurry was then subjected to spray granulation in a spray granulator, maintaining an inlet temperature of 200 to 225 °C and an outlet temperature of 100 °C. This process yielded 108 kg of precursor material. The precursor material was then mixed uniformly with 10 kg of glucose (>99%; Aladdin) for 2.5 h. The mixture was then sintered at 800 °C under an argon atmosphere for 12 h at a rate of 10 °C·min^−1^. Finally, the product was naturally cooled to room temperature, ground, and collected. ZnNbO was synthesized by a solid-state reaction method. Niobium(V) oxide (Nb_2_O_5_; 99.9%; Sigma-Aldrich) and zinc oxide (ZnO; >99%; Sigma-Aldrich) were mixed stoichiometrically and ball-milled at 800 rpm for 6 h, followed by calcinated in air at 1,200 °C for 6 h. The product was naturally cooled to room temperature, collected, and ground to obtain ZnNbO. LMO, NCM_111_, and NCM_523_ were purchased from MTI Corporation.

### Electrochemical measurements

CR2025 coin cells were assembled with cathode discs (diameter, 14 mm), absorbent glass mat separators (diameter,16 mm), and anode discs (diameter, 12 mm) as anode, and 125 μl of electrolyte was added. The composition of the electrodes is 70 wt % electrode material, 20 wt % acetylene black (AB; MTI Corporation), and 10 wt % polyvinylidene fluoride. The P/N ratio for the coin cell is 1.16. Pouch cells (100 mA·h) were assembled with one piece of LMO (>99%; MTI Corporation, single-side coating) cathode piece (66 mm × 96 mm), absorbent glass mat (66 mm × 96 mm × 0.2 mm), one piece of LTP anode (single-side coating, 56 mm × 86 mm), with 10 to 15 g of electrolytes added per ampere-hour. The composition of cathode piece is 83.5 wt % LMO, 13 wt % AB, 2 wt % carboxymethyl cellulose (MTI Corporation), and 1.5 wt % styrene-butadiene rubber (MTI Corporation) on an aluminum foil as current collector, and the mass loading of cathode is about 35 mg·cm^−2^. The composition of anode piece is 90 wt % LTP, 6 wt % AB, 2 wt % carboxymethyl cellulose, and 1.5 wt % styrene-butadiene rubber on an aluminum foil as current collector, and the mass loading of anode is 20 mg·cm^−2^. The P/N value of the prepared cathode and anode is 1.73. The prepared cathode pieces, anode pieces, and separators were neatly stacked in the order of cathode–separator–anode and then fixed with adhesive tape. The cathode and anode leads were drawn out using an ultrasonic welding machine, and the welding points were covered with high-temperature tape to form a standard cell. The cell was then placed in a suitable aluminum–plastic film, and the premixed electrolyte was injected into the cell under vacuum at a mass of 10 to 15 g per ampere-hour. The pressure was maintained for 10 min, followed by heat sealing in vacuum to complete the battery fabrication process. The fabricated pouch cells were clamped with reinforcement plates for charge and discharge testing.

To assemble 2-A·h pouch cell, 8 pieces of LMO (>99%; MTI Corporation) cathode (185 mg·cm^−2^, 140 mm × 67 mm, 12-μm-thick aluminum foil as current collector) and 7 pieces of LTP anode (160 mg·cm^−2^, 137 mm × 63 mm, 12-μm-thick aluminum foil as current collector) were stacked with 9-μm-thick polypropylene separators (143 mm × 70 mm). The following fabrication process of 2-A·h pouch cell is the same as 100-mA·h pouch cell. To assemble an industrial-level 60-V, 15-A·h commercial LMO/LTP aqueous battery, 59 pieces of LMO (>99%; MTI Corporation) cathode (17.5 mg·cm^−2^, 140 mm × 67 mm, 12-μm-thick aluminum foil as current collector) and 58 pieces of LTP anode (16.5 mg·cm^−2^, 137 mm × 63 mm, 12-μm-thick aluminum foil as current collector) were firstly stacked with 9-μm-thick polypropylene separators (143 mm × 70 mm) to form a single cell. After adding 140 g of 1.1-*m* LiMS–TMP–H_2_O electrolyte, such single cell was sealed and aged 24 h before charge–discharge tests. The battery pack to drive electric bike is composed of 40 single cells connected in series. The electric bike test is conducted at 25 °C with a riding speed of approximately 25 km·h^−1^. The total load was approximately 137.7 kg, including the electric bike’s own weight (~77.7 kg, battery pack included) and the rider’s weight (~60 kg).

The ESW was determined by linear sweep voltammetry at a scan rate of 10 mV·s^−1^ using a BioLogic VMP3 workstation with a 3-electrode configuration. An Ag/AgCl electrode served as the reference, while a 316 stainless steel grid (200 mesh) functioned as both the working and counter electrodes. Prior to testing, all electrodes were ultrasonically cleaned in high-purity alcohol, rinsed 3 times with high-purity water, and dried. Measurements were carried out in a Swagelok T-type cell with a working electrode area of approximately 1 cm^2^.

Cyclic voltammetry was conducted on the same workstation at a scan rate of 0.1 mV·s^−1^. For cathode testing, the 3-electrode setup comprised active material (∼2 mg) as the working electrode, a carbon rod as the counter electrode, and Ag/AgCl as the reference electrode. For anode testing, the working electrode consisted of active material (∼1.5 mg), with a 2-mm platinum wire as the counter electrode and Ag/AgCl as the reference. Galvanostatic charge–discharge cycling was performed using a Neware battery tester under constant current mode at various current densities. The specific current densities corresponding to 1 C were 68 mA·g^−1^ for LTO, 100 mA·g^−1^ for LTP, and 200 mA·g^−1^ for ZnNbO-based systems.

The Li^+^/H^+^ conductivity of Li_3_PO_4_ and LiF pellets was evaluated by EIS using an Admiral Instruments workstation, with a frequency range from 100 kHz to 100 mHz at 25 °C. Specifically, 500 and 100 mg of commercial γ-Li_3_PO_4_ (or LiF) powders were first hydraulically pressed into pellets with a diameter of 10 mm under applied loads of 4 and 8 tons, respectively, to fabricate millimeter-scale (around 2.1 mm in thickness) and micrometer-scale (350 to 980 μm in thickness) pellets for conductivity measurements. Then, the pellet was attached with wetted glass fiber (wetted by 1 M Li_2_SO_4_ solution) and then sandwiched between 2 stainless steel spacers to measure the EIS [74]. The Li^+^ conductivity (*σ*_Li+_) was calculated from *σ*_Li+_ = *l*/*RS*, where *R* is the total impedance (from EIS result), *l* is the sample thickness, and *S* is the area (1.13 cm^2^) [75]. To measure the H^+^ conductivity of Li_3_PO_4_ and LiF pellets, the hydraulic pressed pellet was firstly attached with wetted glass fiber (wetted by 1 M Li_2_SO_4_ solution) and then sandwiched between 2 stainless steel spacers in a sealed chamber with wet H_2_ (humidified 7% H_2_ in Ar) atmosphere [76]. The H^+^ conductivity (*𝜎*_H+_) is obtained on the basis of the equation *𝜎*_H+_ = *𝜎*_m_ − *𝜎*_Li+_, where *𝜎*_m_ is the measured ionic conductivity of Li_3_PO_4_ (or LiF) pellets in H_2_ atmosphere and *𝜎*_Li+_ is Li^+^ conductivity measured in air.

### Material characterization

FT-IR spectra were acquired using a Brüker Vertex 70 spectrometer over the wave number range of 400 to 4,000 cm^−1^. Raman spectroscopy was conducted on a Horiba HR800 spectrometer in a backscattering configuration. This system, with a spectral resolution of 0.3 cm^−1^, used a continuous-wave 633-nm HeNe laser (∼1-mW maximum output) for both sample excitation and spectral collection. XPS measurements were performed on a Thermo Fisher Scientific ESCALAB 250 microprobe. Prior to analysis, sample surfaces were cleaned by Ar^+^ sputtering under an argon partial pressure below 10^−8^ torr. Spectra were collected using a 200-μm x-ray beam (1,486.6 eV, 49.3 W), with binding energies referenced to the adventitious C 1s peak at 284.8 eV. All measurements were carried out under an ultrahigh vacuum (chamber pressure maintained at 2.0 nPa), and a wide survey scan (0 to 1,200 eV) was obtained for each sample to determine surface elemental composition. TEM was carried out using a Zeiss Libra 200MC instrument. Thermogravimetric analysis was performed using the TA Instruments Q500 in air from room temperature to 800 °C and from room temperature to 900 °C, respectively. An Agilent 8890 gas chromatography system, equipped with a CP-Molsieve 5-Å PT column and a thermal conductivity detector, was used to quantify H_2_ evolution. The gas generated from the operating battery was channeled directly into the system’s automatic sampling loop. High-purity argon was used as the carrier gas with a sampling interval of 15 min [77].

Water activity coefficient was obtained from a vapor–pressure measurement device [78]. Samples were put in a plastic container connected to a vacuum pump with a solution/free space ratio of 1:1. The container was degassed and flushed several times with Ar. After further purging with argon, the container was sealed, and the total pressure was measured with the time when the vapor is equilibrated with liquid phase. Before recording, a thermocouple was placed in the liquid to guarantee the temperature (22 °C). The measurement assumed that the vapor originated from pure water, yielding a saturated vapor pressure of 2.69 kPa. This value was then applied in Raoult’s law to calculate the corresponding water activity coefficient [79].

### Computational methodological details

#### MD simulations of electrolyte systems

We applied MD simulations to investigate the structure and interactions of ion aggregates in lithium electrolytes with methanesulfonic acid lithium [Li(CH_3_SO_3_), LiMS], H_2_O, and TMP in different ratios. All MD simulations were carried out using the general AMBER force field [80]. First, geometry optimizations for MS and TMP were carried out at the HF/6-31G* level using Gaussian g09 [81] calculations. With Gaussian log file, we used antechamber to perform the restricted electrostatic potential fitting and produced the AMBER PREP files for MS and TMP, respectively. With the MS and TMP parameters ready, we constructed a 60 × 60 × 60 Å^3^ box that contains LiMS, transferable intermolecular potential 3P model H_2_O, and TMP molecules in molar ratio as LiMS:H_2_O:TMP = 1:3:*X*, *X* = 0, 2, 4, and 6, respectively. The MS, lithium ion, TMP, and H_2_O were randomly placed in the box using PACKMOL [82] and further processed using LEaP in AMBER18 package [83]. Each system is simulated with 3 copies. The details of MD simulations are shown in Table S5.

#### Simulation setups for electrolyte systems

Each system went through energy minimization, 125-ps equilibration under isothermal–isovolumetric (NVT) ensemble with position restraint on solute component and a time step of 1 fs. The system continued to relax without restraint under isothermal–isobaric (NPT) ensemble using the AMBER18 package [83]. The NPT ensemble was used for further equilibration and MD production under 298 K, 1 bar, Langevin dynamics thermostat, and a time step of 2 fs. The simulations time in NPT ensemble are listed in Table S5 as 2,000 ns for each simulation. The particle mesh Ewald technique [84] was applied to calculate long-range electrostatic interactions. The short-range van der Waals and electrostatics were cut off at 12.0 Å with switch at 10.0 Å.

#### MD simulations of Li and proton diffusion in Li_3_PO_4_ interface systems

The γ-Li_3_PO_4_ crystal structure in the *Pnma* space group (no. 62) is taken from Materials Project database [85,86] with unit cell parameters *a* = 4.89 Å, *b* = 6.07 Å, and *c* = 10.42 Å. The γ-Li_3_PO_4_ slab contains 6 × 6 × 2 units (36.426 × 29.358 × 20.832 Å^3^) with the (100) plane chosen as the solid–liquid interface. A transferable intermolecular potential 3P water box containing 3 M HCl and 3 M LiCl was built and put above the Li_3_PO_4_ slab. The whole Li_3_PO_4_–solution system contains 5,229 atoms and 903 H_2_O molecules in a periodic box of 36.426 × 29.358 × 57 Å^3^. Bonded and nonbonded parameters used for PO_4_^3−^ are displayed in Table S6. The “Zundel cation” kind H^+^ parameter [87] was used, with the 12–6–4 parameter, *R*_min_/2, modified to 0.841 Å (in Table S6). As studied in the paper of Li et al. [87], 3 H^+^ types (the zundel cation, eigen cation, and H_3_O^+^) are parameterized. In the beginning of our interfacial systems, we carefully tested and evaluated the 3 H^+^ models (all are parameterized as single spheres) and eventually derived the H^+^ parameter for our study. First, the H_3_O^+^-type H^+^ model (with *R*_min_/2 parameter as 1.72 Å in the paper of Li et al.) describes a large ion–oxygen distance (IOD) of ~2.5 Å. In real-crystal Li_3_PO_4_ layer, H^+^ is more easily to diffuse instead of bulky H_3_O^+^, which needs further dissociation of H^+^ from H_3_O^+^. Thus, to describe the proton diffusion in slab instead of solution, the zundel cation-type H^+^ or eigen cation-type H^+^ with smaller *R*_min_/2 parameters (0.925 and 0.841 Å, respectively) is better. However, our test simulation for eigen cation-type H^+^ is very unstable. In addition, the origin zundel cation-type H^+^ parameter generates an IOD distance of 1.3 Å. Eventually, the zundel cation-type H^+^ parameters with the *R*_min_/2 parameter modified to 1.841 Å are used in Table S6, which generate a reasonable IOD distance of ~1.25 Å (similar to the paper of Li et al. [87]).

The amorphous Li_3_PO_4_ was generated by rising the temperature to 2,000 to 2,500 K in MD simulation to accelerate the melting of crystal structure. After amorphous structure was formed, the system continues to be relaxed under 298 K. Each system is simulated with 3 copies. The message passing interface of AMBER18 package [83] is used to perform the minimization and MD simulations. Systems are relaxed under semi-isotropic pressure coupling (NPγT) ensemble (298 K, 1 bar) using Berendsen barostat and a time step of 1 fs. Same setups are used for long-range electrostatic interactions and short-range van der Waals and electrostatics as they are in the electrolyte systems. Similar setups to those in the electrolyte systems are used for energy minimization, followed by 125-ps equilibration under NVT ensemble with position restraint on the Li_3_PO_4_ slab. The simulations time in NPγT ensemble are listed in Table S5 as 200 ns for each simulation.

With the well-equilibrated systems, we further study the preference of Li^+^ and H^+^ diffusion in Li_3_PO_4_ slab. We generate vacant Li sites by removing random Li ions from the middle slab (yellow in Fig. 4A and B) and relax the system in 100-ps MD simulations; we call this process a “vacancy refilling” process. In general, 10 vacant Li sites are maintained in the beginning of a “vacancy refilling” process for γ-Li_3_PO_4_ and roughly 10 to 20 vacant Li sites for amorphous slab. The “vacancy refilling” process was repeated over 10 times in each simulation of 5 parallel copies. A summary of MD simulations is provided in Table S5.

#### PDOS calculations

We performed a series of hybrid DFT calculations to obtain the PDOS using the Quickstep module of the CP2K program suite [88]. To prepare structures for the PDOS calculations, LiMS–H_2_O and 1.1-*m* systems with over 1,000 atoms were equilibrized and relaxed in MD simulations for over 200 ns, which display similar coordination distribution with their corresponding large size systems. We extracted 10 configurations in 20-ns interval from the last 200 ns of each simulation; a total of 30 different configurations of 600 ns were used to generate the PDOS for each system. In Quickstep, a dual basis of localized Gaussians and plane waves is used to represent the Kohn–Sham equation and electronic density [89]. We used the molecularly optimized (MOLOPT) basis set with double-ζ plus polarization (DZVP-MOLOPT-GTH), as well as a planewave cutoff of 400 Ry and a REL_CUTOFF of 60 Ry. Hartree–Fock exchange (HFX) was applied with its efficient extension (the auxiliary density matrix method) [90], in which the contracted basis set with polarization function, cpFIT3, was applied as auxiliary basis set. The truncated HSE06 hybrid functional was adopted for the exchange correlation energy functional, where part of the exchange functional is replaced by HFX. Truncated Coulomb potential with cutoff radius less than half the cell was applied in the HFX calculation. The orbital transformation method is used in the self-consistent field wave function optimization cycle. The PDOSs of different components were averaged over 30 different configurations of 600 ns to obtain a representative ensemble average.

#### QM calculations

We performed QM calculations to study the reduction potential of TMP with and without Li using Gaussian g09 [81]. The initial TMP or TMP–Li structures were taken from MD simulation. The half-cell reactions of TMP with and without Li are expressed in Fig. 2A. We conducted QM calculations for each of the reactants and products in the half reactions at 298 K and 1 bar. Geometry optimization and frequency calculations were performed for each of the reactants and products using B3LYP-D3(BJ)/6-311G* with solvation model based on density continuum solvation model. B2PLYP-D3(BJ)/def2-TZVP was further used to obtain the single-point energy in gas phase, which was converted to gas-phase Gibbs free energy by adding with the thermal correction from the frequency calculations. M062X/6-31G* was used to obtain the solvation free energy in aqueous phase. The solvation model based on density continuum solvation model with water parameter (*ε* = 78.3553) and acetone (*ε* = 20.493), respectively, was applied during the geometry optimization and frequency calculations and to yield the solvation energy. The calculated thermochemistry values from Gaussian for each of the reactants and products were displayed in Table S7. The absolute Li/Li^+^ electrode potential is approximately 1.4 V. The reduction potential of TMP-derived product versus Li/Li^+^ electrode was calculated as [9,91,92],

$$E^{0}=\left(\mathrm{vs}\frac{{\mathrm{Li}}^{+}}{\mathrm{Li}}\right)=E_{\mathrm{abs}}^{0}-E_{\mathrm{abs}}^{0}\left(\frac{{\mathrm{Li}}^{+}}{\mathrm{Li}}\right)=\frac{-\Delta G_{298K,\text{solv}}}{\mathrm{nF}}-1.4$$ (1)

where *F* is the Faraday’s constant and *n* is the number of electrons (here, *n* = 1).

#### Analysis of MD simulations

Data analysis of MD simulations was performed with AMBER [83,93] and GROMACS [94] built-in analysis tools, or visual molecular dynamics [95] and further generated using Matplotlib [96]. Structural features are shown by PyMOL (Schrödinger, LLC). The RDF defines the ratio of the average local number density of particles, ⟨*ρ*(*r*)⟩, at a distance *r*, to the bulk density of particles, *ρ*: *g*(*r*) = ⟨*ρ*(*r*)⟩/*ρ*. The self-diffusion coefficient *D* for type A can be calculated from the mean-squared displacement (MSD) using the Einstein relation:

$$\begin{array}{c} \begin{array}{l} D=\frac{1}{2d}\underset{t\to \infty }{\text{lim}}\frac{d}{\mathrm{dt}}\langle \mathrm{MSD}\left(t\right)\rangle =\frac{1}{6}\underset{t\to \infty }{\text{lim}}\frac{d}{\mathrm{dt}}\langle {\left[r\left(t\right)-r\left(0\right)\right]}^{2}\rangle \\ \;=\frac{1}{6}\underset{t\to \infty }{\text{lim}}\frac{\sum_{i=1}^{N}{\langle {\left[r_{i}\left(t\right)-r_{i}\left(0\right)\right]}^{2}\rangle }_{i\in A}}{\mathrm{Nt}} \end{array} \end{array}$$ (2)

where *d* (=3) is the dimension of the system, *r_i_* represents the center-of-mass position of molecule *i* at time *t*, *t* = 0 refers to a time reference point, and the angle brackets ⟨ ⟩ denote the ensemble average. Long MD trajectories were split into shorter trajectories from which the time reference points were updated, and then we average over all MSD values. The system-size dependency of self-diffusion of pure liquids is given by [97]:

$$D^{0}=D^{\mathrm{MD}}+D^{\mathrm{FSC}}=D^{\mathrm{MD}}+\frac{2.837k_{B}T}{6\mathrm{\pi \eta L}}$$ (3)

where *D*^0^ is the diffusion in an infinitely large system, *D*^FSC^ is the finite-size correction, 2.837 is a dimensionless constant for a cubic simulation box with periodic boundary conditions, *η* is the shear viscosity of the liquid, and *L* is the length of the simulation box [97,98]. In equilibrium MD, the viscosity can be computed from the Green–Kubo relation or Einstein relation [99,100]. To obtain the off-diagonal stress tensors, we convert the AMBER prmtop and inpcrd files into GROMACS input files using the amb2gro_top_gro.py program and conducted MD simulations using the GROMACS 2019.4 package [94]. The off-diagonal components of the stress tensor were extracted using the gmx energy tool and processed using the Green–Kubo relation [99,100]. The accuracy of the calculated viscosity has been studied previously with the integration time, the early-time range to select reasonable viscosity, the size of the cubic box, and weight [97,101–103], which suggests that long integration time can improve the accuracy of the viscosity at the early-time range near 2 ps. We split long MD trajectories into shorter trajectories and examined different integration lengths and sampling frequencies [99]. The averaged shear viscosity at 6 to 50 ps is extracted from 200-ps calculations (100,000 iterations) before the shear viscosity difference keeps growing between trajectories. The simulated shear viscosity is also close to the experimental data (Fig. S61), verifying the accuracy of the simulation model.

The transference number is computed using the approach built upon concentrated solution theory [33,104,105]. The transport coefficients using the solvent reference are expressed in the Einstein form as:

$$W_{\mathrm{ij}}=\frac{1}{6}\underset{t\to \infty }{\text{lim}}\frac{d}{\mathrm{dt}}\langle \frac{1}{n_{i}}\sum_{a}\Delta r_{i,a}^{s}\left(t\right)\cdot \frac{1}{n_{j}}\sum_{\beta }\Delta r_{j,\beta }^{s}\left(t\right)\rangle \left[\mathrm{SOL}\right]$$ (4)

where *i* and *j* represent different species and *n*_i_ is the number of particles of species *i*. The superscript *s* indicates that we use the average position of all solvent molecules in solution (SOL) as displacement reference. Δ*r_i,α_^s^*(*t*) represents the displacement vectors for the motion of *α*th particle of species *i* from time 0 to *t*.

The cation transference number is computed as:

$${t_{+}}^{\mathrm{SOL}}=\frac{W_{++}-W_{+-}}{W_{++}-2W_{+-}+W_{--}}$$ (5)

The degree of uncorrelated ion motion (*α*), or ionicity, is defined as the ratio of the actual total ionic conductivity (*σ*) and the ideal ionic conductivity (*σ*_NE_):

$$a=\frac{\sigma }{\sigma_{\mathrm{NE}}}=\frac{\sum_{i=1}^{N}\sum_{j=1}^{N}q_{i}q_{j}\langle \Delta r_{i}\left(t\right)\Delta r_{j}\left(t\right)\rangle }{\sum_{i=1}^{n}q_{i}^{2}\langle \Delta r_{i}{\left(t\right)}^{2}\rangle }$$ (6)

The ratio represents the proportion of all the terms in the collective displacement to only the self-correlation term. When *α* = 1 occurs, all ions move in completely uncorrelated motion. If *α* = 0 occurs, it indicates that all of the cations only move together with anions. The correlations between the motion of ions (*α*) were analyzed using the “atomiccorr” in “cpptraj” [93] from AMBER package.

DFT calculations for the analysis of binding energy between water and anions: DFT calculations were conducted under Gaussian 16 package. The B3LYP hybrid functional at 6-31g* level of basis set including the atom–pairwise dispersion (DFT-D3) correction with Becke–Johnson (BJ) damping to optimize geometry. Single-point calculation of every structure was carried out at B3LYP/def2-TZVP including the DFT-D3 (BJ) correction. The binding energy was obtained by the following equation:

$$E_{\text{binding}}=E_{\text{complex}}-E_{\text{anion}}-E_{\text{water}}$$ (7)

## Data Availability

All data are available in the manuscript or the Supplementary Materials or from the authors.
