# Supplementary material for: Methanesulfonate in Phosphate Electrolyte Superstructure Enables Ampere-Hour Practical Aqueous Batteries
Source: Research (Wash D C). 2026 Feb 25;9:1170. doi: 10.34133/research.1170 (PMC12932869; doi:10.34133/research.1170)
Supplement: Supplementary 1 — Figs. S1 to S61 Tables S1 to S7 Movies S1 and S2 [file research.1170.f1.zip › SI-0201.docx]

**Supporting information**

**Methanesulfonate in phosphate electrolyte superstructure enables ampere-hour practical aqueous batteries**

Jian Zhi^1,2*#^, Yunfeng Luo^2#^, Chenyi Liao^3#^, Zhongyi Liu^2#^, Mei Han^1,2^, Kaihang Yue^1^, Lei Zhang^2^, Guohui Li^3,4*^, P. Chen^2,5*^

1. State Key Laboratory of High Performance Ceramics, Shanghai Institute of Ceramics, Chinese Academy of Sciences, Shanghai, 200050, China

2. Department of Chemical Engineering and Waterloo Institute of Nanotechnology, University of Waterloo, N2L 3G1, Canada

3. Laboratory of Molecular Modeling and Design, State Key Laboratory of Molecular Reaction Dynamics, Dalian Institute of Chemical Physics, Chinese Academy of Sciences, Dalian, 116023, P. R. China

4. Interdisciplinary Research Center for Biology and Chemistry, Liaoning Normal University, Dalian, Liaoning 116029, China

5. School of Chemical and Biomolecular Engineering, College of Engineering, Eastern Institute of Technology, Ningbo, Zhejiang, 315200, China.

#These authors contributed equally to this work.

*Email: jzhi@mail.sic.ac.cn, ghli@dicp.ac.cn, p4chen@uwaterloo.ca


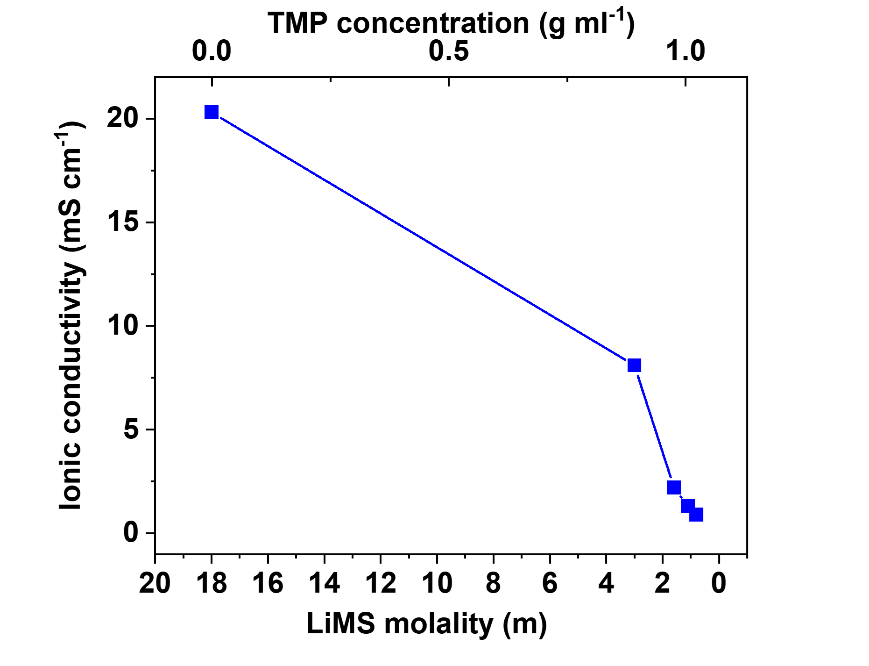


**Figure S1**. Ionic conductivity of various LiMS-H_2_O electrolyte with different concentration of TMP.

**Supplementary Discussion:**

(1) Characterization of Li^+^ transference number (t_Li+_) and diffusion co-efficient (D_Li+_) of various electrolytes.

Li^+^ transference number (t_Li+_) of various LiMS-TMP-H_2_O electrolytes was obtained by AC impedance and potentiostatic DC polarization. Specifically, the time dependence of DC current was measured for LTO/LiMS-TMP-H_2_O/LTO symmetric cells under 20 mV until a stable current was approached. The initial and stable current was extracted, and the cell was monitored by AC impedance to test the bulk resistance of LiMS-TMP-H_2_O and LiMS-TMP-H_2_O/LTO interface. The transference number, t_Li+_ can be calculated by equation 1:

$t_{{Li}^{+}}=\frac{I\left( \infty\right)R_{bulk}\left( \infty\right)[\Delta V-I\left( 0 \right)R_{int}\left( 0 \right)]}{I\left( 0 \right)R_{bulk}\left( 0 \right)[\Delta V-I\left( \infty\right)R_{int}\left( \infty\right)]}$ (1)

where $\Delta V$ is polarization voltage, $I$ is the current, $R_{bulk}$ and $R_{int}$ are the resistance of the LiMS-TMP-H_2_O electrolyte and LiMS-TMP-H_2_O/LTO metal electrode interface, respectively. 0 and $\infty$ represent the initial and steady states, respectively. The I-t curves and Nyquist plots are demonstrated in Figure S2.


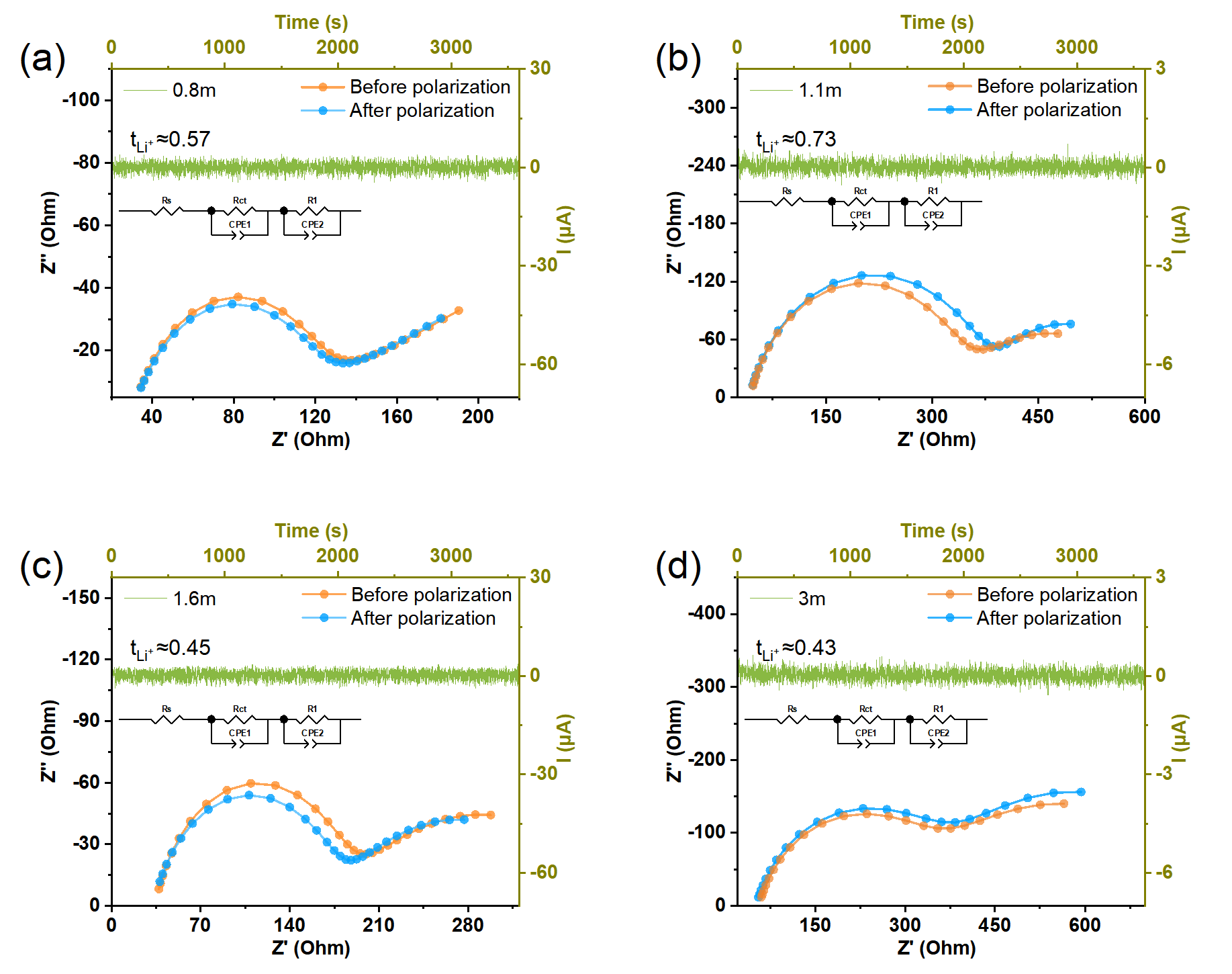


**Figure S2**. Current-time curve at the bias potential of 20 mV and Nyquist plots before and after polarization of LTO//LTO symmetric cells in (a) 0.8m LiMS-TMP-H_2_O, (b) 1.1 m LiMS-TMP-H_2_O, (c) 1.6 m LiMS-TMP-H_2_O and (d) 3 m LiMS-TMP-H_2_O electrolytes.

The Li^+^ diffusion coefficients D_Li+_ were obtained via the galvanostatic intermittent titration technique (GITT, Figure S3), which is typically calculated using the following equation:

$$D_{GITT}=\frac{4}{\pi\tau}({\frac{n_{m}V_{m}}{S})}^{2}(\frac{{\Delta E}_{s}}{{\Delta E}_{t}})^{2}$$

where τ is the relaxation time, $n_{m}$ is the number of moles, $V_{m}$ is the molar volume, *S* is the electrode/electrolyte contact area, ${\Delta E}_{s}$is the total voltage change due to the pulse, and ${\Delta E}_{t}$is the voltage change for constant current charging/discharging.


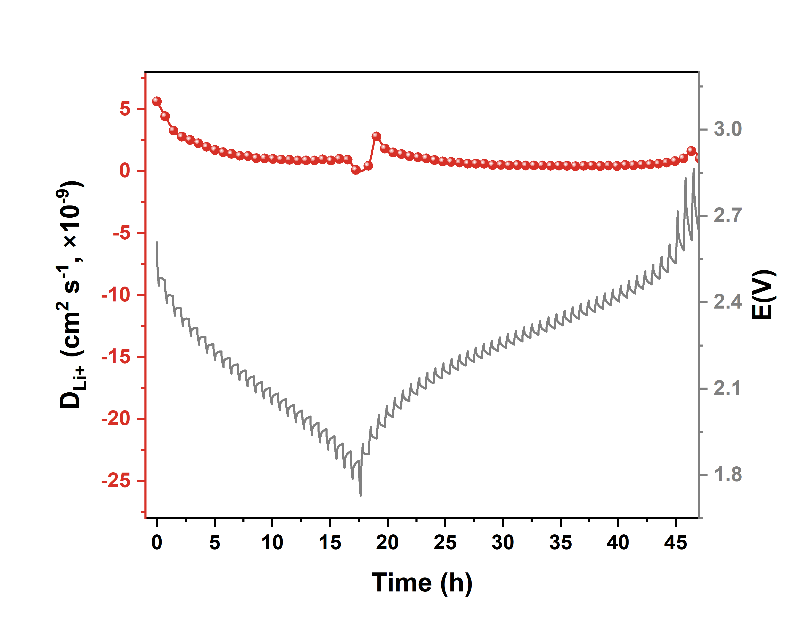


**Figure S3**. Discharge and charge GITT curves of LMO/LTO in 1.1 m LiMS-TMP-H_2_O electrolyte and its corresponding diffusion coefficients at different discharge and charge stages.

(2) Detailed procedure in PFG NMR diffusion measurements:

All the samples were sealed inside a glove box into low-pressure/vacuum-valve NMR tubes (535-LPV-7, Wilmad-Lab Glass). NMR experiments were carried out using a Bruker Avance III HD NMR spectrometer with a resonance proton frequency of 600 MHz and a 5 mm double-resonance broadband probe. ^7^Li diffusion coefficient measurements were conducted using the bipolar gradient longitudinal eddy current delay pulse sequence. The strength of the field gradient (g) was increased in eight equidistant steps up to a maximum of 45 G cm^-1^, and the length of the gradient pulse (δ) and diffusion time (Δ) were adjusted to the experimental conditions (observed nucleus and sample temperature) depending on the expected diffusion coefficient. Diffusion coefficient values were obtained from fits of the signal attenuation to the parameters of the pulse sequence according to Stejskal’s and Tanner’s equation using the Bruker Dynamics Center 2.2.1 software.

(3) Experimental details of DEMS characterization:

To quantify gas evolution associated with SEI formation while excluding contributions from residual or physically adsorbed species, differential electrochemical mass spectrometry (DEMS) was applied using gastight Swagelok-type cells. LMO/LTO full cells were assembled by sequentially stacking an LTO anode, separator, LMO cathode, and a stainless-steel ring spacer, followed by the addition of 85 μL of either LiMS–TMP–H₂O or pristine LiMS–H₂O electrolyte. All assembly procedures were conducted under an inert nitrogen atmosphere.

After cell construction, the full cells were interfaced with the DEMS system for simultaneous electrochemical cycling and gas analysis. Electrochemical control was achieved using a battery testing station, while evolved gases were transported through a regulated flow path to a quadrupole mass spectrometer. Prior to electrochemical activation, the assembled cells were purged with high-purity argon at approximately atmospheric pressure overnight to remove dissolved gases from the electrolyte and desorb residual species from internal cell components. As a result, only gases generated during electrochemical reactions were detected during subsequent cycling. To further suppress moisture interference, a cold trap was installed at the gas outlet before mass spectrometric analysis.

(4) Cost calculations:

The calculation of costs is based on the price per liter of electrolyte ($USD L ^-1^).

$$Cost=\frac{Price \left( USD \right)}{Volume \left( L \right)},$$

$$Price=salt price+additives price+water price \left( negligible \right)$$

For example, when we calculate the cost of WIS electrolyte (21 mol LiTFSI in 1 kg water), we can regard the volume of the electrolyte prepared this way as 1 L, and the corresponding cost can be calculated as follows:


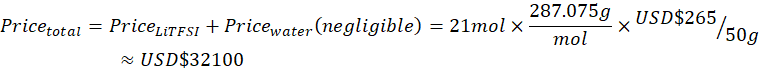

$$Cost=\frac{Price \left( USD \right)}{Volume \left( L \right)}=\frac{USD\$32100}{1L}=\frac{32100 USD\$}{L}$$

The raw material prices used in the cost analysis were based on the real-time market prices for laboratory-grade chemicals at the time of calculation, which has been listed as Table S1.

**Table S1.** Detailed prices and specifications of all key raw materials for the cost calculations

| **Product Name** | **Chemical Formula** | **Price*** |
| --- | --- | --- |
| Zinc sulfate monohydrate | ZnSO_4_ · H_2_O | USD$190.70/500g |
| Lithium sulfate | Li_2_SO_4_ | USD$113.18/500g |
| Manganese sulfate monohydrate | MnSO_4_ · H_2_O | USD$97.67/1Kg |
| Lithium bis(trifluoromethanesulfonyl)imide （LiTFSI） | LiN(CF_3_SO_2_)_2_ | USD$265.03/50g |

* Based on the prices from Sigma-Aldrich website (<https://www.sigmaaldrich.com>) on 10^th^-May-2025

We acknowledge that factors such as production yield losses, procurement scale, material purity grades, and process-dependent costs would significantly influence the final cost in large-scale manufacturing and practical applications. However, the primary purpose of this comparison is to provide a preliminary and relative cost assessment among different electrolyte formulations at the laboratory research and development stage, thereby evaluating their initial economic feasibility. To this end, a consistent and idealized calculation framework was adopted across all electrolyte systems to ensure a fair and meaningful comparison under identical assumptions, rather than to predict absolute costs in real-world production scenarios.


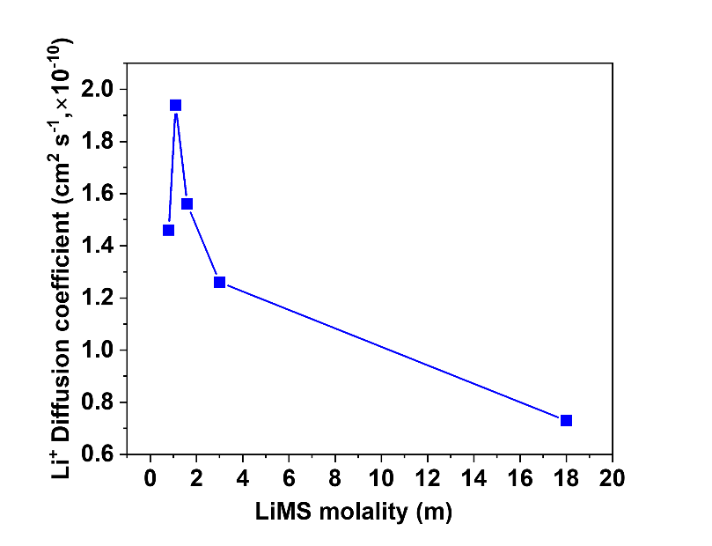


**Figure S4.** Diffusion coefficients of Li^+^ based on PFG-NMR technology.


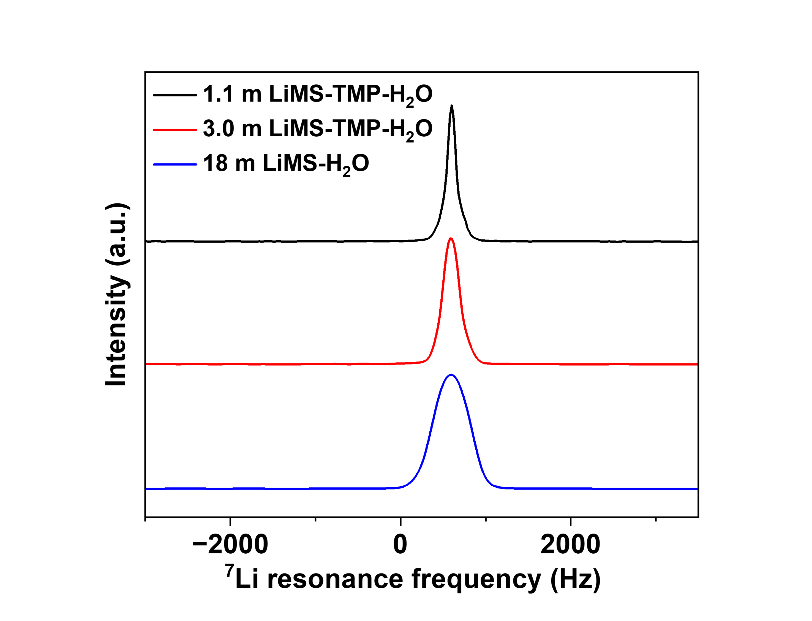


**Figure S5**. 7Li NMR spectrum of 1.1 m LiMS-TMP-H_2_O, 3.0 m LiMS-TMP-H_2_O and 18 m LiMS-H_2_O at 298 K.

**
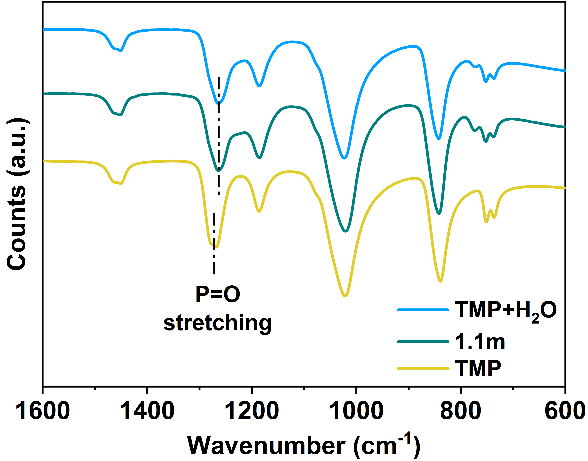
**

**Figure S6.** FT-IR spectra of TMP-H_2_O solution, 1.1 m LiMS-TMP-H_2_O electrolyte and pure TMP.


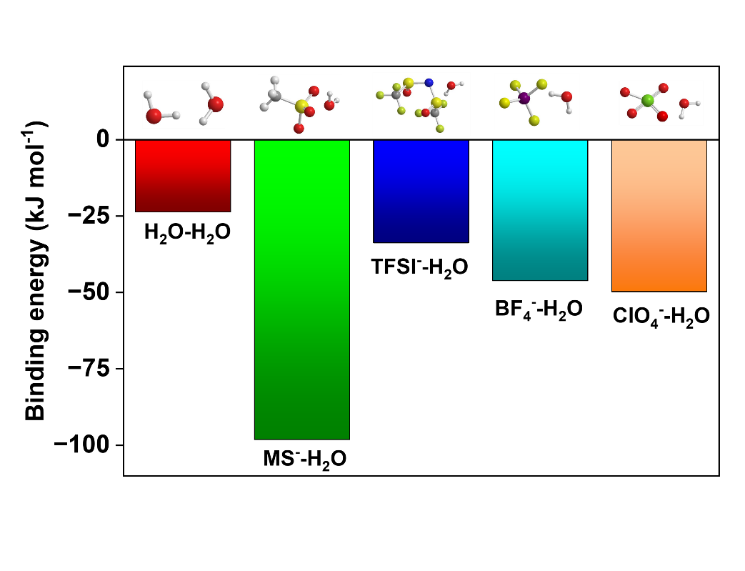


**Figure S7.** Binding energy of water to MS^-^, TFSI^-^. BF_4_^-^ and ClO_4_^-^ anions.


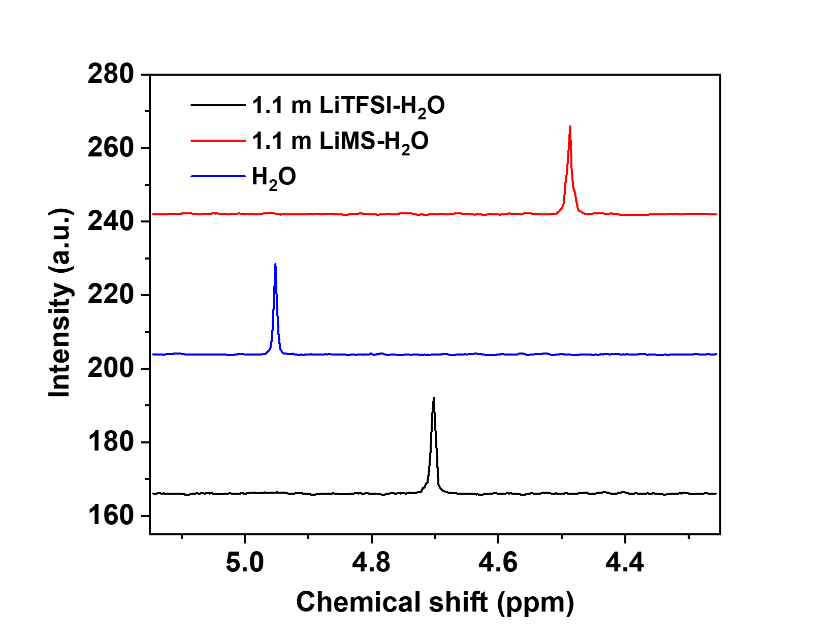


**Figure S8.** ^1^H NMR spectra of water molecules in water, 1.1 m LiMS-H_2_O and 1.1 m LiTFSI-H_2_O solutions.


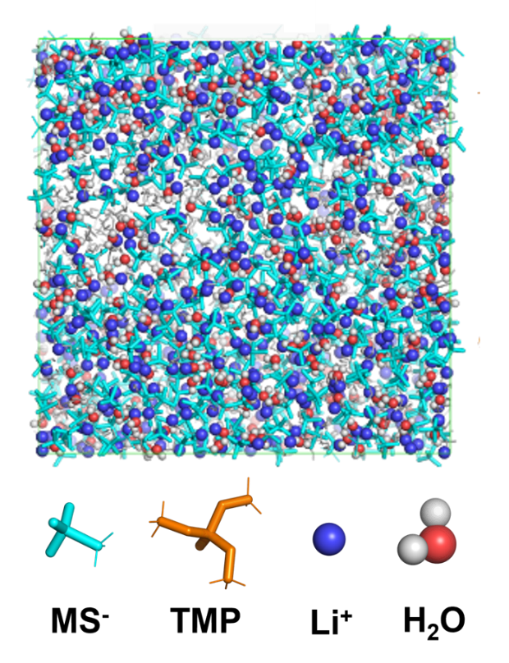


**Figure S9**. Snapshots of the well equilibrated 18 m LiMS-H_2_O systems from MD simulations.


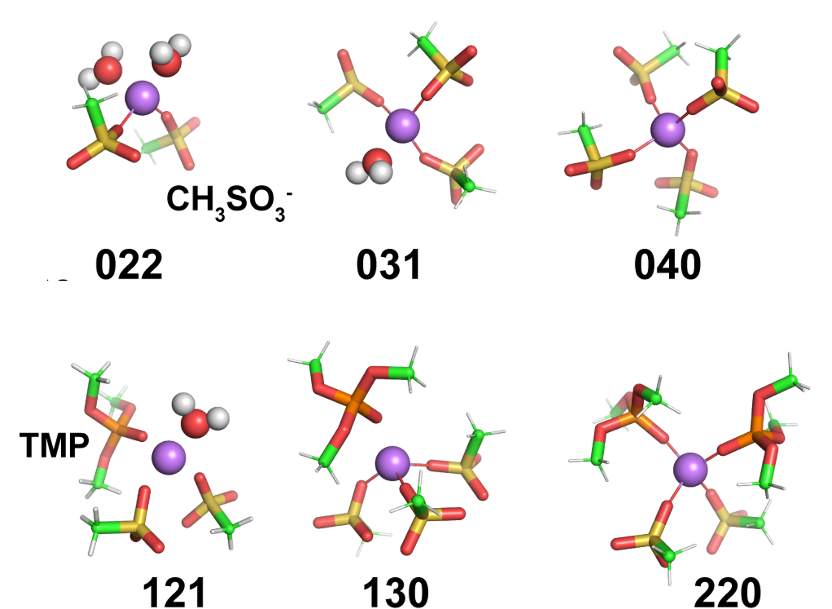


**Figure S10**. Representative coordination structures between Li^+^ and TMP, MS, H_2_O c with high population.


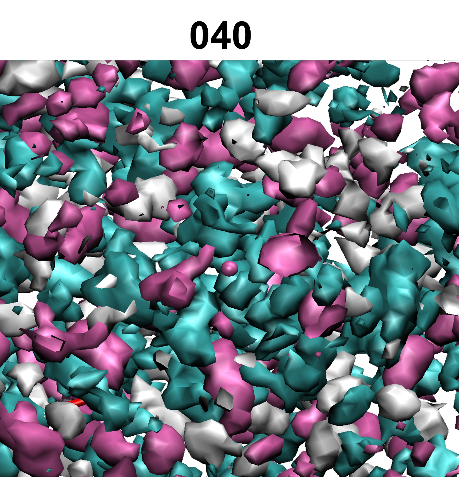


**Figure S11**. Spatial distribution functions (SDFs) of MS (cyan), TMP (yellow), and H_2_O (gray) around a Li^+^ (purple) in 040 coordination structures.


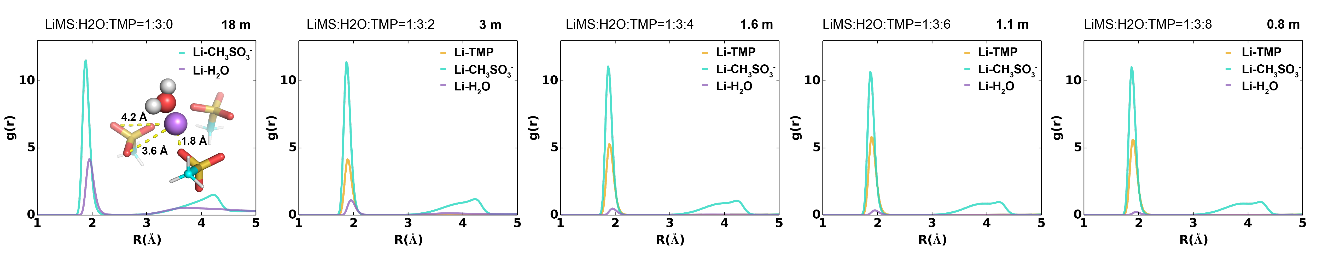


**Figure S12.** Radial distribution function *g*(r) of MS, TMP, and H_2_O as a function of distance from Li^+^. The bulk water *g*(r) as 1 is used as reference.


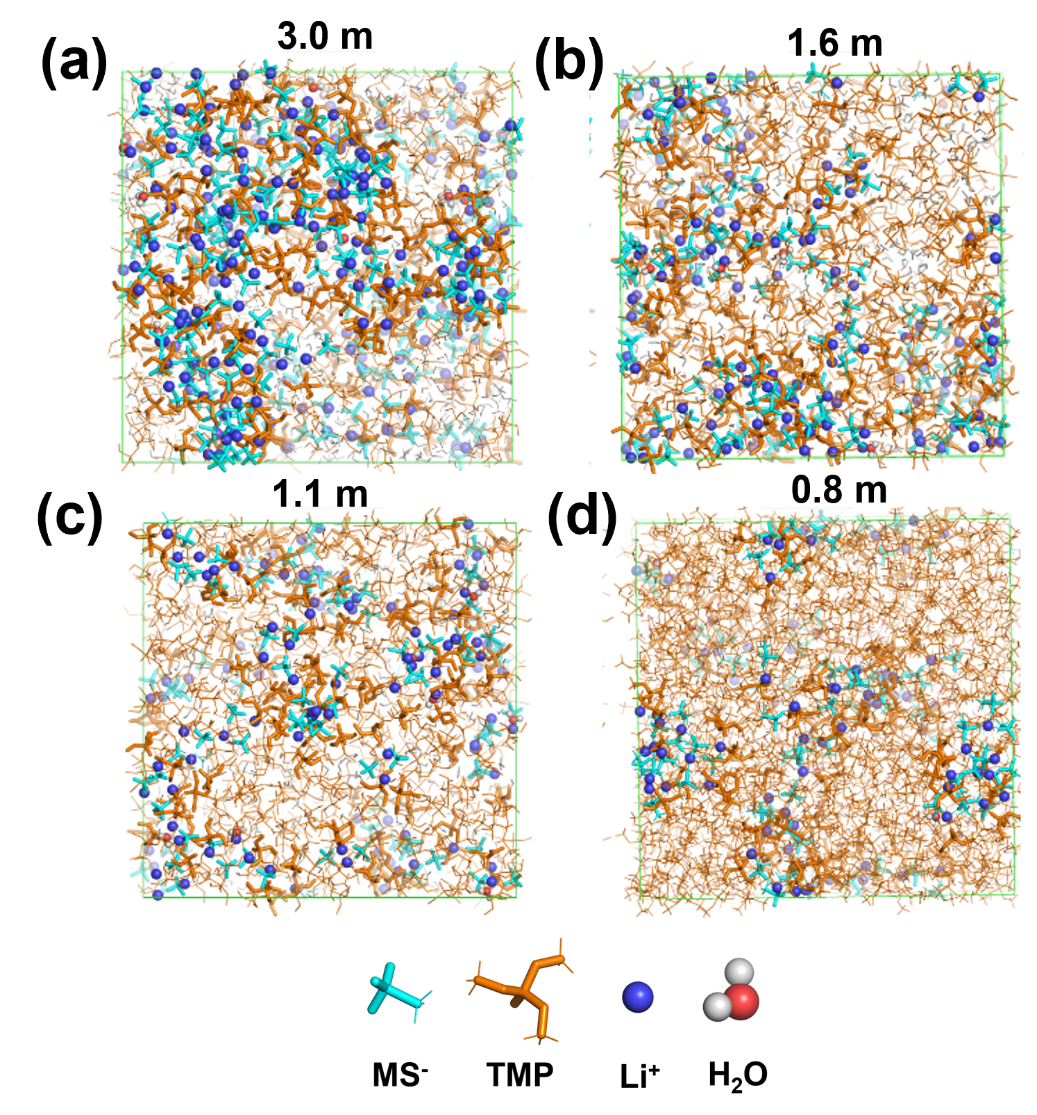


**Figure S13**. Snapshots of the well equilibrated (a) 3 m, (b) 1.6 m, (c) 1,1 m and (d) 0.8 m LiMS-TMO-H_2_O systems from MD simulations.


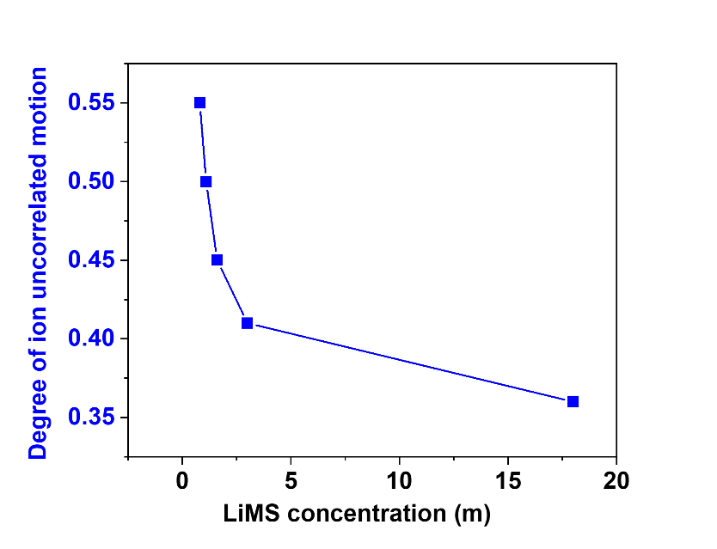


**Figure S14**. Degree of ion uncorrelated motion in various LiMS-H_2_O electrolyte with different concentration of TMP obtained from MD simulations. Values with statistical errors are listed in Table S5.


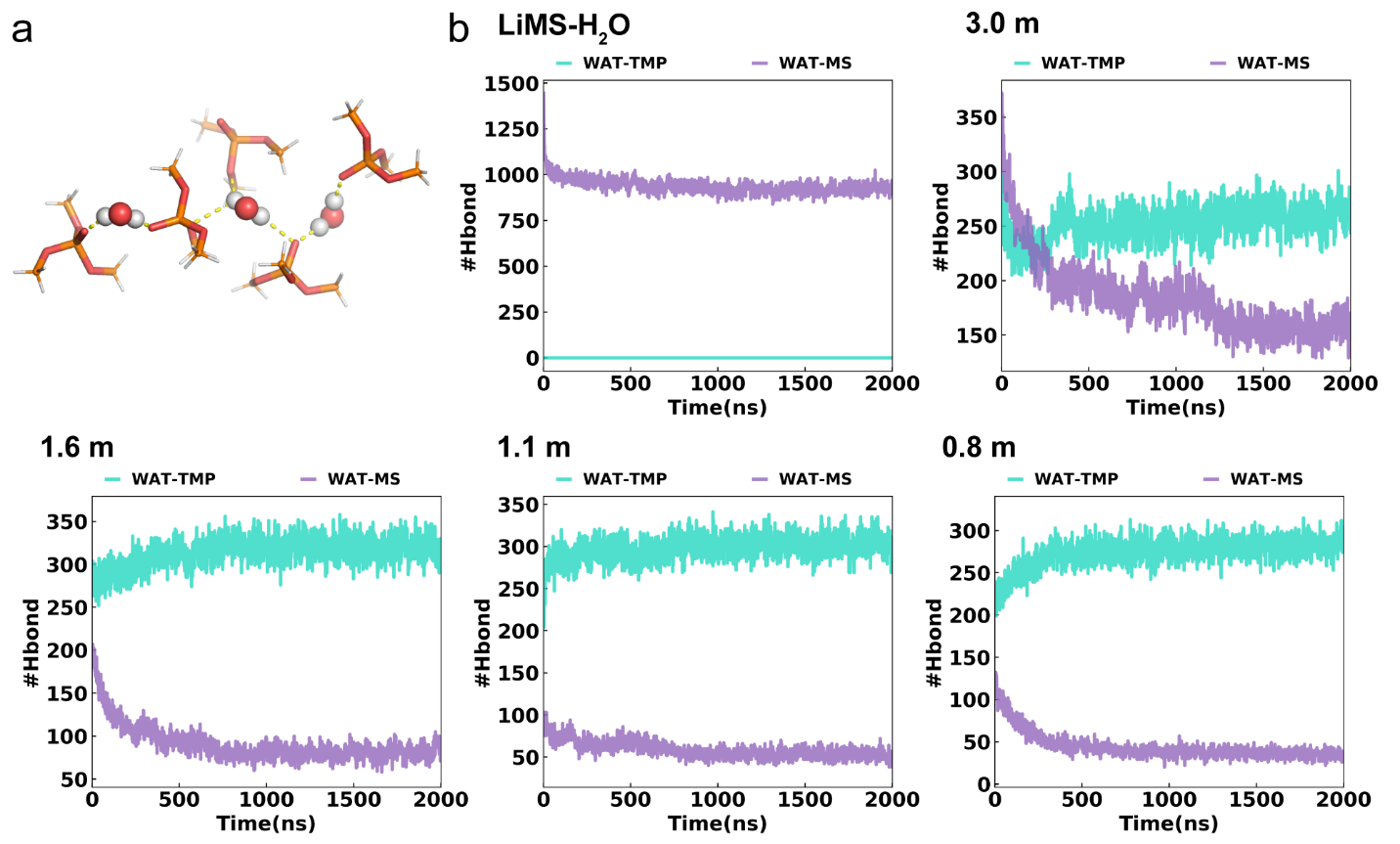


**Figure S15**. (a) Snapshot of hydrogen bonds between TMP and water molecules. (b) Time evolution of hydrogen bonds between TMP and water molecules (in cyan) and hydrogen bonds between MS and water molecules (in purple) in various LiMS-H_2_O electrolyte with different concentration of TMP.


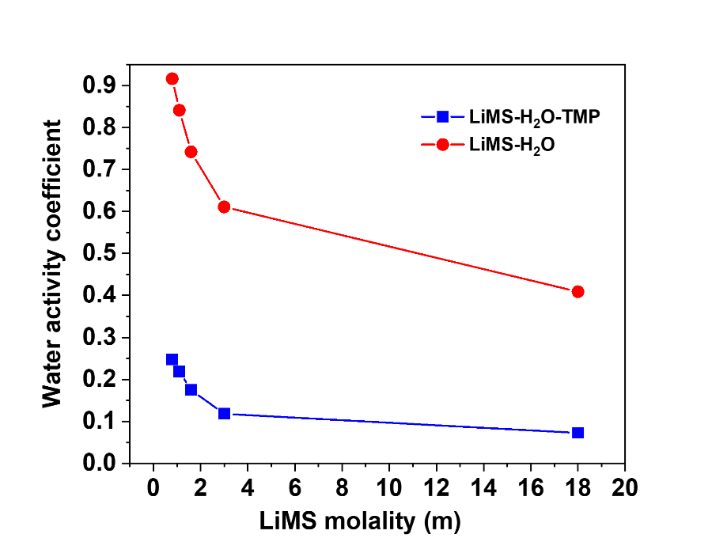


**Figure S16**. Water activity coefficient of LiMS-H_2_O-TMP and pristine LiMS-H_2_O electrolyte with different LiMS concentration.


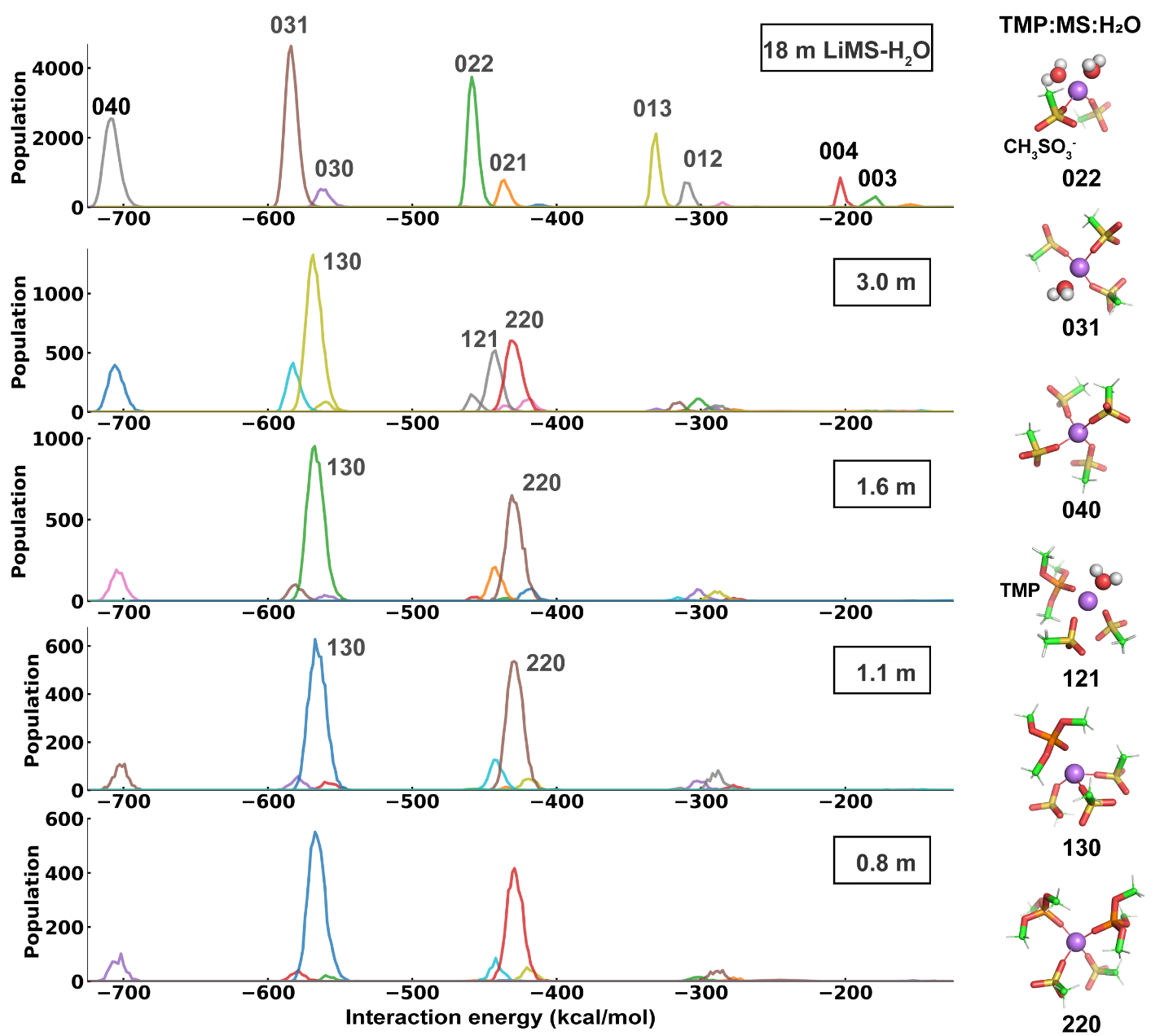


**Figure S17**. Distribution of interaction energies of Li coordination structures in different LiMS systems. Each coordination structure has its characteristic interaction energy peak and shown by different colors. Dominant coordination structures are displayed on the right. The interaction energy of a Li coordination structure includes all bonded and nonbonded interactions and is calculated directly with the GAFF forcefield in MD simulation setup.


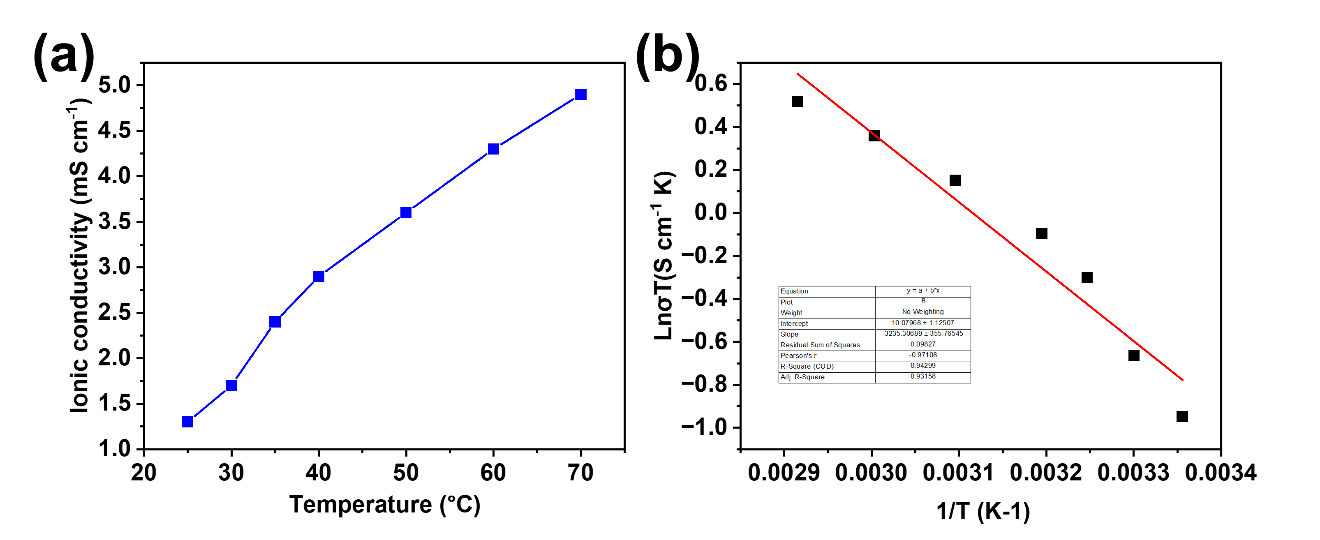


**Figure S18.** (a) Ionic conductivity of 1.1 m LiMS-TMP-H_2_O electrolytes from 25°C to 70°C. (b) Calculation of activation energy based on Arrhenius equation.


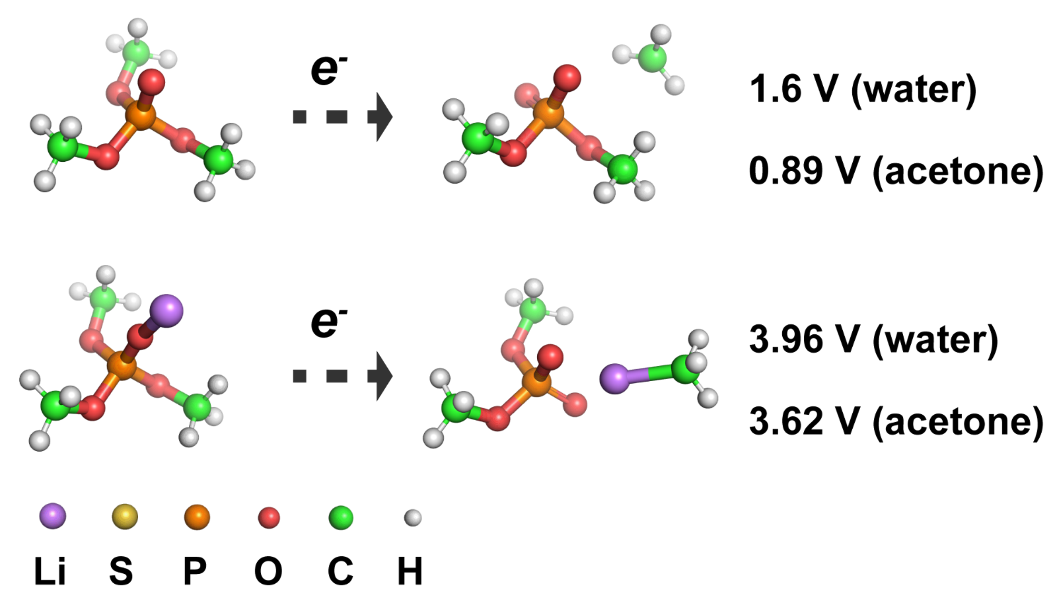


**Figure S19**. Calculated reduction potential of TMP with and without Li^+^ around using quantum mechanics.


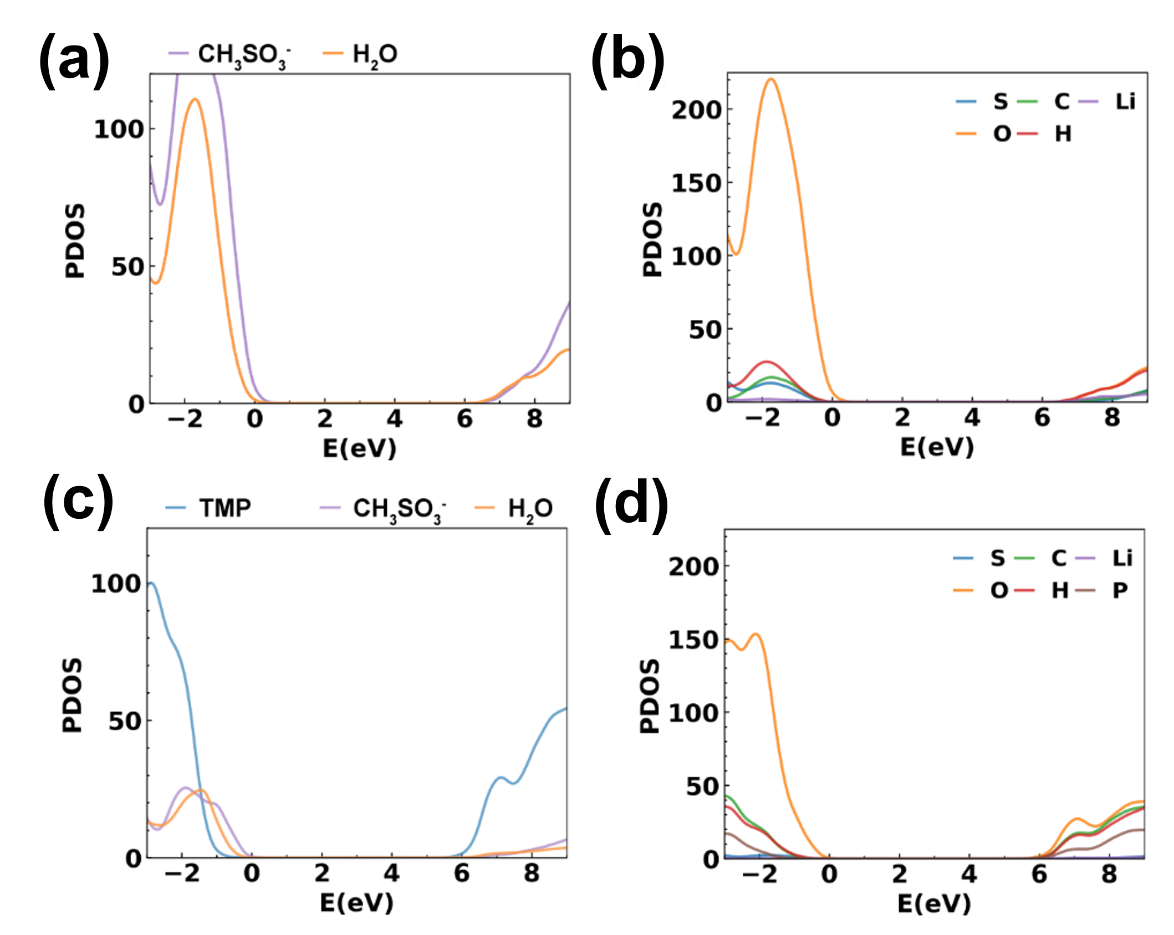


**Figure S20**. PDOS with projections onto TMP, MS, and H_2_O components and projections onto atomic orbitals in (a,b) 18 m LiMS-H_2_O and (c,d) 1.1 m LiMS-TMP-H_2_O using the HSE06 hybrid functional.


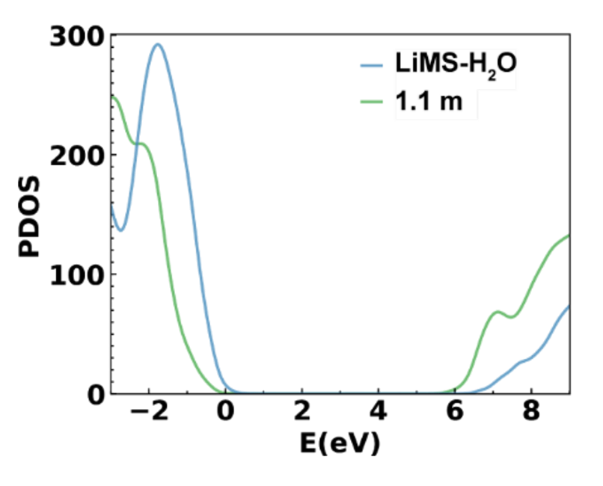


**Figure S21**. Sum of all orbital PDOS in in 18 m LiMS-H_2_O and 1.1 m LiMS-TMP-H_2_O. TMP has caused a significant downshift of the orbital levels and accounts for the vast majority of the LUMO; also it has changed the electron density states near the highest occupied molecular orbital (HOMO) levels.


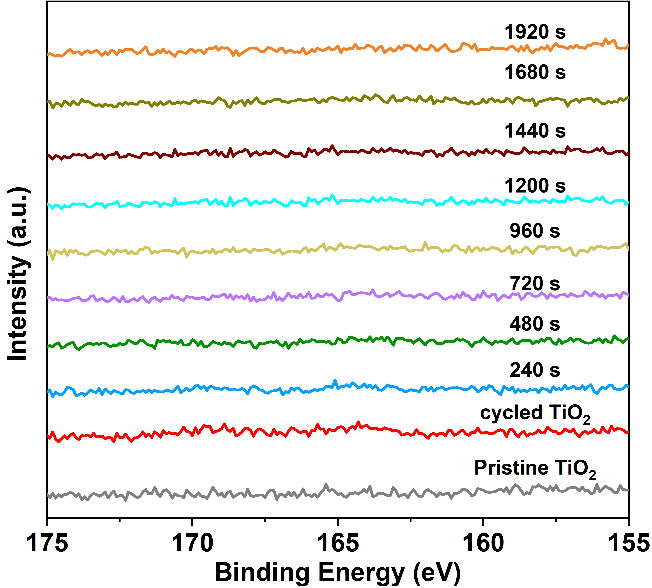


**Figure S22**. S 2p XPS spectrum of pristine (bottom) and cycled TiO_2_ anode at full lithiation state after various durations of Ar^+^ sputtering.


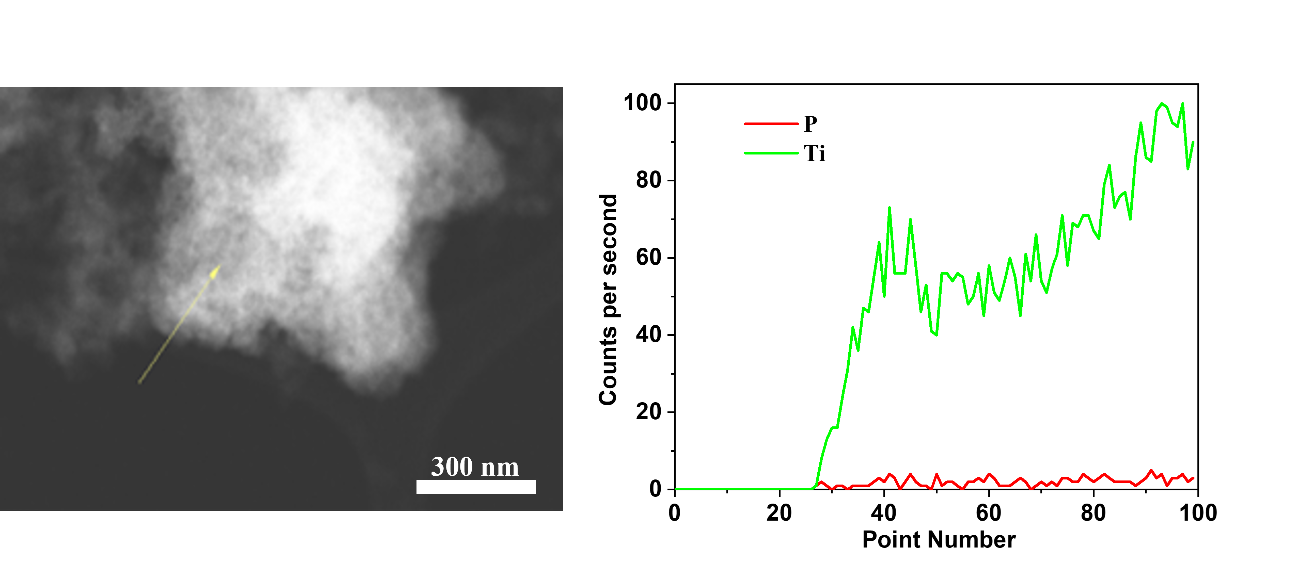


**Figure S23**. EDS line scan of cycled TiO_2_ anode at full lithiation state.


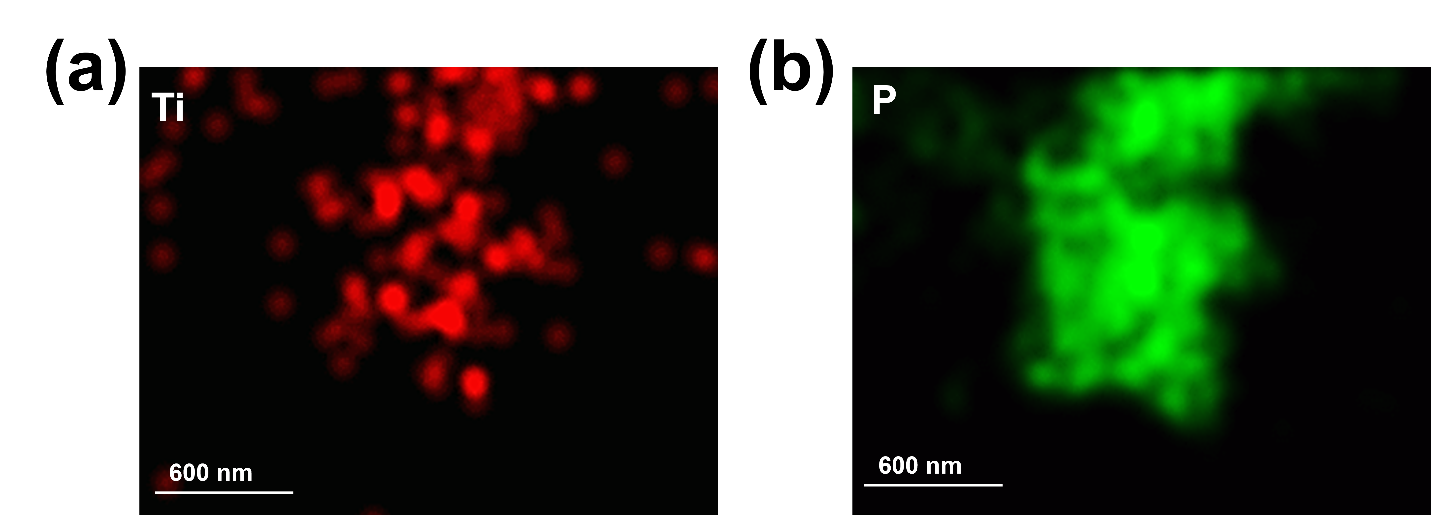


**Figure S24**. EDS mapping of (a) Ti and (b) P elemental distribution of TiO_2_ anode after 50^th^ cycle from LMO/LTP battery with 1.1 m LiMS-TMP-H_2_O electrolyte.


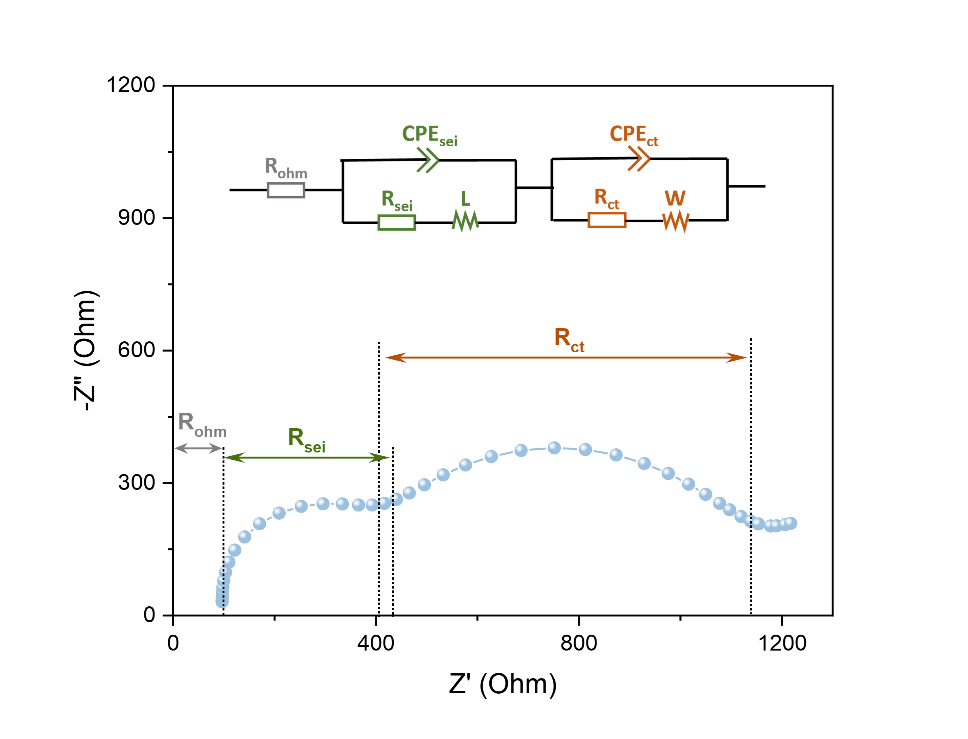


**Figure S25**. Equivalent circuit model for the fitting of the Nyquist plot in Figure 3a.


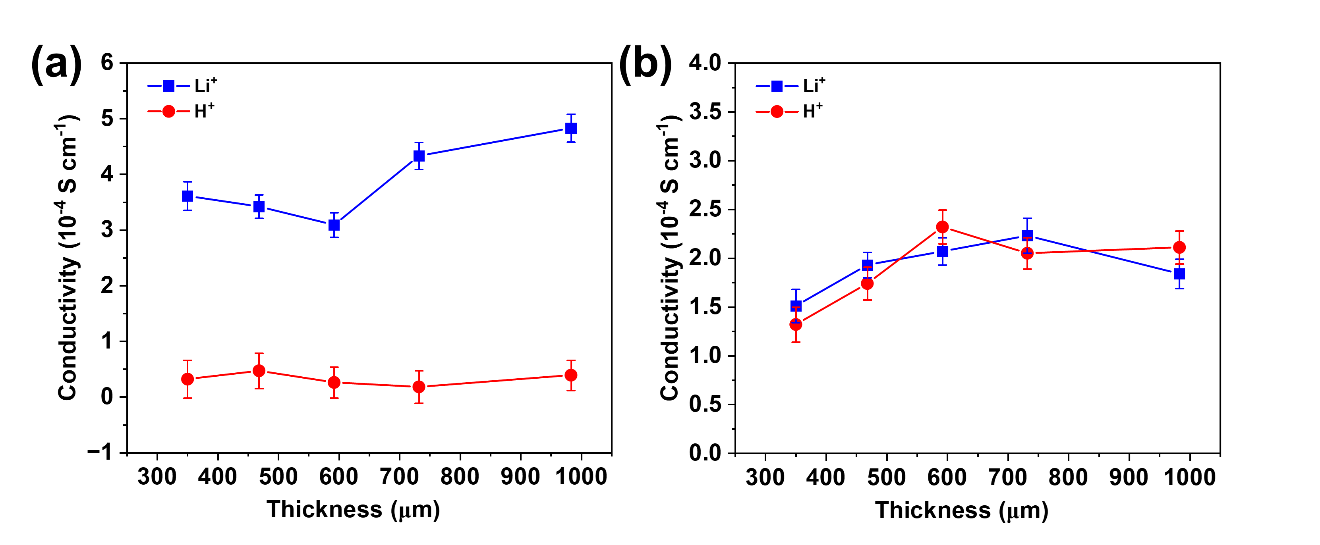


**Figure S26.** Li^+^ and H^+^ ionic conductivities of micrometer-scale ultrathin γ-Li_3_PO_4_ (a) and LiF (b) pellets under different thicknesses.


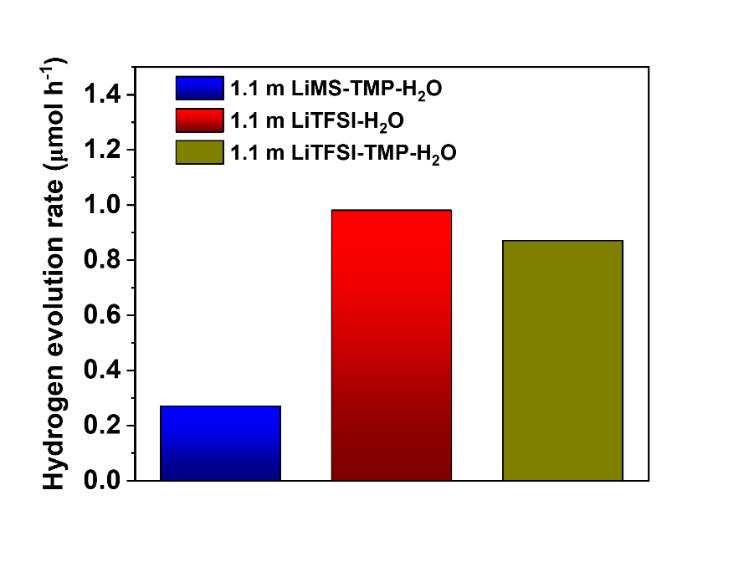


**Figure S27**. The average hydrogen evolution rate of LMO/TiO_2_ battery with 1.1 m LiMS-TMP-H_2_O, 1.1 m LiTFSI-H_2_O and LiTFSI-TMP-H_2_O electrolyte during the cycling under 5 C.


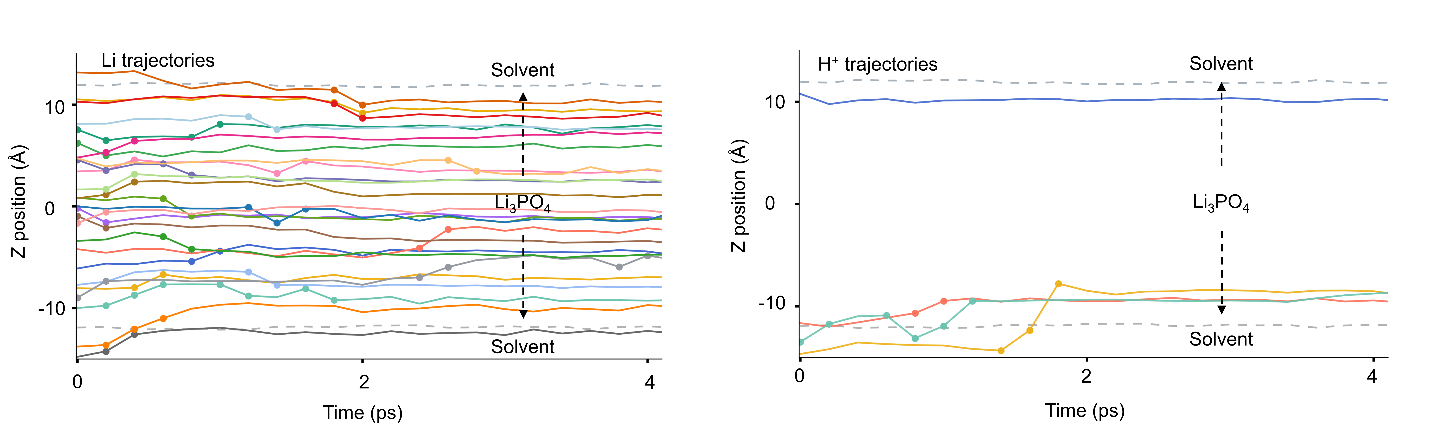


**Figure S28**. Illustrations of Li^+^ or H^+^ transport trajectories in amorphous Li_3_PO_4_ slabs with vacant Li-sites during MD simulations. Each diffusion that is greater than 0.9 Å in 0.2 ps is marked by dots. The positions of the upper and lower interfaces of Li_3_PO_4_ slabs are marked by two gray lines.


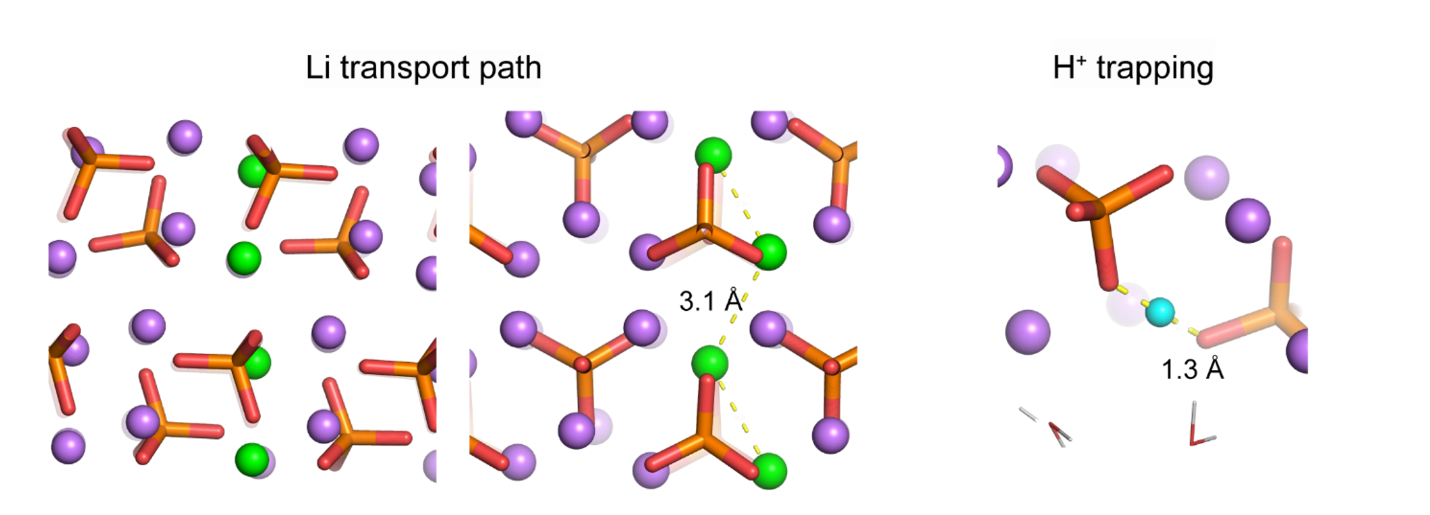


**Figure S29**. The Li^+^ diffusion pathway and H^+^ trapped at the interface of Li_3_PO_4_.


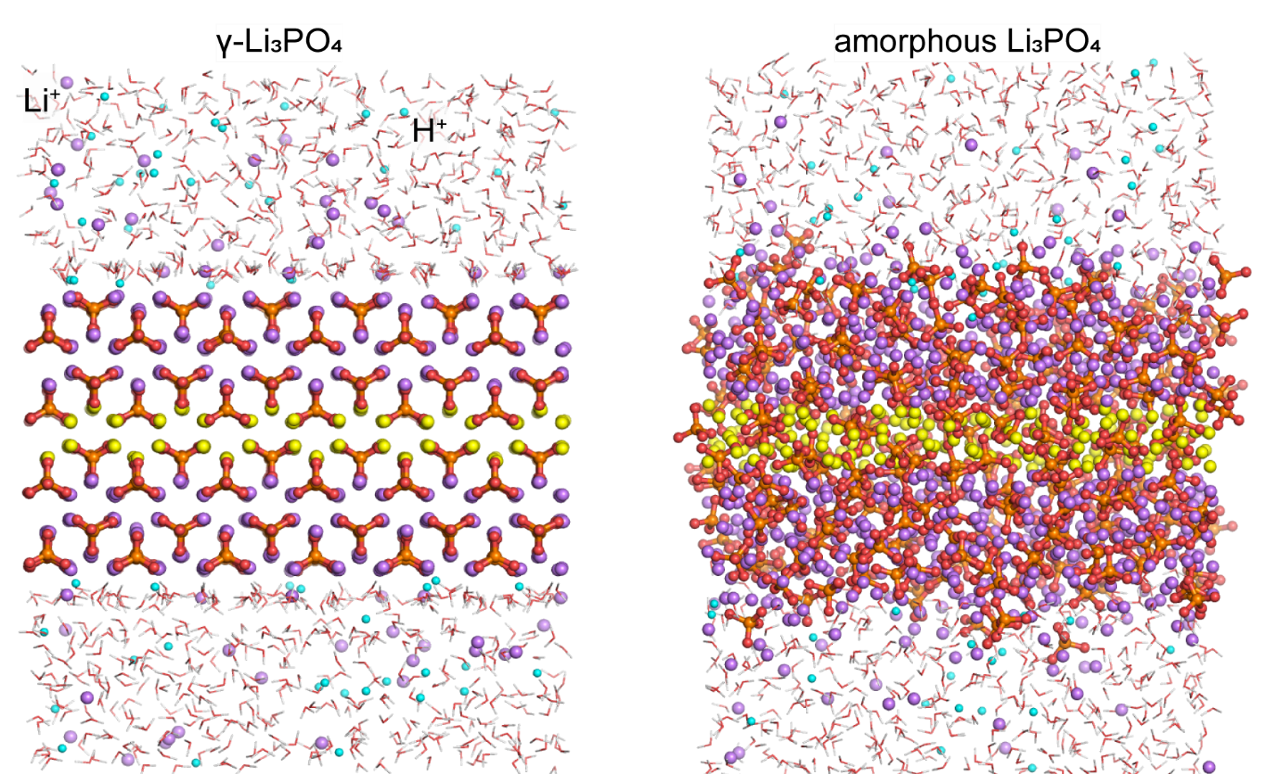


**Figure S30**. MD equilibrated structures of γ-Li_3_PO_4_ and amorphous Li_3_PO_4_ slabs in aqueous solution of a mixture of LiCl and HCl. Li^+^ is shown in purple sphere; H^+^ is shown in cyan sphere. Vacant Li-sites were simulated by removing random Li^+^ from the middle region of the slab (shown in yellow).


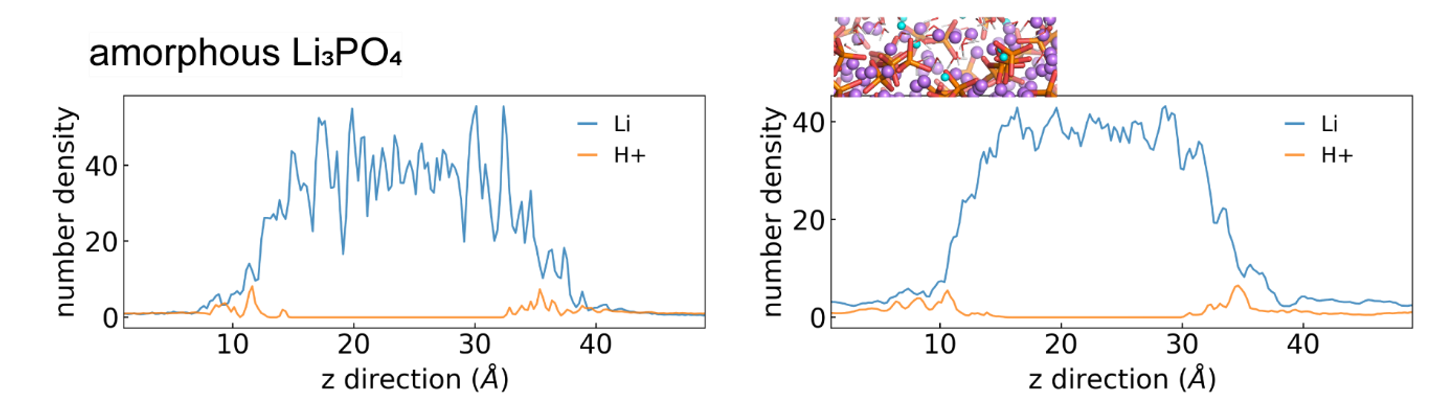


**Figure S31**. Number density of Li^+^ and H^+^ from 0.25-Å slices on z-axis in amorphous Li_3_PO_4_ slabs without (left) and with (right) vacant Li-sites during MD simulations.


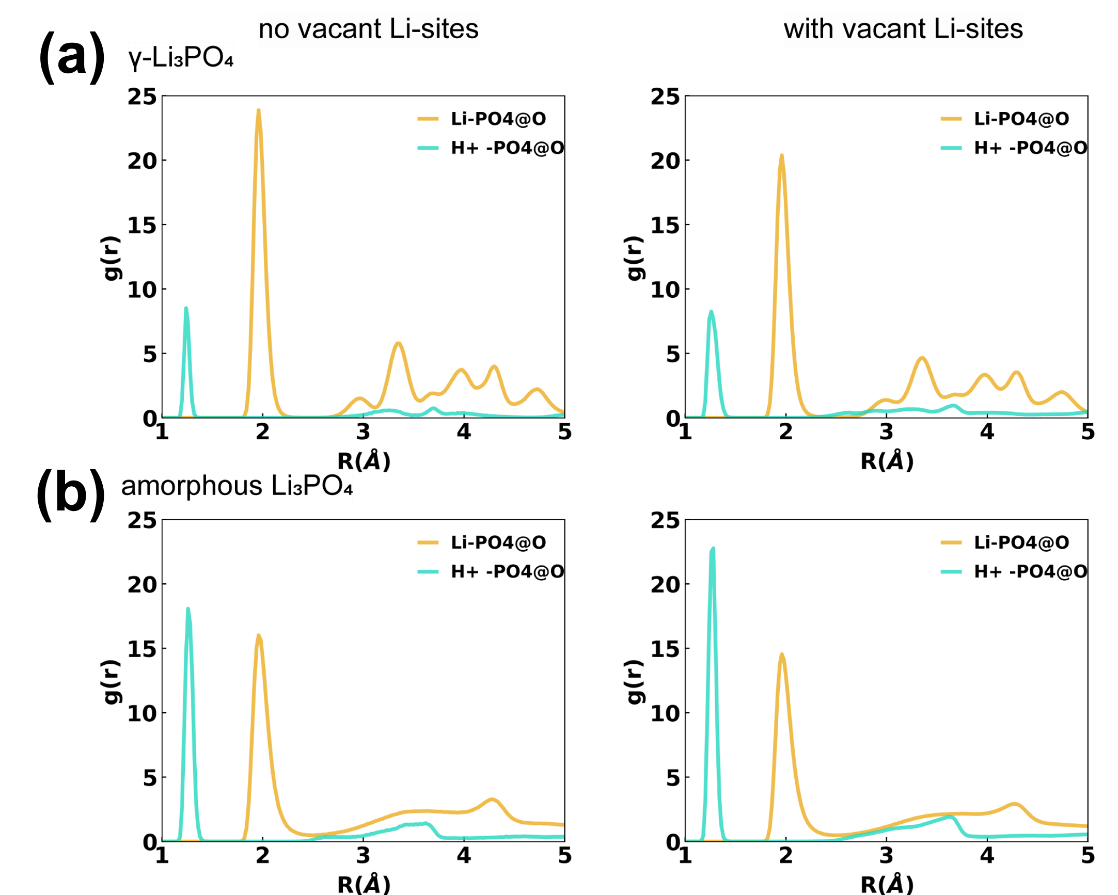


**Figure S32**. Radial distribution function *g*(r) of Li^+^ and H^+^ from TMP@O in (a) γ-Li_3_PO_4_ and (b) amorphous Li_3_PO_4_ slabs with and without vacant Li-sites during MD simulations.


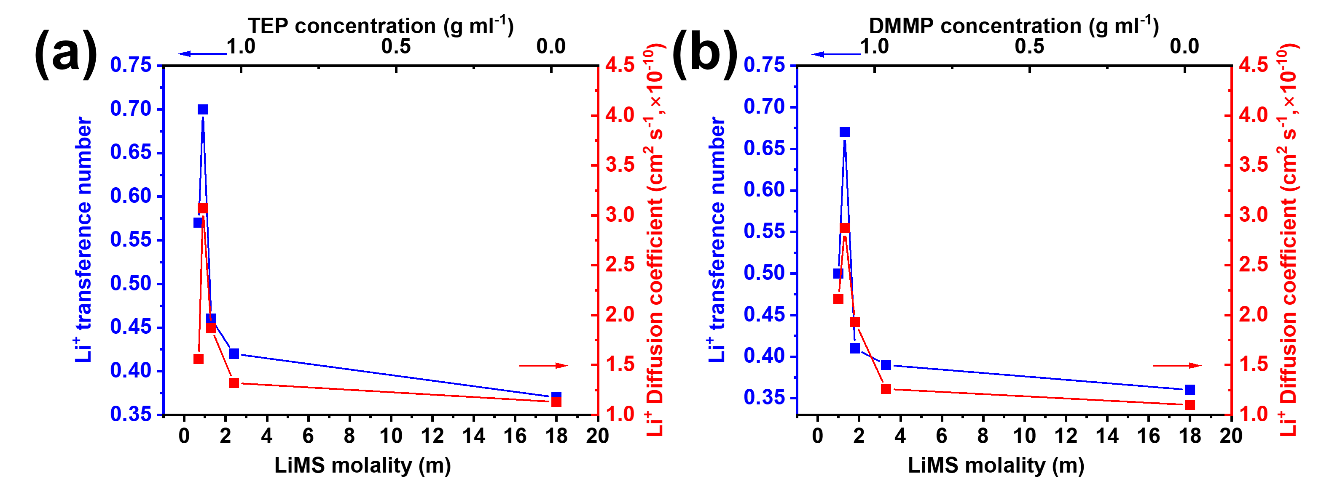


**Figure S33.** Li^+^ transference number (t_Li+_) and diffusion co-efficient (D_Li+_) of (a) LiMS-TEP-H_2_O and (b) LiMS-DMMP-H_2_O electrolytes under different LiMS modalities and phosphate concentrations.


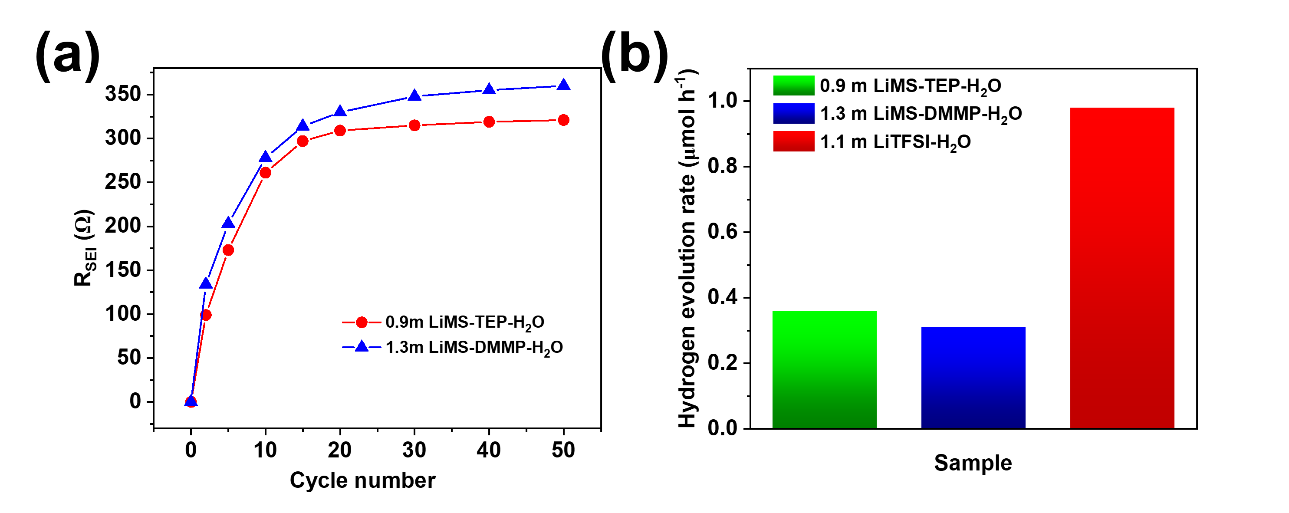


**Figure S34.** (a) The change of R_sei_ as a function of cycling number of TiO_2_ anode from LMO/TiO_2_ battery with 1.1 m LiMS-TMP-H_2_O, 0.9 m LiMS-TEP-H_2_O and 1.3 m LiMS-DMMP-H_2_O electrolytes. (b) The average H_2_ evolution rate of LMO/TiO_2_ battery with 0.9 m LiMS-TEP-H_2_O, 1.3 m LiMS-DMMP-H_2_O and 1.1 m LiTFSI-H_2_O electrolyte during the cycling under 5 C.


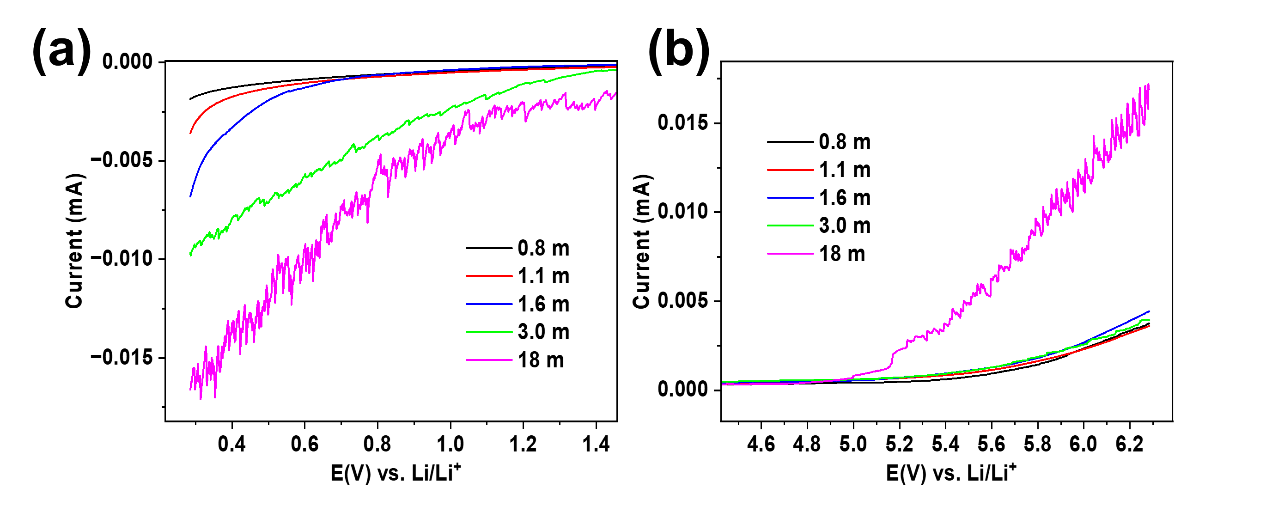


**Figure S35**. The closer view of the areas near the (a) cathodic and (b) anodic limits in Figure 5a.

**
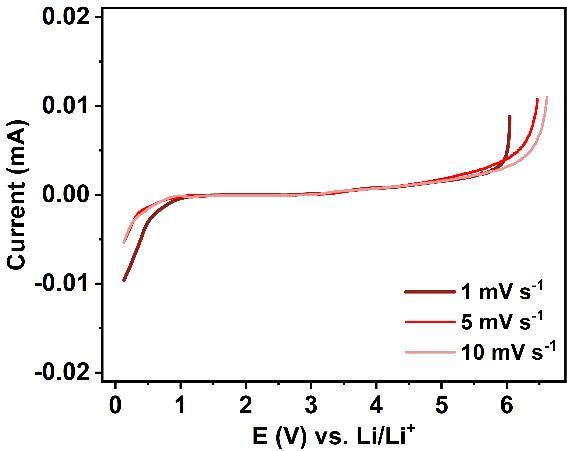
**

**Figure S36.** Electrolyte stability window of 1.1 m LiMS-TMP-H_2_O electrolyte with different scan rates.


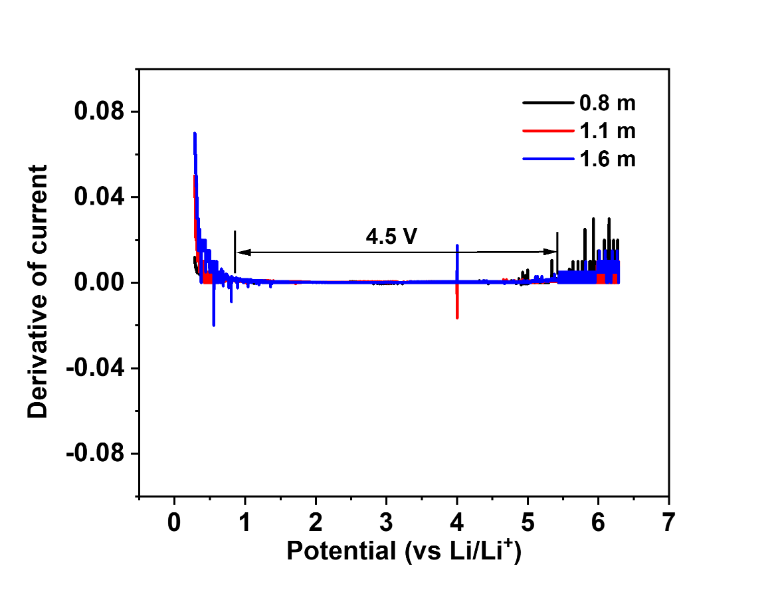


**Figure S37**. Derivative of the current (Figure 5a) from 1.6 m, 1.1 m and 0.8 m electrolyte.


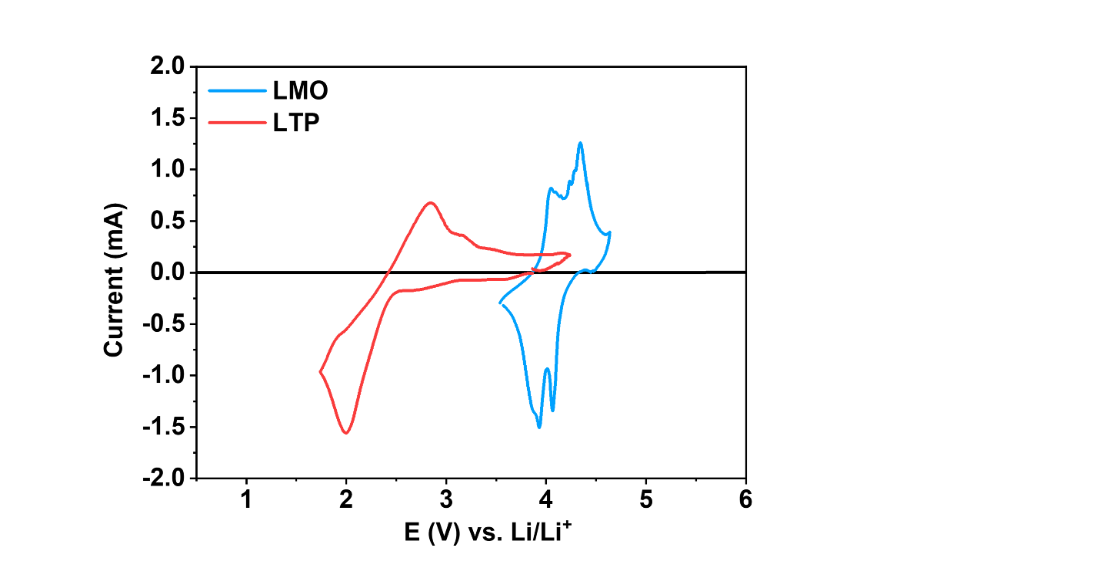


**Figure S38**. Electrochemical stability window of 1.1 m LiMS-TMP-H_2_O electrolyte overlaid with cyclic voltammograms of LMO/LTP couple at a scan rate of 0.2 mV s^-1^.


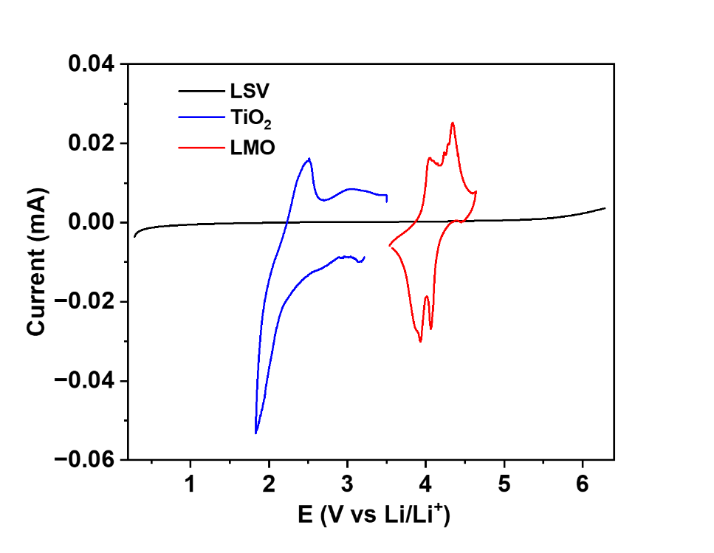


**Figure S39**. Electrochemical stability window of 1.1 m LiMS-TMP-H_2_O electrolyte overlaid with cyclic voltammograms of LMO/TiO_2_ couple at a scan rate of 0.2 mV s^-1^.


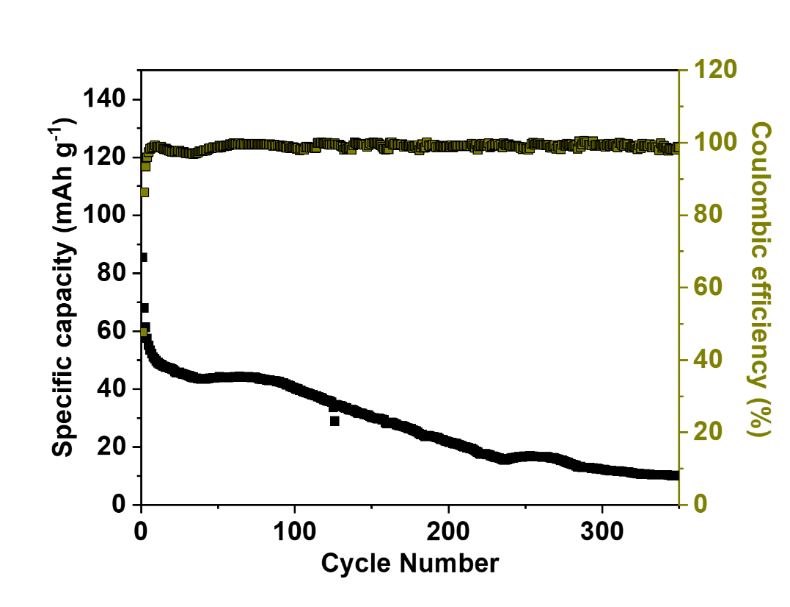


**Figure S40**. Cycling performance of LMO/LTO aqueous battery with 1.1 m LiTFSI-TMP-H_2_O electrolyte at 5 C.


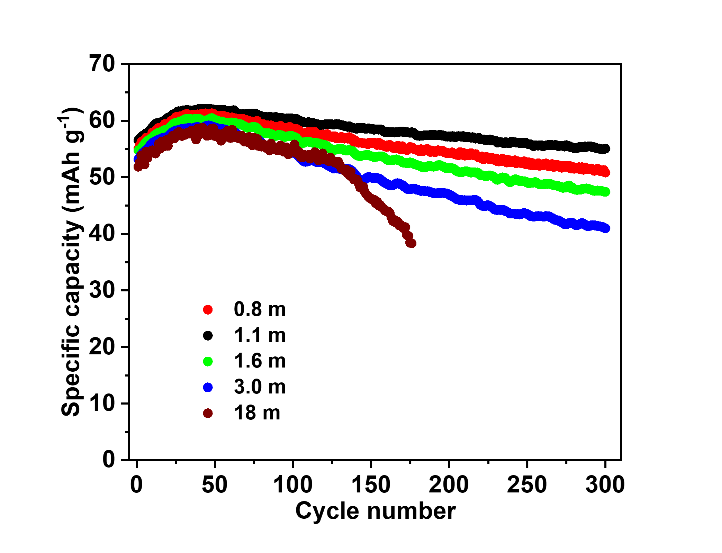


**Figure S41.** Cycling performance of LMO/LTO aqueous battery with 0.8 m, 1.1 m, 1.6 m, 3 m LiMS-TMP-H_2_O and 18 m LiMS-H_2_O electrolyte at 3 C.


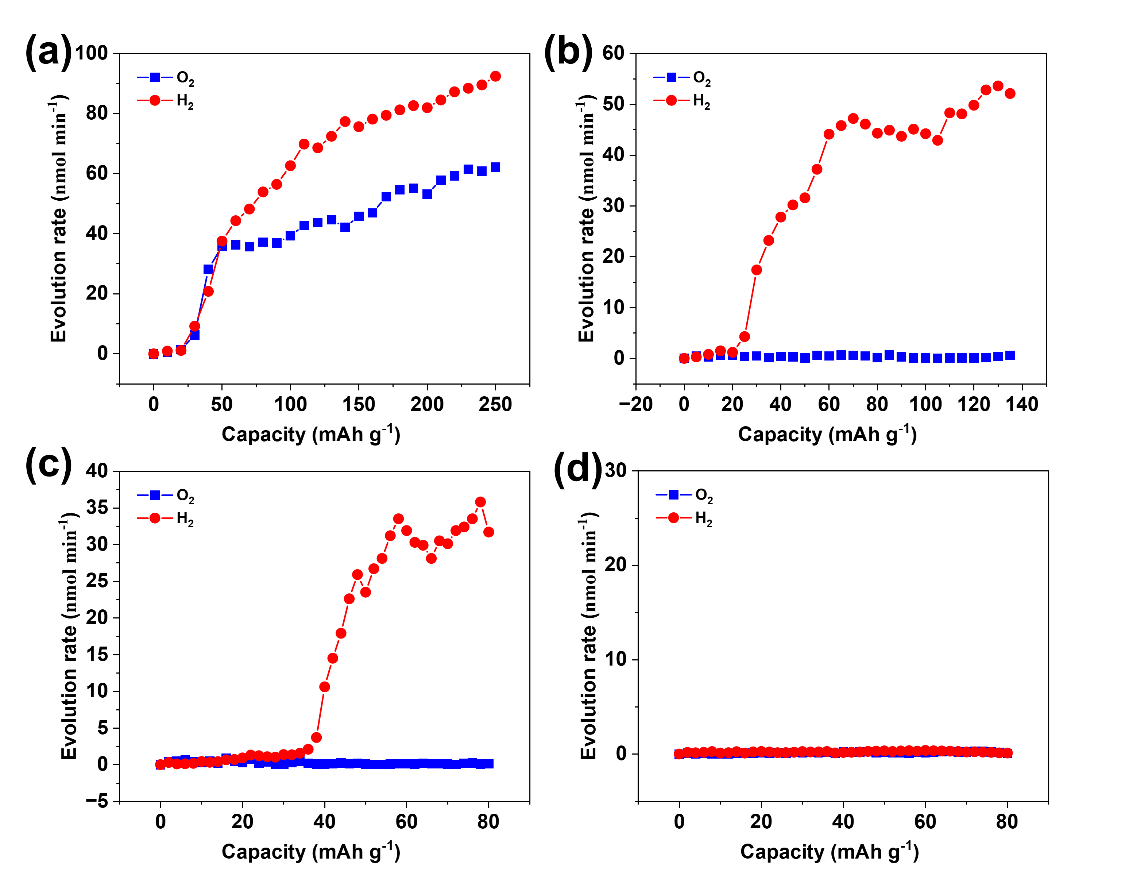


**Figure S42.** H_2_ and O_2_ evolution monitored from LMO/LTO full cells with 18 m LiMS-H_2_O (a), 3.0 m LiMS-TMP-H_2_O (b), 1.6 m LiMS-TMP-H_2_O (c) and 1.1 m LiMS-TMP-H_2_O (d) during the 151^st^ charging process after 150 cycles at 5C.


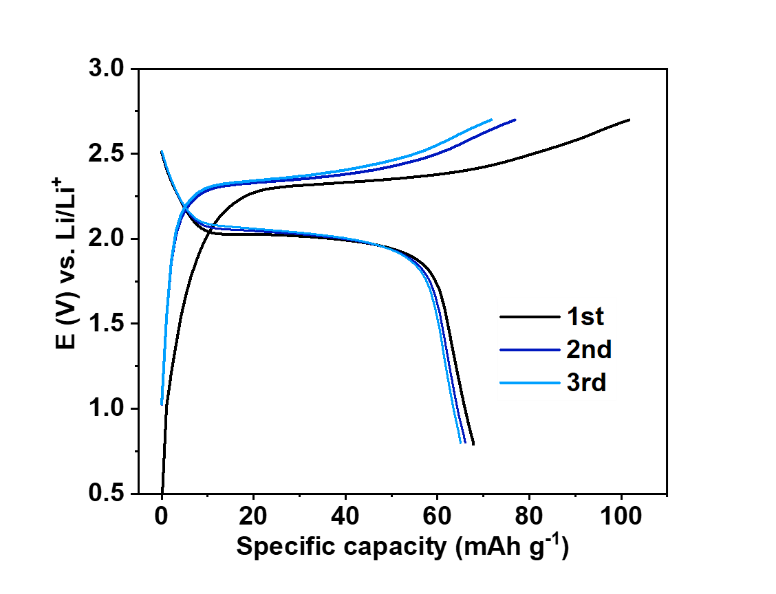


**Figure S43**. Voltage profile of LMO/TiO_2_ aqueous battery with 1.1 m LiMS-TMP-H_2_O electrolyte at 5 C.


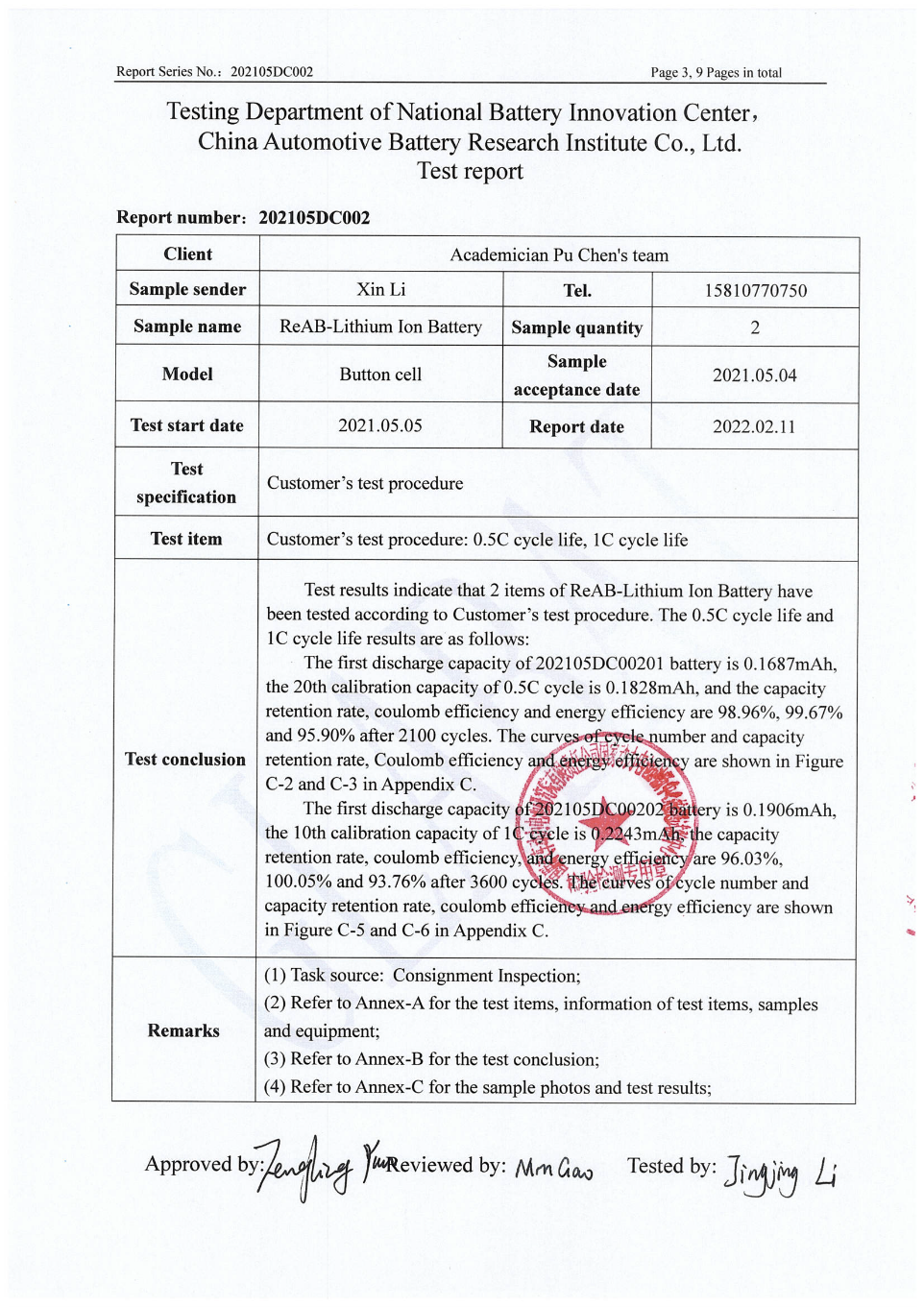


**Figure S44**. Certificate of 0.5 C and 1 C cycle life of LMO/LTP aqueous battery with 1.1 m LiMS-TMP-H_2_O electrolyte issued by China Automotive Battery Research institute.


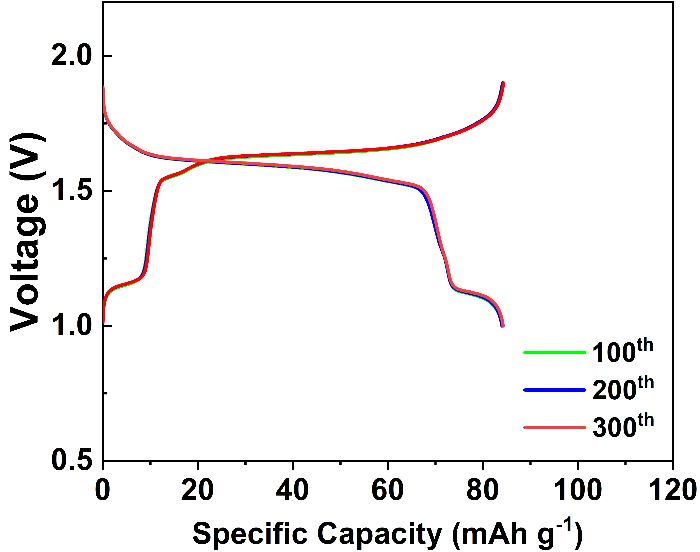


**Figure S45.** GCD profiles of LMO/LTP full cells at 0.5 C after 100th, 200th and 300th cycles.


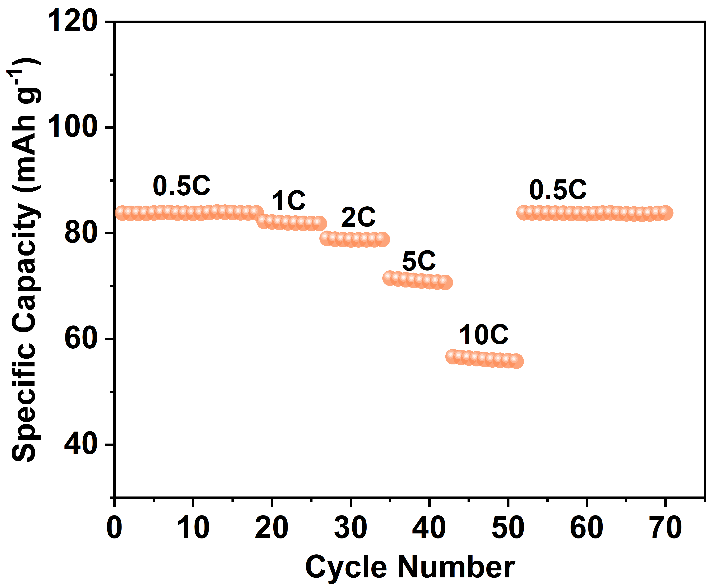


**Figure S46.** The rate performance of LMO/LTP battery employing 1.1 m LiMS-TMP-H_2_O electrolyte.


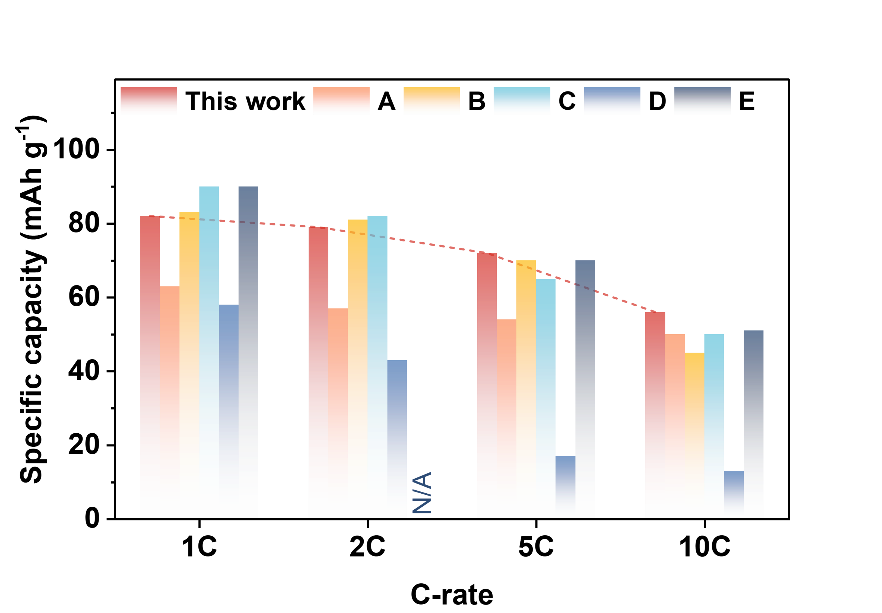


**Figure S47.** Comparison of rate performance of 1.1 m LiMS-TMP-H_2_O electrolyte in LMO/LTP electrode pair with other aqueous electrolytes (A, B, C, D and E refers to Ref.48 to 52 in main manuscript).


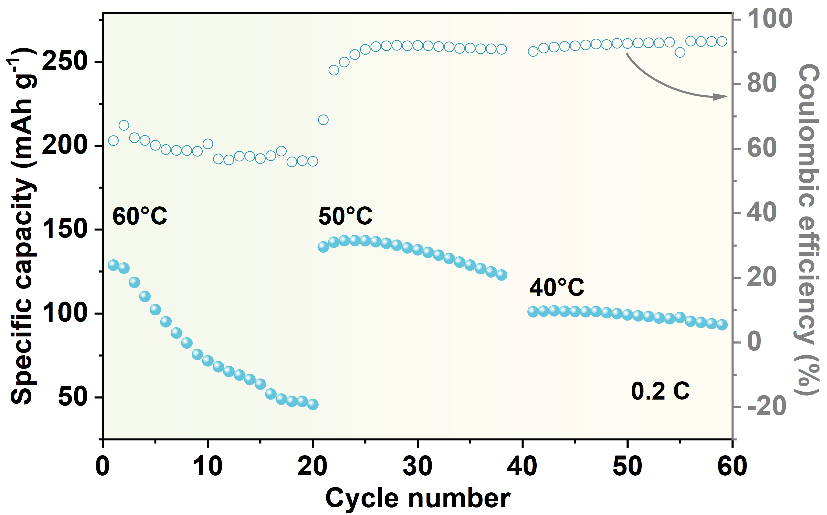


**Figure S48.** Cycling performance of LMO/LTP using 1.1 m LiMS-TMP-H_2_O electrolyte at 40-60℃.


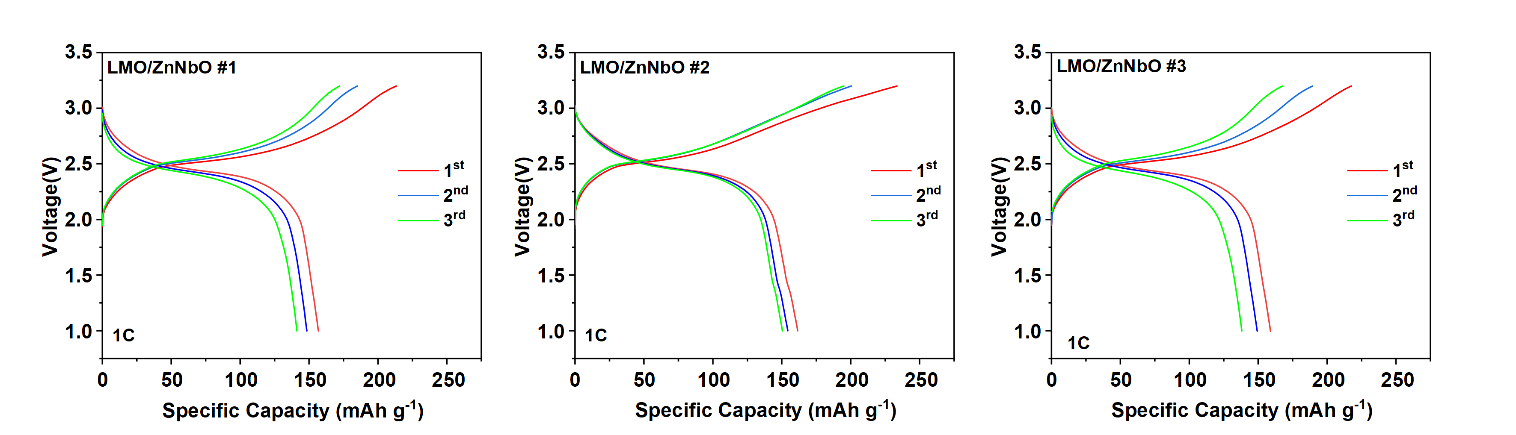


**Figure S49.** Voltage profiles of LMO/ZnNbO aqueous battery with 1.1 m LiMS-TMP-H_2_O electrolyte at 1 C.


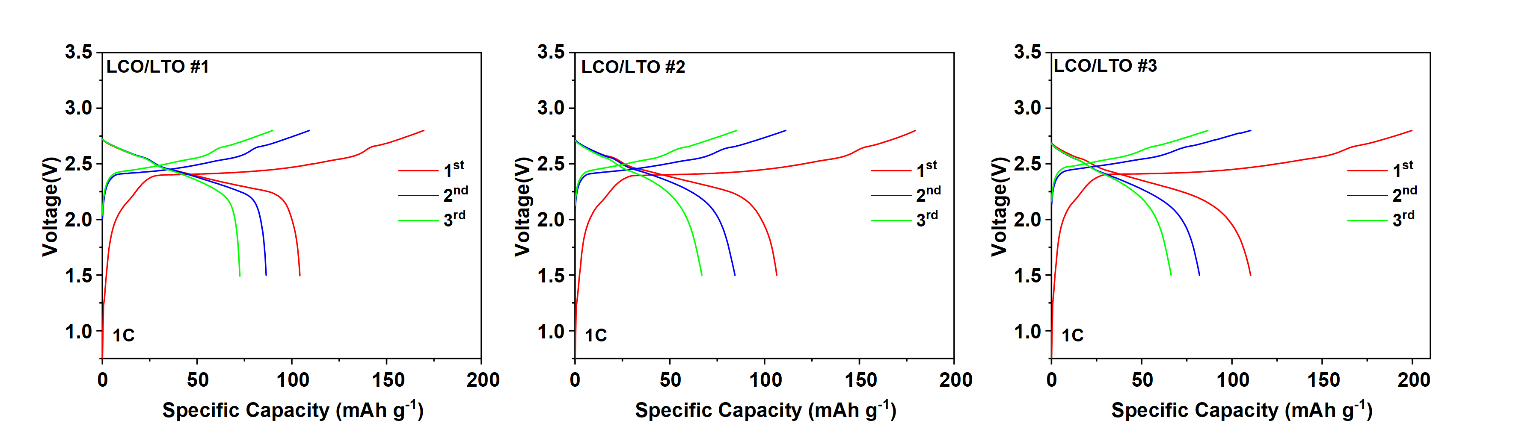


**Figure S50.** Voltage profiles of LCO/LTO aqueous battery with 1.1 m LiMS-TMP-H_2_O electrolyte at 1 C.


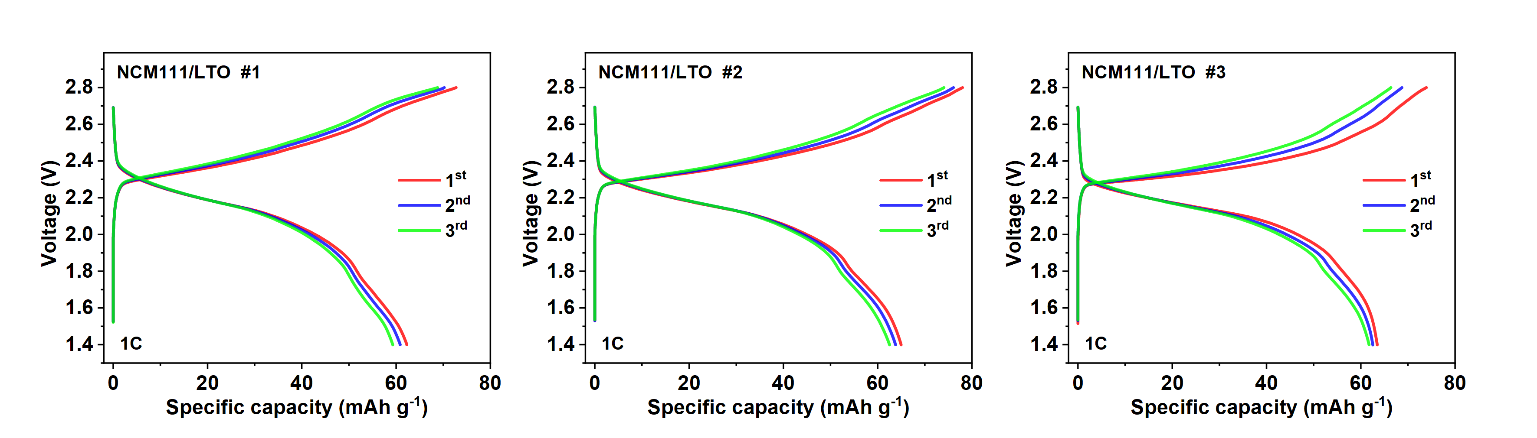


**Figure S51.** Voltage profiles of NCM_111_/LTO aqueous battery with 1.1 m LiMS-TMP-H_2_O electrolyte at 1 C.


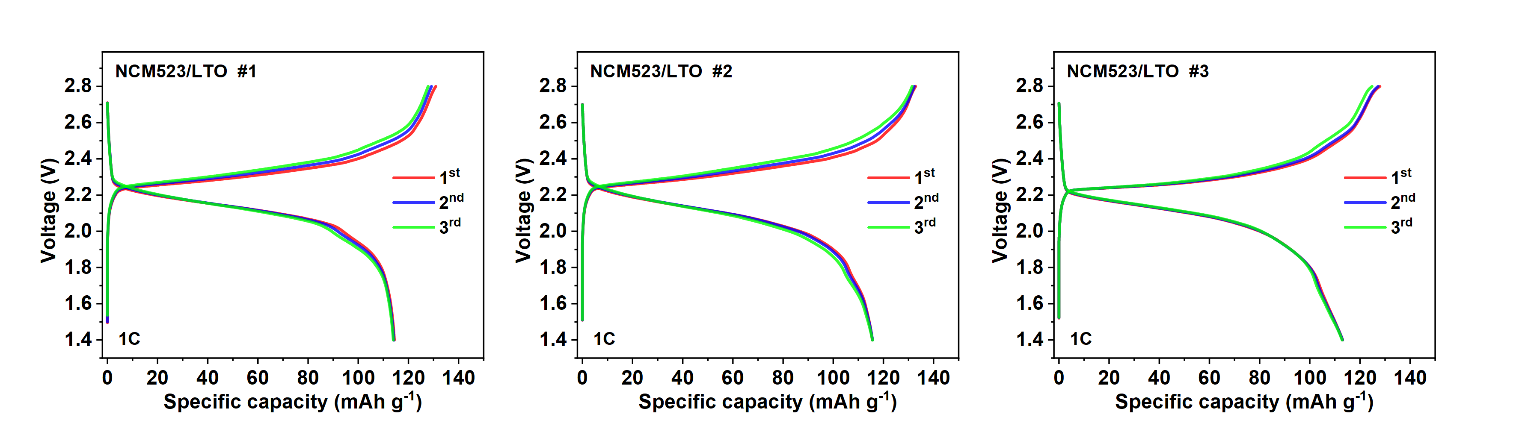


**Figure S52.** Voltage profiles of NCM_523_/LTO aqueous battery with 1.1 m LiMS-TMP-H_2_O electrolyte at 1 C.


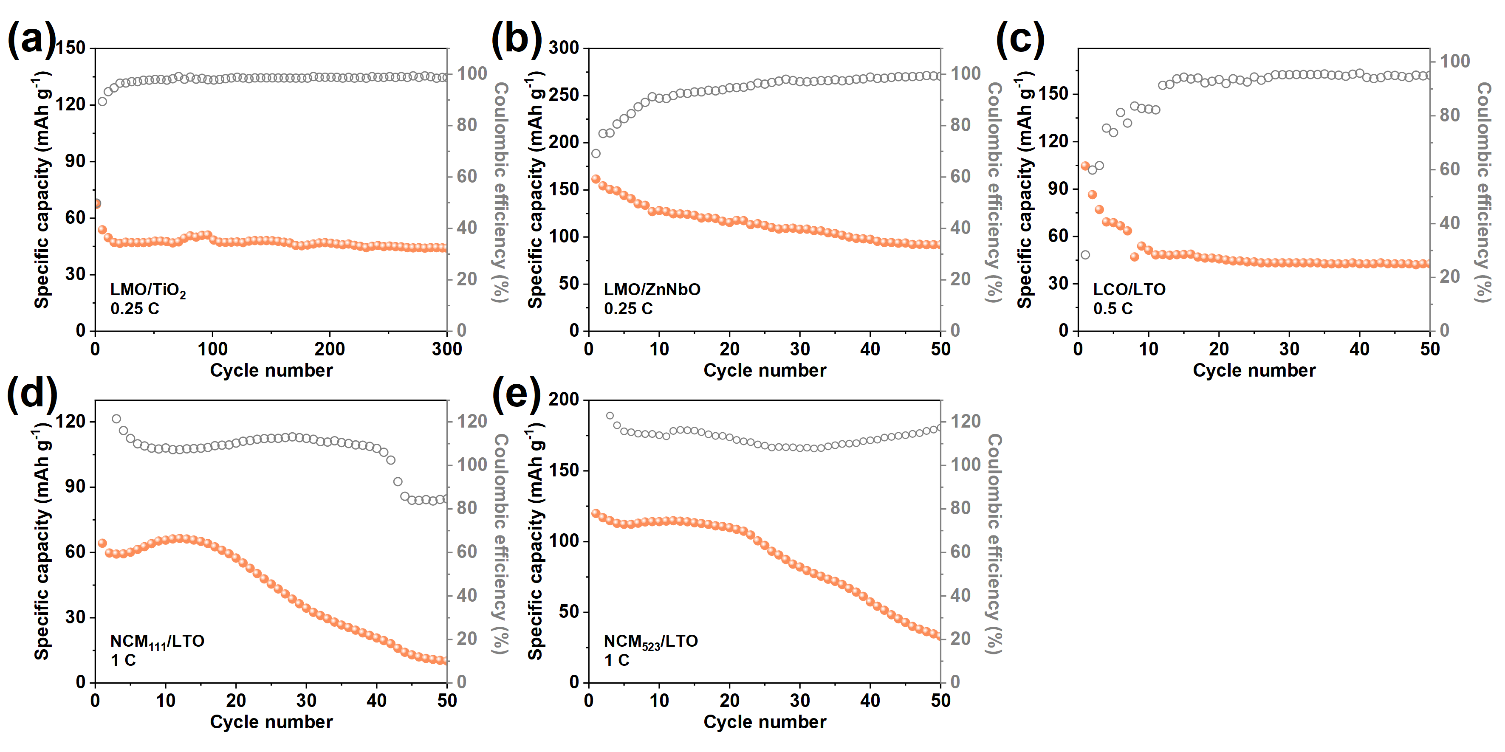


**Figure S53.** Cycling performance of (a) LMO/TiO_2_, (b) LMO/ZnNbO, (c) LCO/LTO, (d) NCM_111_/LTO and (e) NCM_523_/LTO full cells with 1.1 m LiMS-TMP-H_2_O electrolyte.


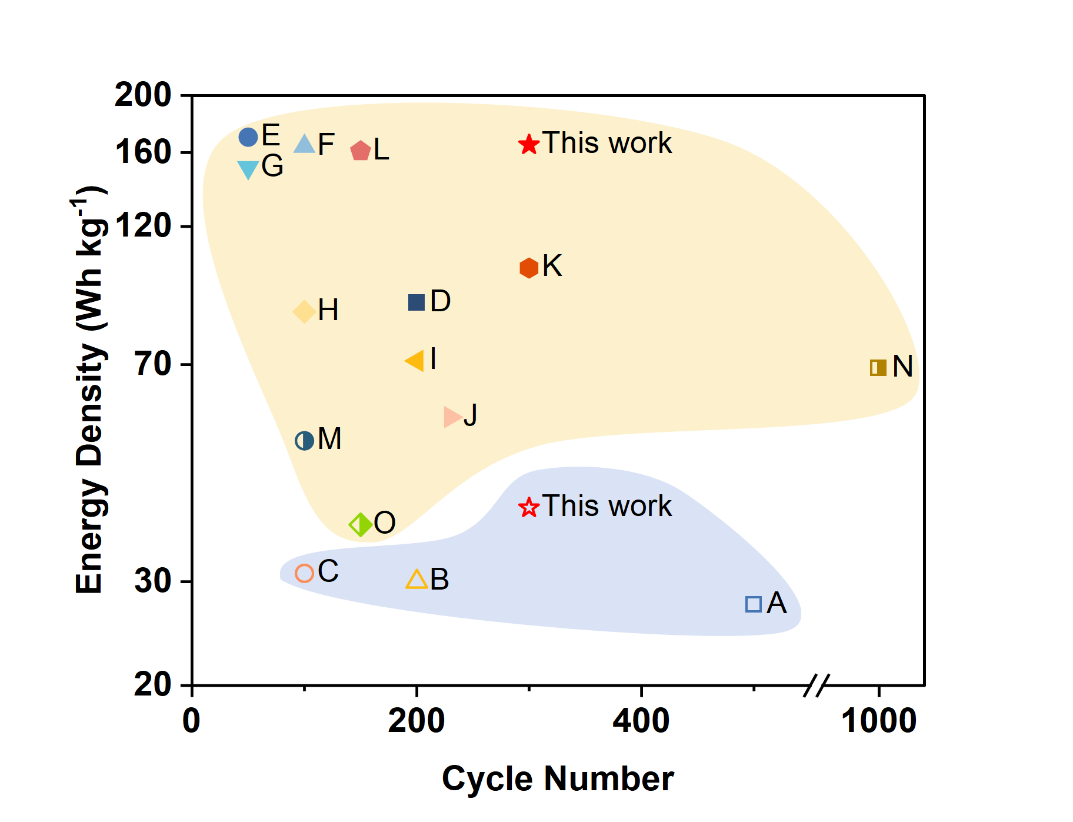


**Figure S54.** Energy density and cycle number comparison among this work and some state-of-the-art aqueous batteries. (hollow symbols A, B and C) are based on real gravimetric energy density, solid symbols D, E, F, G, H, I, J, K, L, M, N and O are based on the mass of active electrode materials). A-C refers to Ref. 57,61 and 62, while D-O refers to Ref. 63 to 74 in main manuscript.


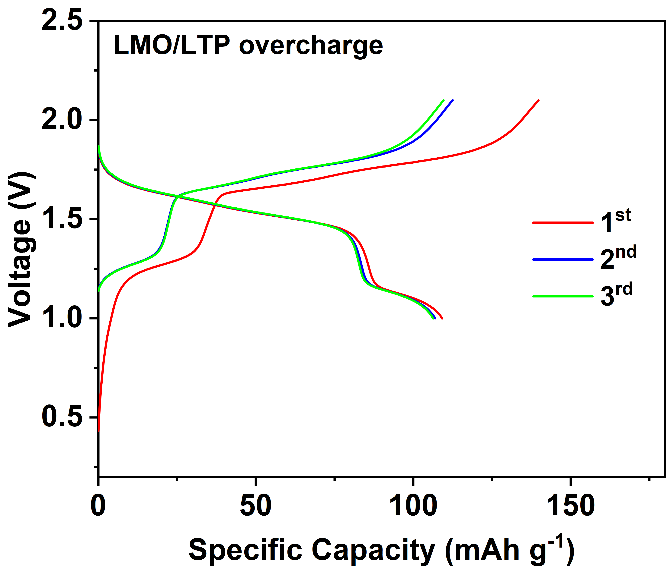


**Figure S55.** Voltage profiles of LMO/LTP aqueous battery with 1.1 m LiMS-TMP-H_2_O electrolyte overcharged to 2.5 V under 0.5 C.


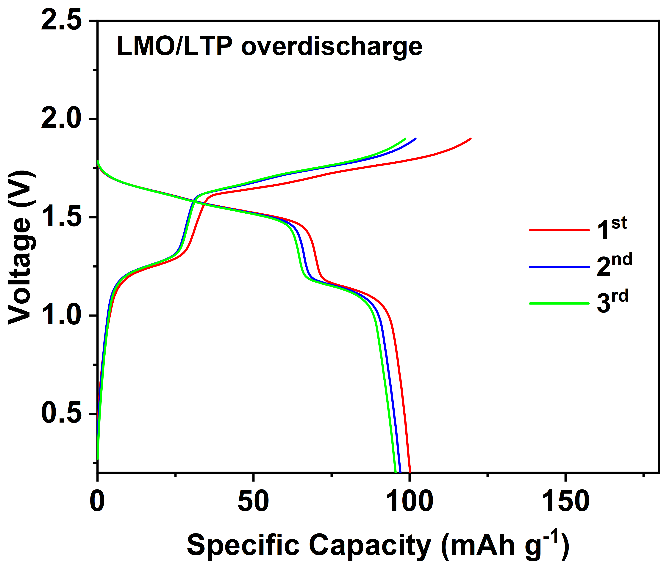


**Figure S56.** Voltage profiles of LMO/LTP aqueous battery with 1.1 m LiMS-TMP-H_2_O electrolyte overdischarged to 0 V under 0.5 C.


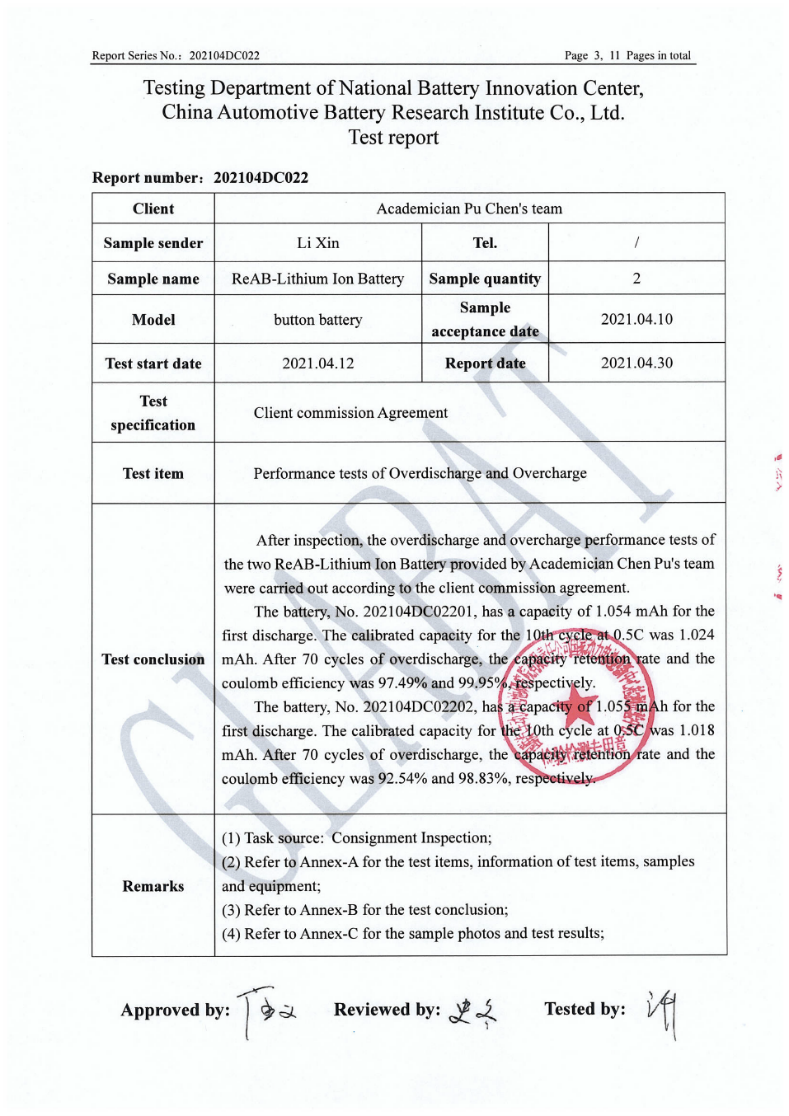


**Figure S57**. Certificate of overcharge/overdischarge performance of LMO/LTP aqueous battery with 1.1 m LiMS-TMP-H_2_O electrolyte issued by China Automotive Battery Research institute.


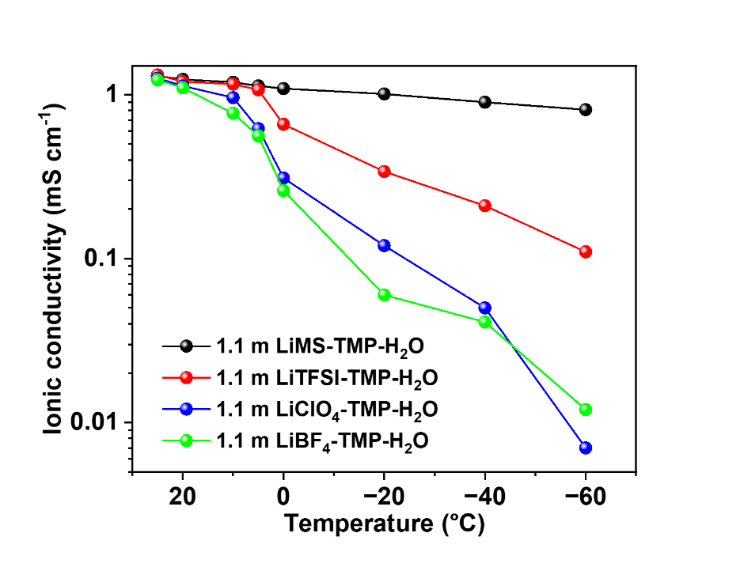


**Figure S58.** Ionic conductivities of 1.1 m LiMS-TMP-H_2_O, LiTFSI-TMP-H_2_O, LiClO_4_-TMP-H_2_O and LiBF_4_-TMP-H_2_O electrolytes over the temperature range of -60°C~+20°C.


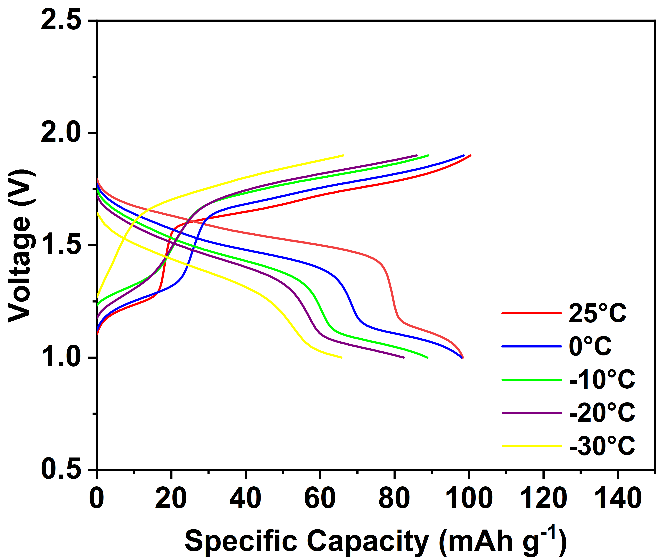


**Figure S59.** Voltage profiles of LMO/LTP aqueous battery with 1.1 m LiMS-TMP-H_2_O electrolyte under different temperatures.


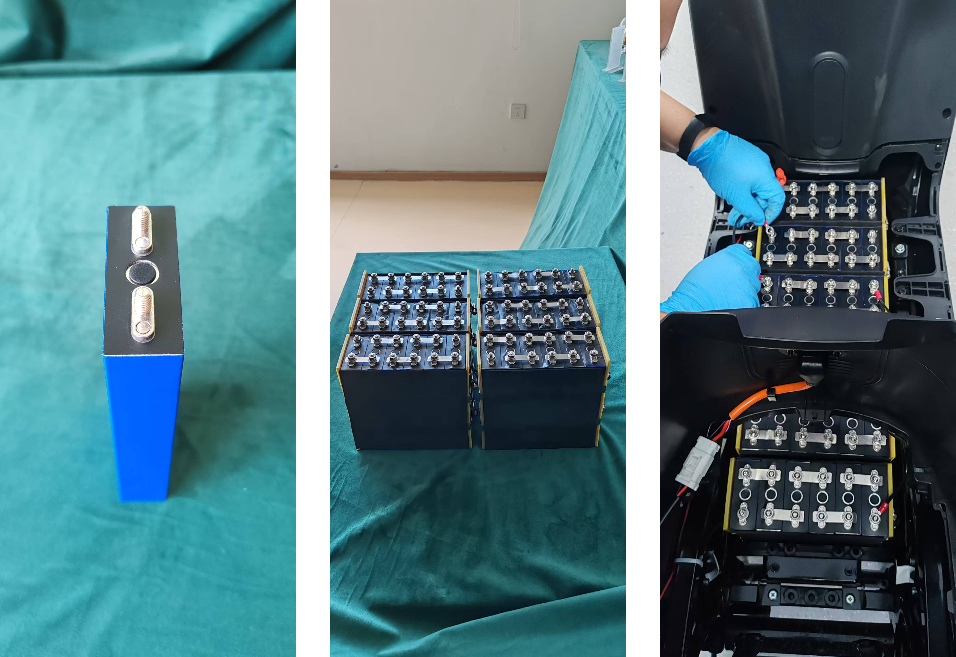


**Figure S60**. Assembly of commercially available 60V 15Ah LMO/LTP aqueous battery employing MIP electrolyte battery, which drives an electric bike to run smoothly for a demonstration distance of 70 km.


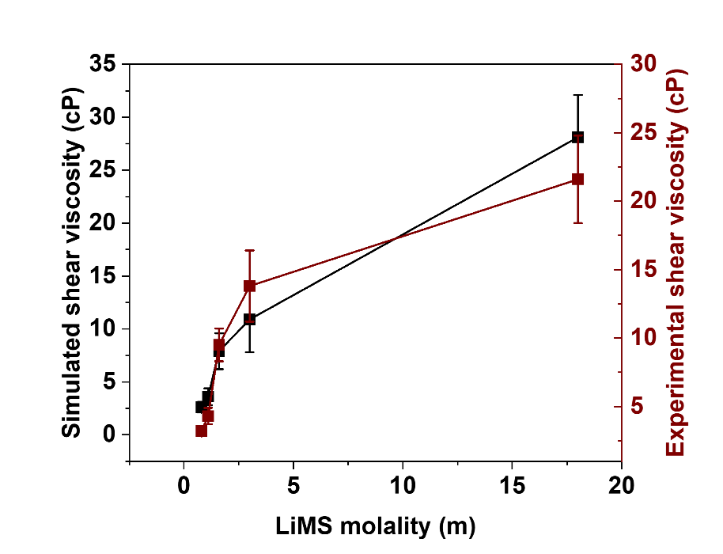


**Figure S61**. MD simulated and experimental shear viscosity of various LiMS-TMP-H_2_O electrolyte with different concentration of LiMS and TMP.

**Table S2**. Composition of various LiMS-TMP-H_2_O electrolyte.

| LiMS molality (mol kg^-1^) | LiMS mol% | H_2_O mol% | TMP mol% | TMP mass concentration  (g mL^-1^) |
| --- | --- | --- | --- | --- |
| 18 | 25 | 75 | 0 | 0 |
| 3 | 17 | 50 | 33 | 0.89 |
| 1.6 | 12.5 | 37.5 | 50 | 0.98 |
| 1.1 | 10 | 30 | 60 | 1.01 |
| 0.8 | 8.3 | 25 | 66.7 | 1.03 |

**Table S3.** The degree of ion uncorrelated motion (α) and the transference number of Li^+^ in different LiMS-TMP-H_2_O systems of MD simulations.

| Systems | degree of ion uncorrelated motion, α | Li^+^ transference number |
| --- | --- | --- |
| LiMS-H_2_O | 0.362± 0.008 | 0.5527±0.0551 |
| 3.0 m | 0.415± 0.004 | 0.5632±0.0767 |
| 1.6 m | 0.455± 0.008 | 0.5373±0.0521 |
| 1.1 m | 0.501± 0.006 | 0.6019±0.0557 |
| 0.8 m | 0.548± 0.011 | 0.5155±0.0501 |

**Table S4.** Electrochemical performance of recently reported aqueous metal-ion batteries.

| **Electrode material** | **Electrolyte** | **Current density** | **Energy density** | **Cycle number** | | | **Ref^1^** |
| --- | --- | --- | --- | --- | --- | --- | --- |
| N-ccLTP/LMO-GF | 2 M Li_2_SO_4_ | 0.5 C | 27.4 Wh kg^-1^ | | 500 cycles | A | |
| Zn_2_Nb_34_O_87_/LMO | 50 m LiTFSI-30 m TMBTFSI (WiBS) | 1 C | 30 Wh kg^-1^ | | 200 cycles | B | |
| Zn/Zn_0.25_V_2_O_5_ | Zn(OTf)_2_-H_2_O- sulfolane (RME) | 0.28 C | 31 Wh kg^-1^ | | 100 cycles | C | |
| NTP/NMF | NaClO_4_/NaOH | 0.5 C | 88.9 Wh kg^-1^ | | 200 cycles | D | |
| Al_2_O_3_@Li_4_Ti_5_O_12_/ LiVPO_4_F | 42 m LiTFSI-21 m Pyr_13_∙TFSI (WiSE) | 0.2 C | 170 Wh kg^-1^ | | 50 cycles | E | |
| TiO_2_/LiMn_2_O_4_ | 50 m LiTFSI-25 m TMBTFSI (WiS) | 1 C | 164 Wh kg^-1^ | | 100 cycles | F | |
| LTO/LMO | 12 m SAPE@SPE | 0.5 C | 151 Wh kg^-1^ | | 50 cycles | G | |
| NaTi_2_(PO_4_)_3_/ Na_2_MnFe(CN)_6_ | “water-in-polymer” gel electrolyte | 0.1 C | 86 Wh kg^-1^ | | 100 cycles | H | |
| NaTiOPO_4_/Na_1.88_Mn[Fe(CN)_6_]_0.97_ | 9 m NaOTF-22 m TEAOTF (IC-WiS) | 0.25 C | 71 Wh kg^-1^ | | 200 cycles | I | |
| TiO_2_-CsF/LMO | 13 m LiTFSI | 1.5 C | 57 Wh kg^-1^ | | 230 cycles | J | |
| LTO/NCM811 | LD_1.7_W_0.6_-KOH | 3 C | 102 Wh kg^-1^ | | 300 cycles | K | |
| NbO_2_/LMO | 2.6 m LiTFSI-H_2_O-MU | 0.25 C | 161 Wh kg^-1^ | | 150 cycles | L | |
| TiO_2_/LMO | 18 m LiTFSI | 0.27 C | 52 Wh kg^-1^ | | 100 cycles | M | |
| Zn/MnO_2_ | 2 M ZnSO_4_-20 mM PA-0.1 M MnSO_4_ | 4 C | 69 Wh kg^-1^ | | 1000 cycles | N | |
| Zn/VOX | ZnSO_4_/AEV deep eutectic electrolyte | 0.2 C | 37.46 Wh kg^-1^ | | 150 cycles | O | |
| This work | 1.1 m LiMS-TMP-H_2_O | 0.5 C | 30 Wh kg^-1^ | | 300 cycles | * | |
|  |  | 0.5 C | 165 Wh kg^-1^ | | 300 cycles |  |  |

^1^ A-O refers to the symbols in Figure S54 in supporting information

**Table S5**. A summary of major MD simulations.

| Systems | | LiMS:H_2_O:TMP |  | #LiMS | #H_2_O | #TMP | Initial box (Å^3^) | Atom No. | simulations time (ns) |
| --- | --- | --- | --- | --- | --- | --- | --- | --- | --- |
| LiMS-H_2_O | | 1:3:0 |  | 596 | 1796 | 0 | 60×60×60 | 10752 | 2000×3 |
| 3.0 m | | 1:3:2 |  | 210 | 644 | 420 | 60×60×60 | 10962 | 2000×3 |
| 1.6 m | | 1:3:4 |  | 134 | 412 | 536 | 60×60×60 | 11554 | 2000×3 |
| 1.1 m | | 1:3:6 |  | 99 | 296 | 594 | 60×60×60 | 11877 | 2000×3 |
| 0.8 m | | 1:3:8 |  | 80 | 240 | 640 | 60×60×60 | 12320 | 2000×3 |
|  | Small systems for PDOS calculations | | | | | | | | |
| LiMS-H_2_O | | 1:3:0 |  | 66 | 198 | 0 | 29×29×29 | 1188 | 500×3 |
| 1.1 m | | 1:3:6 |  | 11 | 33 | 66 | 29×29×29 | 1320 | 500×3 |
|  | γ- Li_3_PO_4_ and amorphous slab aqueous systems | | | | | | | | |
| Systems | | Li_3_PO_4_ size (Å^3^) |  | #H_2_O | #HCl | #LiCl | Initial box (Å^3^) | Atom No. | simulations time |
| γ-Li_3_PO_4_ | | 36.4×29.3×20.8 |  | 903 | 54 | 54 | 36.4×29.3×57 | 5229 | 200 ns×3 |
| γ- Li_3_PO_4_  vacancy diffusion | | 36.7×29.6×~20 |  | 903 | 54 | 54 | 36.7×29.6×49 | 5229 | (100 ps×10)×5 |
| γ- Li_3_PO_4_  vacancy diffusion  at 500K/600K | | 36.7×29.6×~20 |  | 903 | 54 | 54 | 36.7×29.6×49 | 5229 | (100 ps×10)×2 |
| amorphous Li_3_PO_4_ | | 36.4×29.3×~23 |  | 903 | 54 | 54 | 36.4×29.3×57 | 5229 | 200 ns×3 |
| amorphous Li_3_PO_4_  vacancy diffusion | | 36×29×~23 |  | 903 | 54 | 54 | 36×29×50.8 | 5229 | (100 ps×10)×5 |

**Table S6**. Bonded and nonbonded parameters used for PO_4_^3-^ and H^+^ of Li_3_PO_4_ in MD simulations.

| Nonbonded parameters | | | |
| --- | --- | --- | --- |
|  | R_min_/2 (Å) ^a^ | epsilon (kcal mol^-1^) | q (e) |
| O | 1.8401 | 0.21 | -1.4 |
| P | 2.1 | 0.2 | 2.6 |
| H^+^ | 0.841 | 0.00001138 | 1 |
| Bonded parameters | | | |
| BOND | *K*_b_ (kcal/mol) | *r*_0_ (Å) |  |
| P-O | 525 | 1.53 |  |
| ANGLE | *K*_θ_ (kcal/mol/rad^2^) | *θ*_0_ (°) |  |
| O-P-O | 525 | 109.5 |  |

^a^ R_min_ is the distance of two atoms at Lennard-Jones potential minimum.

**Table S7.** Calculated gas-phase Gibbs free energies and solvation free energies at 298 K in water and acetone solvation models.^a^

|  | water | | acetone | |
| --- | --- | --- | --- | --- |
|  | (CH_3_)_3_PO_4_ | (CH_3_)_2_PO_4_^-^ +·CH_3_ | (CH_3_)_3_PO_4_ | (CH_3_)_2_PO_4_^-^ +·CH_3_ |
| G_gas_ | -761.2428055 | -761.250006 | -761.2431672 | -761.2548541 |
| ΔG_solv_ | -0.011274423 | -0.114068005 | -0.01215059 | -0.084501998 |
| G_solv_ | -761.251068 | -761.3610621 | -761.2523059 | -761.3363442 |
| ΔG_298 K,solv_ (without Li^+^) | | -0.1099941 |  | -0.0840383 |
|  | water | | acetone | |
|  | (CH_3_)_3_PO_4_Li | (CH_3_)_2_PO_4_Li -CH_3_ | (CH_3_)_3_PO_4_Li | (CH_3_)_2_PO_4_Li -CH_3_ |
| G_gas_ | -768.7440138 | -768.8503315 | -768.7467751 | -768.8499609 |
| ΔG_solv_ | -0.005600416 | -0.096178564 | -0.005177482 | -0.086575973 |
| G_solv_ | -768.7466023 | -768.9434982 | -768.7489407 | -768.933525 |
| ΔG_298 K,solv_ (with Li^+^) | | -0.1968958 |  | -0.1845843 ^b^ |

^a^ All values are in Hartrees. G_gas_ includes the G_coor_ correction term; G_solv_= G_gas_ + ΔG_solv_+1.89/627.51, where 1.89 kcal/mol is the ΔG ^1 atm → 1 M^ as the free energy of compression from 1 atm to 1 M.

^b^ The reduction potential, for example for (CH_3_)_3_PO_4_Li, is calculated as -(ΔG_298 K,solv_/*nF*) - 1.4 = -(-0.1845843 ×627.5095×1000×4.184/96485.33212) -1.4 =3.62 V (vs. Li/Li^+^)
